# A rank-conditioned *α*-stable perturbation for differential evolution: Separating perturbation frequency and tail heaviness

**DOI:** 10.1371/journal.pone.0357907

**Published:** 2026-09-09

**Authors:** Tae Jong Choi, Yeji An

**Affiliations:** 1 Graduate School of Data Science, Chonnam National University, Gwangju, Republic of Korea; 2 Department of Lifelong Education, Kyungil University, Gyeongsangbuk-do, Republic of Korea; Gulf University for Science and Technology, KUWAIT

## Abstract

Heavy-tailed perturbations are commonly used in differential evolution (DE) to restore diversity and mitigate premature convergence. Adaptive Cauchy perturbation refines this strategy by varying perturbation probabilities according to dimension-wise convergence, but it retains a fixed Cauchy law and cannot adjust perturbation severity across individuals. This paper proposes rank-conditioned α-stable perturbation, a two-level adaptive operator that decouples perturbation activation from tail-shape control. Dimension-wise convergence indicators determine coordinate-specific perturbation probabilities, while each individual’s fitness rank determines the stability index of a symmetric α-stable kernel. Thus, higher-ranked individuals receive lighter-tailed perturbations for local refinement, whereas lower-ranked individuals receive heavier-tailed perturbations for occasional long-range exploration. Unlike fixed-kernel heavy-tailed DE operators, the proposed operator induces a per-individual exploration–exploitation trade-off within each generation. It is embedded into L-SRTDE, the winner of the IEEE CEC 2024 competition, and evaluated on the IEEE CEC 2017 benchmark suite across multiple dimensions. Results show the best Friedman rankings among the compared methods and favorable Wilcoxon outcomes against recent DE variants. Controlled comparisons with conventional Cauchy and adaptive dimension-wise Cauchy perturbations show that rank-guided tail-shape adaptation complements probability-based activation, with modest overhead and stable performance across α-stable parameter settings.

## Introduction

We consider bound-constrained continuous optimization problems of the form:


$$\begin{array}{c} \min_{\vec{x}\in \Omega }\;f(\vec{x}), \end{array}$$ (1)

where $\vec{x}=(x_{1},...,x_{d})\in {\mathbb{R}}^{d}$ is a decision vector, $\Omega \subseteq {\mathbb{R}}^{d}$ is the feasible domain, and $f:{\mathbb{R}}^{d}\to \mathbb{R}$ is the objective function. A global minimizer ${\vec{x}}^{⋆}\in \Omega$ satisfies $f({\vec{x}}^{⋆})\leq f(\vec{x})$ for all $\vec{x}\in \Omega$ [1,2].

In many applications, f(·) can only be accessed through pointwise evaluations. The objective value may be produced by simulations, measurements, or proprietary models for which derivative information is unavailable, unreliable, or computationally expensive. Such black-box settings are typically subject to a strict evaluation budget *B*, and the practical goal is to return a high-quality solution $\hat{\vec{x}}\in \Omega$ within at most *B* function evaluations. These constraints motivate derivative-free, population-based optimizers that can balance exploration and exploitation without analytic information about f(·) [3,4].

Differential evolution (DE) [5] is a widely used population-based method for continuous black-box optimization. By combining differential mutation, crossover, and greedy selection, DE often performs well on nonconvex and multimodal landscapes [6]. Nevertheless, DE is susceptible to diversity loss: the population may contract prematurely, causing the search to become increasingly local and stagnate in suboptimal regions [6]. This effect is particularly important under limited evaluation budgets, because the algorithm may spend a large fraction of its remaining evaluations refining an unpromising basin.

A common strategy for mitigating premature convergence is to introduce heavy-tailed perturbations. Since heavy-tailed distributions assign non-negligible probability to large deviations, they can occasionally generate long-range moves and help the population escape local optima. Cauchy perturbation (CP) [7] is a representative example. During recombination, selected components of a target vector are replaced by Cauchy-distributed samples centered at the target coordinate. This mechanism can reintroduce diversity without redesigning the entire DE framework. However, conventional CP applies a fixed perturbation law once perturbation is activated. It therefore does not account for two forms of heterogeneity: different coordinates may converge at different rates, and different individuals may play different search roles within the population.

Adaptive Dimension-wise Cauchy Perturbation (ADCP) [8] addresses the first form of heterogeneity by adapting the perturbation probability at the coordinate level. Specifically, ADCP estimates the convergence level of each dimension from population spread and maps this information to a dimension-specific jumping probability. Perturbations are thereby concentrated on coordinates that exhibit stronger premature convergence, while dimensions that remain diverse are disturbed less frequently. However, ADCP still uses a fixed Cauchy kernel after perturbation is activated. Thus, it adapts where perturbations are applied, but not how heavy-tailed or disruptive the perturbation should be.

This remaining limitation motivates the present study. In a population-based optimizer, high-quality individuals are often more useful for local refinement around promising regions, whereas low-quality individuals can be used more aggressively to explore distant areas. A fixed perturbation law treats these individuals identically once perturbation is activated, which may be unnecessarily disruptive for elite solutions and insufficiently exploratory for weaker solutions in difficult multimodal landscapes. We therefore formulate heavy-tailed perturbation as a two-level control problem: coordinate-wise activation determines where perturbations are applied, while individual-wise tail-shape control determines the degree of perturbation severity.

In this study, we propose *Adaptive Lévy Perturbation* (ALP), a rank-conditioned α-stable perturbation mechanism for DE. ALP preserves the dimension-wise jumping schedule of ADCP [8], but replaces the fixed Cauchy draw with a symmetric α-stable perturbation whose stability index is assigned according to fitness rank. Since the stability index α controls the tail behavior of an α-stable distribution, ALP assigns lighter-tailed perturbations to high-quality individuals and heavier-tailed perturbations to low-quality individuals. In this way, the proposed method decouples coordinate-level perturbation activation from individual-level perturbation severity.

We embed ALP into L-SRTDE [9], a recent success-rate-based DE framework that won the IEEE CEC 2024 competition, and evaluate the resulting algorithm on the IEEE CEC 2017 benchmark suite [10] across multiple dimensional settings. Although the suite was originally introduced for the 2017 competition, it remains a widely used reference benchmark in recent CEC-related single-objective optimization research and competition-level algorithm assessment. Experimental results indicate that L-SRTDE-ALP obtains the best overall Friedman rankings among the compared algorithms and favorable Wilcoxon outcomes against several recent DE variants. Controlled comparisons with CP and ADCP further suggest that adapting the perturbation distribution itself complements probability-based perturbation activation.

The main contributions of this study are summarized as follows:

- We formulate heavy-tailed perturbation in DE as a two-level control problem that separates coordinate-wise perturbation activation from individual-wise perturbation tail-shape control.
- We propose ALP, a rank-conditioned symmetric α-stable perturbation mechanism. By assigning stability indices according to fitness rank, ALP provides lighter-tailed perturbations for high-quality individuals and heavier-tailed perturbations for low-quality individuals.
- We integrate ALP into the L-SRTDE framework while preserving the baseline mutation, success-rate feedback, population management, and selection mechanisms. This allows the effect of rank-conditioned tail adaptation to be evaluated as a modular recombination-level enhancement.
- We conduct an extensive empirical study on the IEEE CEC 2017 benchmark suite across multiple dimensions, including comparisons with state-of-the-art DE variants and controlled comparisons with CP and ADCP.

The remainder of this study is organized as follows. The Preliminaries section reviews background material on DE, Cauchy perturbation, ADCP, and α-stable distributions. The Literature Review section summarizes related research, with particular emphasis on L-SHADE-family variants and perturbation-based diversity enhancement mechanisms. The Proposed Algorithm section presents the proposed ALP operator and its integration into L-SRTDE. The Experimental Study section describes the experimental protocol and discusses the results. Finally, the Conclusion section concludes the paper and outlines directions for future research.

## Preliminaries

### DE

DE [5] is a real-coded evolutionary algorithm designed for continuous black-box optimization. At generation *g*, DE maintains a population ${𝒫}^{g}=\{{\vec{x}}_{1}^{\;g},...,{\vec{x}}_{N}^{\;g}\}\subseteq \Omega \subseteq {\mathbb{R}}^{d}$ which evolves through the iterative generation of trial vectors followed by greedy selection.

#### Mutation.

Mutation constructs a mutant vector by scaling the difference between population members. For example, the classical DE/rand/1 strategy generates, for each target index *i*,


$$\begin{array}{c} {\vec{v}}_{i}^{\;g}={\vec{x}}_{r_{1}}^{\;g}+F({\vec{x}}_{r_{2}}^{\;g}-{\vec{x}}_{r_{3}}^{\;g}), \end{array}$$ (2)

where $r_{1},r_{2},r_{3}$ are mutually distinct indices sampled uniformly from {1,...,N} (typically excluding *i*), and *F* > 0 denotes the scaling factor.

#### Crossover.

The mutant vector ${\vec{v}}_{i}^{\;g}$ is then recombined with the target vector ${\vec{x}}_{i}^{\;g}$ to form a trial vector ${\vec{u}}_{i}^{\;g}$. Under binomial crossover,


$$\begin{array}{c} u_{i,j}^{\;g}=\left\{ \begin{array}{cc} v_{i,j}^{\;g}, & \text{if }r_{i,j}\leq CR\text{ or }j=j_{rand},\\ x_{i,j}^{\;g}, & \text{otherwise,} \end{array}\right. \end{array}$$ (3)

where CR∈[0,1], $r_{i,j}\sim U(0,1)$, and $j_{rand}\in \{1,...,d\}$ ensures that at least one coordinate is inherited from the mutant.

#### Selection.

DE employs a one-to-one greedy selection mechanism:


$$\begin{array}{c} {\vec{x}}_{i}^{\;g+1}=\left\{ \begin{array}{cc} {\vec{u}}_{i}^{\;g}, & \text{if }f({\vec{u}}_{i}^{\;g})\leq f({\vec{x}}_{i}^{\;g}),\\ {\vec{x}}_{i}^{\;g}, & \text{otherwise.} \end{array}\right. \end{array}$$ (4)

This process is repeated until the evaluation budget is exhausted or another stopping criterion is satisfied.

#### Extensions and applications of DE.

DE offers a flexible and modular framework for continuous optimization, and many effective variants have been proposed by modifying or augmenting its operators, hybridization mechanisms, and application-specific modeling. Recent work includes dynamic population structuring, where the population is decomposed into multiple subpopulations and the number of subpopulations is adjusted online using clustering analysis to better balance exploration and exploitation [11]. Hybrid metaheuristics have also been explored, such as dual-population frameworks that split the population into two groups evolved by different paradigms and then recombine them each iteration, leveraging complementary exploration–exploitation behaviors to improve robustness and solution quality in large-scale nonconvex dispatch problems [12]. Beyond structural changes and hybridization, improvements have been developed at the operator level. For instance, an improved crossover strategy can generate trial vectors by combining information from original individuals and multiple mutated candidates, enhancing diversity and reducing stagnation; together with adaptive parameter control and constraint-handling repair, this leads to stronger performance on benchmark suites and complex constrained dispatch tasks [13]. DE has also been embedded into hybrid optimization pipelines for practical engineering design, such as integrating an improved PSO with periodic DE steps and multi-direction local search to optimize constrained layout objectives, demonstrating the usefulness of DE-based hybrids in real-world systems with complex evaluation models [14]. Comprehensive overviews of recent advances and representative DE families can be found in the survey literature [6,15,16].

### Cauchy perturbation and adaptive dimension-wise cauchy perturbation

Heavy-tailed perturbation strategies are frequently employed in DE to mitigate diversity loss by enabling occasional non-local moves during recombination. In the following, we briefly review CP and its adaptive extension, ADCP, which serve as the conceptual baseline for this study.

#### Cauchy perturbation (CP).

CP [7] extends binomial crossover by allowing coordinates that would otherwise be inherited from the target vector to be replaced with samples drawn from a Cauchy distribution centered at the target. Let $J_{i}$ denote a Bernoulli random variable indicating whether the perturbation mode is activated for the individual *i*, i.e., $J_{i}=1$ if $r_{i}^{\left(2\right)}\leq JR$ and $J_{i}=0$ otherwise, where $r_{i}^{\left(2\right)}\sim U(0,1)$. Then, the CP trial vector is


$$\begin{array}{c} u_{i,j}^{\;g}=\left\{ \begin{array}{cc} v_{i,j}^{\;g}, & \text{if }r_{i,j}^{\left(1\right)}\leq CR\text{ or }j=j_{rand},\\ rndc\;\left(x_{i,j}^{\;g},\;0.1\right), & \text{if }r_{i,j}^{\left(1\right)}>CR\text{ and }J_{i}=1,\\ x_{i,j}^{\;g}, & \text{otherwise,} \end{array}\right. \end{array}$$ (5)

where $r_{i,j}^{\left(1\right)}\sim U(0,1)$ and rndc(μ,γ) denotes a Cauchy random variable with location μ and scale γ. Note that $J_{i}$ is shared across all coordinates of the individual *i*. Thus, once the perturbation is activated, all coordinates not inherited from the mutant are replaced by Cauchy draws.

#### Dimension-wise adaptation (ADCP).

A limitation of using a single global *JR* is that contraction can occur unevenly across coordinates. ADCP [8] addresses this by computing a convergence indicator for each dimension and converting it into a dimension-specific jumping probability. The convergence level of the coordinate *j* at generation *g* is defined as


$$\begin{array}{c} CL_{j}^{g}=1-\frac{\sigma_{j}^{g}}{\sigma_{j}^{0}}, \end{array}$$ (6)

where $\sigma_{j}^{g}$ is the current population standard deviation in dimension *j* and $\sigma_{j}^{0}$ is the initial value. To map $CL_{j}^{g}$ to a jumping rate, ADCP uses


$$\begin{array}{c} JR_{j}^{\;g}=JR_{\min}+(JR_{\max}-JR_{\min})\cdot \frac{e^{\kappa \;CL_{j}^{g}}-1}{e^{\kappa }-1}, \end{array}$$ (7)

where $JR_{\min},JR_{\max}\in [0,1]$ set the bounds for the probability, and κ>0 controls the nonlinearity. Using $JR_{j}^{\;g}$, the recombination rule becomes


$$\begin{array}{c} u_{i,j}^{\;g}=\left\{ \begin{array}{cc} v_{i,j}^{\;g}, & \text{if }r_{i,j}^{\left(1\right)}\leq CR\text{ or }j=j_{rand},\\ rndc\;\left(x_{i,j}^{\;g},\;0.1\right), & \text{if }r_{i,j}^{\left(1\right)}>CR\text{ and }r_{i,j}^{\left(2\right)}\leq JR_{j}^{\;g},\\ x_{i,j}^{\;g}, & \text{otherwise,} \end{array}\right. \end{array}$$ (8)

where $r_{i,j}^{\left(2\right)}\sim U(0,1)$ is sampled independently for each coordinate. This per-dimension decision concentrates heavy-tailed perturbations on coordinates exhibiting stronger contraction, while leaving other coordinates relatively stable. Since the perturbation kernel remains fixed to the Cauchy distribution, ADCP primarily controls where perturbations occur (through $JR_{j}^{g}$) rather than how heavy-tailed they are. This motivates the rank-guided Lévy α-stable mechanism introduced in the Proposed Algorithm section.

## Literature review

Recent developments in DE have pursued several complementary directions, including adaptive control of algorithm parameters, population-size and stage-dependent search regulation, rank-aware selective pressure, diversity management, and heavy-tailed variation mechanisms. Because the proposed method is implemented within L-SRTDE [9], we first review representative developments in the L-SHADE lineage [17] and closely related adaptive DE frameworks. We then discuss Lévy-based and heavy-tailed variation mechanisms, which provide the conceptual background for the proposed rank-conditioned α-stable perturbation.

### Success-history parameter adaptation

JADE [18] popularized the DE/current-to-pbest/1 mutation strategy together with an external archive and online adaptation of *F* and *CR*, providing a foundation for subsequent memory-based schemes. SHADE [19] extended this idea by maintaining a finite memory of successful parameter settings and sampling new control parameters from distributions centered on these memories. L-SHADE [17] further combined success-history adaptation with linear population size reduction (LPSR), enabling a gradual transition from broader exploration in the early search to stronger exploitation in later stages.

### Population reduction and stage-dependent control

Several advanced variants refine how parameter adaptation interacts with different stages of the search. For example, jSO [20] extends the L-SHADE framework with additional time-varying control rules that encourage exploration during the early stage and increasingly emphasize exploitation as the search progresses, yielding strong performance on CEC benchmark suites.

### Selective pressure and rank-aware sampling

Another line of research regulates how individuals contribute to offspring generation. LSHADE-RSP [21] introduces rank-based selective pressure, assigning higher-ranked solutions greater probabilities of participating in selected mutation roles. NL-SHADE-RSP [22] further combines this rank-aware mechanism with modified population reduction and archive management to improve the exploration–exploitation balance.

### Refinements of memory updates and bias control

NL-SHADE-LBC [23] modifies historical parameter adaptation by introducing controlled changes in the bias of memory updates. This enables the search to favor more aggressive or conservative parameter values depending on its current stage.

### Multi-population and ensemble extensions

L-NTADE [24] employs a dual-population design that separates newly generated individuals from high-quality solutions, allowing recent improvements to influence the search while preserving strong solutions as stable references. Ensemble-style approaches, such as mLSHADE-RL [25], combine multiple operators and may incorporate restart or local-search mechanisms to mitigate stagnation and improve robustness.

### Success-rate feedback control

Rather than relying exclusively on success-history memories, L-SRTDE [9] uses the observed generation-wise success rate as a feedback signal to regulate mutation intensity and elite selection while retaining linear population size reduction. This success-rate-based control mechanism provides the baseline framework into which the proposed perturbation operator is incorporated.

### Lévy-based and heavy-tailed variation

Adaptive variation mechanisms have also been extensively studied as a means of regulating exploration and exploitation. In evolution strategies, adaptation of mutation-related strategy parameters plays a central role in controlling search behavior [26]. Similar adaptive principles have been widely investigated in DE [6]. Heavy-tailed distributions provide a complementary mechanism because they generate predominantly moderate variations while retaining a non-negligible probability of producing large exploratory moves.

Several studies have incorporated Lévy-based mechanisms into DE in different forms. He and Yang [27] proposed Lévy DE (LDE), in which Lévy distributions are used for adaptive parameter control; in particular, the mutation scale factor $F_{i}$ is generated from different Lévy distributions according to their historical performance. Sharma et al. [28] introduced Lévy Flight DE (LFDE), where Lévy flight is employed as an additional local-search mechanism around promising solutions. More recently, Civicioglu and Besdok [29] proposed Bernstein–Lévy differential evolution, which combines a Lévy-based search mechanism with elitist mutation and a stochastic Bernstein-polynomial crossover strategy. These studies illustrate that Lévy and other heavy-tailed mechanisms can be incorporated into DE through parameter generation, auxiliary local search, or specialized variation operators.

The proposed ALP differs from these approaches in the quantity being adapted. It does not use a Lévy distribution to generate a conventional DE control parameter such as *F*, nor does it introduce a separate Lévy-flight local-search phase. Instead, ALP directly adapts the tail shape of the perturbation distribution itself. When perturbation is activated, each individual is assigned a symmetric α-stable distribution whose stability index $\alpha_{i}^{g}$ is determined by its fitness rank. Consequently, higher-ranked individuals receive lighter-tailed perturbations, whereas lower-ranked individuals receive heavier-tailed perturbations.

### Positioning of this work

The proposed ALP operator complements both adaptive DE frameworks and previous Lévy-based variation mechanisms. Within L-SRTDE, the baseline mutation strategy, success-rate feedback, population management, and selection procedures remain unchanged. ALP acts specifically at the recombination level and separates two forms of perturbation control: the dimension-wise jumping probability $JR_{j}^{g}$ determines where and how frequently perturbations are activated, whereas the rank-conditioned stability index $\alpha_{i}^{g}$ determines how heavy-tailed the perturbation is for each individual. Thus, unlike approaches that adapt conventional DE parameters or introduce an additional Lévy-search stage, ALP provides individual-level tail-shape adaptation while preserving the underlying optimization framework. This modular design allows the contribution of rank-conditioned tail control to be evaluated independently within the strong L-SRTDE baseline.

## Proposed algorithm

This section introduces ALP, a perturbation operator for DE recombination that assigns distinct tail behaviors to individual solutions. The key idea is to separate two design decisions: where perturbations are applied (via dimension-wise activation, $JR_{j}^{g}$) and how disruptive they are (via individual-wise tail heaviness, $\alpha_{i}^{g}$). We first define the ALP operator and then describe how it integrates into the L-SRTDE algorithm [9].

### ALP

ADCP [8] concentrates perturbations on coordinates that exhibit strong contraction, using a dimension-specific jumping schedule $JR_{j}^{g}$. However, ADCP fixes the perturbation kernel to the Cauchy distribution, giving all individuals the same tail behavior. ALP generalizes this approach by maintaining the same dimension-wise activation while allowing the perturbation’s tail heaviness to vary across individuals based on their fitness rank. As summarized in Table 1, ALP differs from CP and ADCP by adapting not only the perturbation probability but also the perturbation tail behavior at the individual level. Thus, ALP extends dimension-wise activation with rank-conditioned α-stable tail control.

**Table 1:** Relation to ADCP and distinction from fixed-kernel perturbation.

Method	Perturbation probability	Perturbation distribution	Individual-level tail control
CP	Global or fixed	Cauchy	No
ADCP	Dimension-wise adaptive	Cauchy	No
ALP	Dimension-wise adaptive	Rank-conditioned α-stable	Yes

#### Symmetric α-stable perturbations.

Let $S_{\alpha }(\beta ,c,\mu )$ denote an α-stable distribution with stability α∈(0,2], skewness β∈[−1,1], scale *c* > 0, and location μ∈ℝ. In this study, we focus on the symmetric case β=0 and control exploration primarily through α. Notably, α=2 yields a Gaussian distribution, while α=1 recovers the (symmetric) Cauchy distribution.

#### Rank-to-α assignment.

At generation *g*, let $N^{g}$ be the current population size, and let ${\mathrm{rank}}^{g}(i)\in \{1,...,N^{g}\}$ denote the fitness rank of ${\vec{x}}_{i}^{\;g}$ within the population ${𝒫}^{g}$ (1: best, $N^{g}$: worst). ALP assigns the stability index using linear interpolation:


$$\begin{array}{c} \alpha_{i}^{\;g}=\alpha_{\max}-(\alpha_{\max}-\alpha_{\min})\frac{{\mathrm{rank}}^{\;g}(i)-1}{N^{g}-1}, \end{array}$$ (9)

where $0<\alpha_{\min}\leq \alpha_{\max}\leq 2$. This ensures that high-quality solutions (${\mathrm{rank}}^{g}(i)$ small) receive larger α (lighter tails), while lower-quality solutions receive smaller α values (heavier tails).

#### ALP recombination operator.

ALP modifies binomial crossover only when a coordinate would otherwise be copied from the target. Specifically, for a mutant vector ${\vec{v}}_{i}^{\;g}$ and target ${\vec{x}}_{i}^{\;g}$, the trial coordinate is computed as:


$$\begin{array}{c} u_{i,j}^{\;g}=\left\{ \begin{array}{cc} v_{i,j}^{\;g}, & \text{if }r_{i,j}^{\left(1\right)}\leq CR_{i}^{g}\text{ or }j=j_{rand},\\ rnds\;\left(\alpha_{i}^{g},\;0,\;c,\;x_{i,j}^{\;g}\right), & \text{if }r_{i,j}^{\left(1\right)}>CR_{i}^{g}\text{ and }r_{i,j}^{\left(2\right)}\leq JR_{j}^{\;g},\\ x_{i,j}^{\;g}, & \text{otherwise,} \end{array}\right. \end{array}$$ (10)

where $r_{i,j}^{\left(1\right)},r_{i,j}^{\left(2\right)}\sim U(0,1)$, $CR_{i}^{g}\in [0,1]$ is the crossover rate sampled for the individual *i*, and $JR_{j}^{\;g}\in [0,1]$ is the dimension-wise jumping probability derived from the convergence indicator in Eqs. (6)–(7). Here, rnds(α,β,c,μ) denotes a draw from $S_{\alpha }(\beta ,c,\mu )$. In our implementation, we fix the scale at *c* = 0.1.

#### Sampling (CMS method).

Since stable distributions generally lack closed-form densities, we generate α-stable samples using the Chambers–Mallows–Stuck (CMS) method [30–32]. For the symmetric case β=0, sampling simplifies as follows: let $V\sim U(-\frac{\pi }{2},\frac{\pi }{2})$ and W~Exp(1). A unit-scale, zero-location symmetric stable draw $X\sim S_{\alpha }(0,1,0)$ is generated by:


$$\begin{array}{c} X=\left\{ \begin{array}{cc} \frac{\sin(\alpha V)}{(\cos V{)}^{1/\alpha }}{\left(\frac{\cos\;\left((1-\alpha )V\right)}{W}\right)}^{\frac{1-\alpha }{\alpha }}, & \alpha \text{≠}1,\\ \tan V, & \alpha =1. \end{array}\right. \end{array}$$ (11)

Finally, Y=cX+μ yields $Y\sim S_{\alpha }(0,c,\mu )$, which is used in Eq. (10). When α=1 and $\mu =x_{i,j}^{\;g}$, Eq. (10), this reduces to a Cauchy-centered perturbation at the target coordinate.

### L-SRTDE with ALP

We denote the resulting algorithm as L-SRTDE-ALP. Rather than redefining the entire baseline, we highlight the minimal set of modifications:

- Fitness ranks are computed in ${𝒫}^{g}$ and $\alpha_{i}^{g}$ is assigned according to Eq. (9).
- Per-dimension convergence levels $CL_{j}^{g}$ and jumping probabilities $JR_{j}^{g}$ are calculated as in ADCP [8].
- The baseline crossover is replaced with the ALP recombination rule in Eq. (10).

All other components, including the dual-population structure, mutation strategy, success-rate feedback, and insertion-based selection, remain consistent with L-SRTDE [9]. For completeness, we summarize the elements that interact directly with ALP.

#### Initialization and population resizing.

The initial population is set as ${𝒫}^{0}=\{{\vec{x}}_{i}^{\;0}{\}}_{i=1}^{N_{\max}}$ uniformly sampled over Ω, and the top population ${𝒯}^{0}$ is initialized as a copy of ${𝒫}^{0}$. The initial coordinate-wise spreads $\{\sigma_{j}^{0}{\}}_{j=1}^{d}$ are recorded, and the crossover memory $\{M_{CR,h}{\}}_{h=1}^{H}$ is initialized following SHADE-style procedures.

During evaluations, L-SRTDE applies linear population size reduction. Given the maximum budget ${FE}_{\max}$ and current usage ${FE}_{cur}$, the allowed population size is calculated as:


$$\begin{array}{c} N_{allow}=\left\lceil N_{\min}+(N_{\max}-N_{\min})\frac{{FE}_{\max}-{FE}_{cur}}{{FE}_{\max}}\right\rceil . \end{array}$$ (12)

If $|{𝒫}^{g}|>N_{allow}$, the worst-performing individuals are removed.

The generation-wise success rate is defined as:


$$\begin{array}{c} SR^{g}=\frac{N_{s}^{g}}{N^{g}}, \end{array}$$ (13)

where $N_{s}^{g}$ is the number of successful insertions in the generation *g* and $N^{g}=|{𝒫}^{g}|$ after reduction.

#### Mutation and success-rate feedback (baseline).

For each target ${\vec{x}}_{i}^{\;g}\in {𝒫}^{g}$, L-SRTDE generates a mutant vector (r-new-to-ptop/n/t):


$$\begin{array}{c} {\vec{v}}_{i}^{\;g}={\vec{x}}_{r_{1}}^{\;g}+F_{i}^{\;g}\left({\vec{x}}_{pbest}^{\;top,g}-{\vec{x}}_{i}^{\;g}\right)+F_{i}^{\;g}\left({\vec{x}}_{r_{2}}^{\;g}-{\vec{x}}_{r_{3}}^{\;top,g}\right), \end{array}$$ (14)

where the indices are selected as in [9] and *pbest* is sampled from the top $100p_{b}^{g}\%$ of ${𝒯}^{g}$.

The mean scaling factor and elite fraction are updated based on the previous generation’s success rate:


$$\begin{array}{cc} m_{F}^{g} & =0.4+0.25\tanh\;\left(5\;SR^{g-1}\right), \end{array}$$ (15)

$$\begin{array}{cc} p_{b}^{g} & =0.7\exp\;\left(-7\;SR^{g-1}\right). \end{array}$$ (16)

Then $F_{i}^{g}$ is sampled around $m_{F}^{g}$ with the standard positivity handling as described in [9].

#### Crossover learning and ALP application.

Nominal crossover rates are sampled using SHADE-style memory:


$$\begin{array}{c} CR_{i}^{g}\sim 𝒩(M_{CR,h},0.05^{2}), \end{array}$$ (17)

with truncation to the interval [0,1].

At the start of the generation *g*, we first (i) compute ranks and assign $\alpha_{i}^{g}$ according to Eq. (9), (ii) compute $CL_{j}^{g}$ and $JR_{j}^{g}$ from Eqs. (6)–(7), and then (iii) generate ${\vec{u}}_{i}^{\;g}$ by Eq. (10). Following the effective-crossover concept, we record


$$\begin{array}{c} CR_{a,i}^{g}=\frac{1}{d}\sum_{j=1}^{d}𝕀\;\left(u_{i,j}^{\;g}=v_{i,j}^{\;g}\right), \end{array}$$ (18)

and successful $CR_{a,i}^{g}$ values are used to update the memory according to the standard SHADE procedure.

#### Selection and bookkeeping.

For each offspring, if $f({\vec{u}}_{i}^{\;g})\leq f({\vec{x}}_{r_{1}}^{\;g})$, the offspring is considered successful and inserted at the rotating position $n_{c}$:


$$\begin{array}{c} {\vec{x}}_{n_{c}}^{\;g+1}\leftarrow {\vec{u}}_{i}^{\;g}\;\text{if }f({\vec{u}}_{i}^{\;g})\leq f({\vec{x}}_{r_{1}}^{\;g}). \end{array}$$ (19)

At the end of the generation, we compute $SR^{g}$ by Eq. (13) and update $m_{F}^{g+1}$ and $p_{b}^{g+1}$. The procedure repeats until the evaluation budget is exhausted. Pseudocode is provided in Algorithms 1 and 2.

**Algorithm 1: L-SRTDE-ALP (L-SRTDE with Adaptive Lévy Perturbation)**

**Require:** *d*, ${FE}_{\max}$, $N_{\max}$, $N_{\min}$, f(·), $JR_{\min}$, $JR_{\max}$, κ, *c*, $\alpha_{\min}$, $\alpha_{\max}$

**Ensure:** ${\vec{x}}_{best}^{top}$, $f({\vec{x}}_{best}^{top})$

1: Initialize: g←0, ${FE}_{cur}\leftarrow 0$, $N\leftarrow N_{\max}$

2: Set $SR_{prev}\leftarrow 0.5$, H←5, $k_{p}\leftarrow 3$

3: Set crossover memory $M_{CR}[h]\leftarrow 1$ for all h=1,...,H

4: Set memory update pointer $h_{upd}\leftarrow 1$ and insertion pointer $n_{c}\leftarrow 1$

5: **Initialization:** For *i* = 1 to *N* sample ${\vec{x}}_{i}^{new}\sim U(\Omega )$; evaluate $f({\vec{x}}_{i}^{new})$; ${FE}_{cur}\leftarrow {FE}_{cur}+1$

6: Set ${\vec{x}}^{top}\leftarrow {\vec{x}}^{new}$ (copy) and compute initial spreads $\sigma_{j}^{0}$ for j=1,...,d

7: **while** ${FE}_{cur}<{FE}_{\max}$ **do**

8:  sort ${\vec{x}}^{new}$ by fitness to obtain ${\mathrm{rank}}^{g}(i)$ for all *i*

9:   compute $\sigma_{j}^{g}$ from ${\vec{x}}^{top}$; compute $CL_{j}^{g}$ (Eq. (6)) and $JR_{j}^{g}$ (Eq. (7)) for all *j*

10:  compute $\alpha_{i}^{g}$ for all *i* using Eq. (9)

11:  $m_{F}\leftarrow 0.4+0.25\tanh(5\;SR_{prev})$ (Eq. (15)), $p_{b}\leftarrow 0.7\exp(-7\;SR_{prev})$ (Eq. (16))

12:  Initialize success containers: $S_{CR}\leftarrow \emptyset$, $S_{\Delta f}\leftarrow \emptyset$, 𝒮←∅, $N_{s}\leftarrow 0$

13:  **for** *i* = 1 to *N* **do**

14:   $({\vec{u}}_{i},CR_{a,i},r_{1})\leftarrow$ **MakeTrial_ALP**$\left(i,{\vec{x}}^{new},{\vec{x}}^{top},{\mathrm{rank}}^{g},JR^{g},\alpha_{i}^{g},m_{F},p_{b},M_{CR},k_{p},c\right)$

15:   Repair ${\vec{u}}_{i}$ (bound handling); evaluate $f({\vec{u}}_{i})$; ${FE}_{cur}\leftarrow {FE}_{cur}+1$

16:   **if** $f({\vec{u}}_{i})\leq f({\vec{x}}_{r_{1}}^{new})$ **then**

17:    $S_{CR}\leftarrow S_{CR}\cup \{CR_{a,i}\}$

18:    $S_{\Delta f}\leftarrow S_{\Delta f}\cup \{f({\vec{x}}_{r_{1}}^{new})-f({\vec{u}}_{i})\}$

19:    ${\vec{x}}_{n_{c}}^{new}\leftarrow {\vec{u}}_{i}$; update its fitness value

20:    $𝒮\leftarrow 𝒮\cup \{{\vec{u}}_{i}\}$; $N_{s}\leftarrow N_{s}+1$

21:    $n_{c}\leftarrow 1+(n_{c}\mathrm{mod}N)$

22:   **end if**

23:   **if** ${FE}_{cur}\geq {FE}_{\max}$ **then**

24:    **break**

25:   **end if**

26:  **end for**

27:  $SR_{prev}\leftarrow N_{s}/N$

28:  $N_{next}\leftarrow \left\lceil N_{\min}+(N_{\max}-N_{\min})\frac{{FE}_{\max}-{FE}_{cur}}{{FE}_{\max}}\right\rceil$ (Eq. (12))

29:  ${\vec{x}}^{top}\leftarrow$ best $N_{next}$ individuals of $\left({\vec{x}}^{top}\cup 𝒮\right)$

30:  if $N>N_{next}$, remove worst individuals from ${\vec{x}}^{new}$ until size $N_{next}$

31:  $N\leftarrow N_{next}$; $n_{c}\leftarrow 1+((n_{c}-1)\mathrm{mod}N)$

32:  **if** $S_{CR}\text{≠}\emptyset$ **then**

33:   Update $M_{CR}[h_{upd}]$ using $S_{CR}$ and $S_{\Delta f}$ (SHADE update rule); $h_{upd}\leftarrow 1+(h_{upd}\mathrm{mod}H)$

34:  **end if**

35:  g←g+1

36: **end while**

37: **return** best individual in ${\vec{x}}^{top}$

**Algorithm 2. MakeTrial_ALP: Mutation + ALP Crossover**

**Require:** *i*, ${\vec{x}}^{new}$, ${\vec{x}}^{top}$, ${\mathrm{rank}}^{g}$, $JR^{g}=\{JR_{j}^{g}{\}}_{j=1}^{d}$, $\alpha_{i}^{g}$, $m_{F}$, $p_{b}$, $M_{CR}$, $k_{p}$, *c*

**Ensure:** ${\vec{u}}_{i}$, $CR_{a,i}$, *r*_1_

1: sample $F_{i}\sim 𝒩(m_{F},0.02^{2})$ until $F_{i}\in (0,1)$

2: Choose memory slot h←randi[1,H]

3: Sample crossover rate $CR_{i}\leftarrow clip(randn(M_{CR}[h],0.05),0,1)$

4: $r_{1}\leftarrow randi(1,N)$; $p_{\max}\leftarrow \max(1,\lfloor N\;p_{b}\rfloor )$; $pbest\leftarrow randi(1,p_{\max})$ {${\vec{x}}^{top}$ assumed sorted best-first}

5: **repeat**

6:  $r_{2}\leftarrow$ rank-based selection from ${\vec{x}}^{new}$ with pressure $k_{p}$ using ${\mathrm{rank}}^{g}$

7:  $r_{3}\leftarrow randi(1,N)$

8: **until** indices $r_{1},r_{2},r_{3},pbest$ are all distinct and different from *i*

9: **Mutation (r-new-to-ptop/n/t):**

10: ${\vec{v}}_{i}\leftarrow {\vec{x}}_{r_{1}}^{new}+F_{i}({\vec{x}}_{pbest}^{top}-{\vec{x}}_{i}^{new})+F_{i}({\vec{x}}_{r_{2}}^{new}-{\vec{x}}_{r_{3}}^{top})$ (Eq. (14))

11: **ALP crossover:** $j_{rand}\leftarrow randi(1,d)$; count←0

12: **for** *j*=1 to *d* **do**

13:  Draw $r^{\left(1\right)}\sim U(0,1)$ and $r^{\left(2\right)}\sim U(0,1)$

14:  **if** $r^{\left(1\right)}\leq CR_{i}$ **or** $j=j_{rand}$ **then**

15:   $u_{i,j}\leftarrow v_{i,j}$; count←count+1

16:  **else if** $r^{\left(2\right)}\leq JR_{j}^{g}$ **then**

17:   $u_{i,j}\leftarrow rnds(\alpha_{i}^{g},0,c,x_{i,j}^{new})$ {ALP (Eq. (10))}

18:  **else**

19:   $u_{i,j}\leftarrow x_{i,j}^{new}$

20:  **end if**

21: **end for**

22: $CR_{a,i}\leftarrow count/d$ (Eq. (18))

23: **return** ${\vec{u}}_{i}$, $CR_{a,i}$, *r*_1_

## Experimental study

### Experimental setup

We evaluate the proposed L-SRTDE-ALP on the IEEE CEC 2017 single-objective, bound-constrained benchmark suite [10], following the standard evaluation protocol. The suite comprises 29 functions: 2 unimodal functions, 7 simple multimodal functions, 10 hybrid functions, and 10 composition functions. Although the suite was originally introduced for the 2017 Special Session and Competition on single-objective bound-constrained real-parameter numerical optimization, it remains an active reference benchmark in recent CEC optimization research. In particular, the CEC 2024 competition has been described as being based on the CEC 2017 benchmark suite, and the IEEE CEC 2026 single-objective bound-constrained competition adopts the CEC 2017 suite at *D* = 30. Therefore, evaluating the proposed method on CEC 2017 is not only historically comparable but also relevant to recent competition-level algorithm assessment.

#### Compared algorithms.

To contextualize L-SRTDE-ALP, we benchmark it against 11 benchmark algorithms, including recent DE variants and three leading CEC 2017 methods: L-SRTDE [9], BWDE [33], DPSDE [34], FODE [35], MSDE [36], RDE [37], RSDE [38], SMLDE [39], EBOwithCMAR [40], jSO [20], and LSHADE-SPACMA [41]. Among these, L-SRTDE was the winner of the CEC 2024 competition, whereas EBOwithCMAR, jSO, and LSHADE-SPACMA were among the leading algorithms in the CEC 2017 competition. The remaining methods serve as additional state-of-the-art reference points, many of which incorporate Cauchy-based perturbation operators. For each comparator, we use the parameter settings recommended in its original publication. In constructing L-SRTDE-ALP, all parameters and fixed numerical constants of the underlying L-SRTDE framework are retained without modification. Similarly, the parameters governing dimension-wise perturbation activation and the perturbation scale, namely ($JR_{\min}=0.1$), ($JR_{\max}=0.2$), (κ=10), and (*c* = 0.1), are inherited unchanged from ADCP. These inherited settings are fixed a priori and are not retuned for L-SRTDE-ALP, allowing the effect of rank-conditioned tail-shape adaptation to be isolated. Consequently, the only additional parameters introduced by ALP are the stability-index bounds ($\alpha_{\min}$) and ($\alpha_{\max}$). We set ($\alpha_{\min}=0.3$) and ($\alpha_{\max}=1.7$), and examine the suitability and sensitivity of these values in the Analysis of Parameter Settings subsection. Table 2 summarizes the parameter configurations used in all experiments.

**Table 2:** Parameter configurations for all algorithms.

Algorithm	Year	Parameter Settings
L-SRTDE-ALP	—	L-SRTDE settings + $JR_{\min}=0.1,JR_{\max}=0.2,\kappa =10,c=0.1,\alpha_{\min}=0.3,\alpha_{\max}=1.7$
L-SRTDE	2024	$N_{init}=20D,N_{\min}=4,H=5,k_{p}=3,M_{CR}^{\left(0\right)}=1,SR^{\left(0\right)}=0.5$
BWDE	2024	$N_{init}=18D,N_{\min}=4,k=3,p=0.11,P_{j}=0.2,q=0.3,u=0.5$
DPSDE	2024	$N_{init}=18D,N_{\min}=4,k=30,\Delta =0.3$
FODE	2025	$N_{init}=18D,N_{\min}=4,k=3,p=0.11,P_{j}=0.2,\alpha =0.995,r=6$
MSDE	2024	$N_{init}=18D,N_{\min}=4,H=5,k=3,p=0.11,P_{j}=0.2,NA=1.0NP,\theta_{1}=10,\theta_{2}=25$
RDE	2024	$N_{\max}=18D,N_{\min}=4,H=5,k_{r}=3,p_{\max}=0.25,A_{r}=1,p_{r}=0.2,\mu_{F}^{\left(0\right)}=0.3,\mu_{CR}^{\left(0\right)}=0.8,\gamma_{1}^{\left(0\right)}=\gamma_{2}^{\left(0\right)}=0.5$
RSDE	2024	$N_{init}=18D,N_{\min}=4,k=3,p=0.11,P_{j}=0.2,\eta =0.2,\gamma =0.15,\kappa =0.21$
SMLDE	2024	$N_{init}=75D^{\frac{2}{3}},N_{\min}=4,k=3,p=0.11,P_{j}=0.2,\zeta =0.5$
EBOwithCMAR	2017	$PS_{1,\max}=18D,PS_{1,\min}=4,PS_{2,\max}=46.8D,PS_{2,\min}=10,PS_{3}=4+3\log(D),H=6,\sigma =0.3,$
		$CS=200\;(30D),\;300\;(50D/100D),prob_{ls}=0.1,cfe_{ls}=0.25FE_{\max}$
jSO	2017	$N_{init}=25\log(D)\sqrt{D},N_{\min}=4,H=5,p_{\max}=0.25,p_{\min}=0.125,M_{F}^{\left(0\right)}=0.3,M_{CR}^{\left(0\right)}=0.8$
LSHADE-SPACMA	2017	$N_{init}=18D,N_{\min}=4,P_{best}=0.11,H=5,Arc_{rate}=1.4,F_{CP}=0.5,c=0.8$

#### Budget and performance metrics.

For each function and dimensionality, we perform 51 independent runs. The evaluation budget is set to ${FE}_{\max}=10000\cdot D$, where *D* denotes the problem dimension. Performance is reported as the mean error with its corresponding standard deviation over the 51 runs; values smaller than 10^−8^ are recorded as zero. In the result tables, the best mean error among all compared algorithms is highlighted in bold.

#### Statistical analysis.

Pairwise comparisons are conducted using the Wilcoxon rank-sum test [42]. To assess overall algorithm performance and establish a global ranking, we employ the Friedman test [43]. When appropriate, we also apply post hoc multiple-comparison procedures, including Bonferroni–Dunn [44], Holm [45], and Hochberg [46] corrections.

#### Implementation and hardware.

All methods are implemented in C++ and compiled using GCC. Experiments are run on Ubuntu 20.04.5 LTS with an AMD Ryzen Threadripper 2990WX CPU and 64 GB of RAM.

#### Random-number generation.

All stochastic algorithms used the C++ std::mt19937 pseudo-random number generator. Following the time-based seeding convention specified in the CEC 2017 experimental protocol, a global seed s was initialized as unsigned(time(NULL)) for each independent run. Four separate Mersenne Twister instances were then initialized using s, s + 100, s + 200, and s + 300 for the different random-variable generators used by the algorithms. The same PRNG and seed-generation procedure were applied to all compared algorithms.

### Comparison with recent algorithms and CEC 2017 competition winners

We evaluate L-SRTDE-ALP against 11 baselines across all 29 CEC 2017 functions. Complete function-level results, including the best, median, mean, and standard deviation over 51 independent runs, are provided in the Supporting Information. Empirical cumulative distribution functions (ECDFs) of the final objective errors are also reported to characterize the full distribution of the run-level outcomes. For each dimensionality, we summarize (i) pairwise outcomes from the Wilcoxon rank-sum test [42] and (ii) global rankings obtained using the Friedman test [43]. Post hoc comparisons with Bonferroni–Dunn, Holm, and Hochberg adjustments are subsequently reported.

#### 30-dimensional problems.

**Fig 1:**
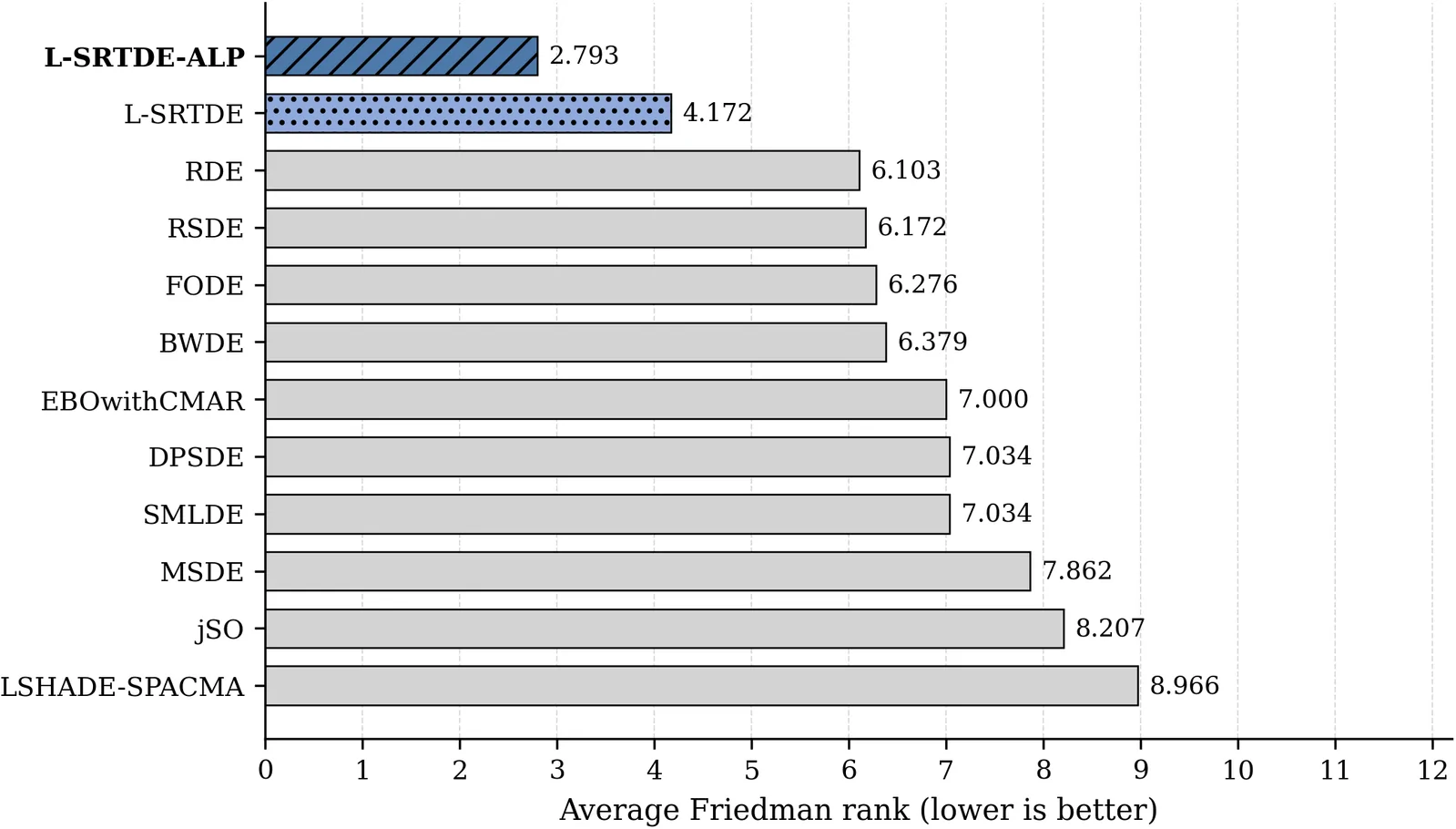
Algorithm rankings derived from Friedman test on 30-dimensional problems.

The Wilcoxon summaries in Table 3 indicate that L-SRTDE-ALP achieves a positive win–loss balance against most competitors. Specifically, against the four competition-level baselines, the win/loss/tie counts are 10/1/18 (L-SRTDE), 18/2/9 (EBOwithCMAR), 23/1/5 (jSO), and 22/2/5 (LSHADE-SPACMA). For the remaining seven methods, L-SRTDE-ALP records between 20 and 23 statistically significant wins, with only 2–3 losses. The Friedman ranking in Fig 1 assigns the lowest (best) average rank to L-SRTDE-ALP (2.793). Post-hoc results in Table 4 indicate that L-SRTDE-ALP has a lower average rank than every comparator, and the rank differences remain statistically significant after all three multiplicity corrections except in the comparison with L-SRTDE. For L-SRTDE, the unadjusted *p*-value is 0.145, and none of the multiplicity-adjusted tests identifies a statistically significant difference.

**Table 3:** Wilcoxon rank-sum results vs. L-SRTDE-ALP on 30-dimensional problems.

L-SRTDE-ALP vs.	+	–	≈
L-SRTDE	10	1	18
BWDE	22	2	5
DPSDE	23	2	4
FODE	23	2	4
MSDE	20	3	6
RDE	23	2	4
RSDE	22	2	5
SMLDE	23	2	4
EBOwithCMAR	18	2	9
jSO	23	1	5
LSHADE-SPACMA	22	2	5

**Table 4:** Post-Hoc p-values for compared algorithms on 30-dimensional problems.

L-SRTDE-ALP vs.	*z*	*p*	Bonf.	Holm	Hoch.
L-SRTDE	1.457	0.145	1.000	0.145	0.145
BWDE	3.788	**<0.001**	**0.002**	**0.001**	**0.001**
DPSDE	4.479	**<0.001**	**<0.001**	**<0.001**	**<0.001**
FODE	3.678	**<0.001**	**0.003**	**0.001**	**0.001**
MSDE	5.353	**<0.001**	**<0.001**	**<0.001**	**<0.001**
RDE	3.496	**<0.001**	**0.005**	**0.001**	**0.001**
RSDE	3.569	**<0.001**	**0.004**	**0.001**	**0.001**
SMLDE	4.479	**<0.001**	**<0.001**	**<0.001**	**<0.001**
EBOwithCMAR	4.443	**<0.001**	**<0.001**	**<0.001**	**<0.001**
jSO	5.718	**<0.001**	**<0.001**	**<0.001**	**<0.001**
LSHADE-SPACMA	6.519	**<0.001**	**<0.001**	**<0.001**	**<0.001**

#### 50-dimensional problems.

**Fig 2:**
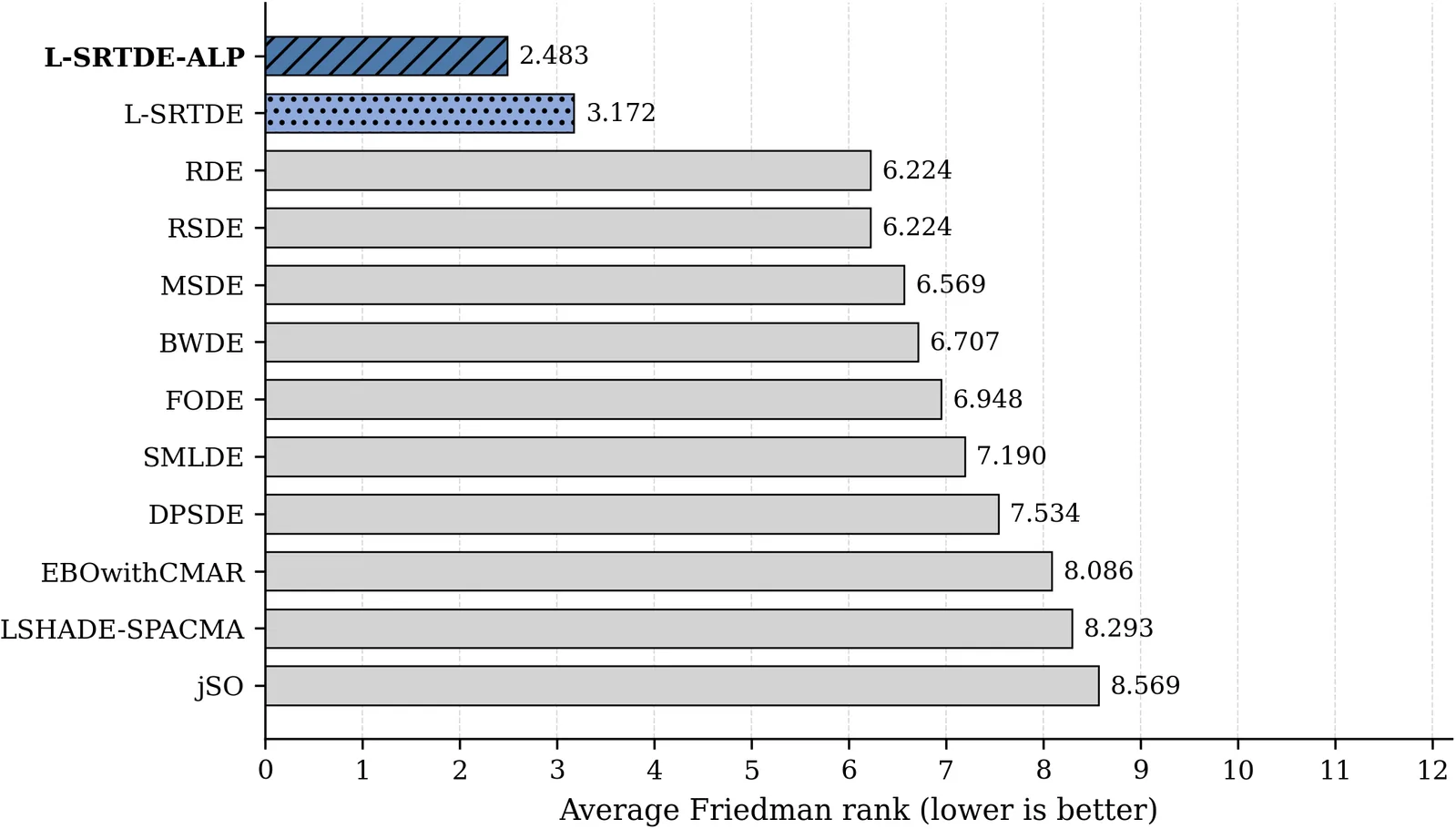
Algorithm rankings derived from Friedman test on 50-dimensional problems.

At *D* = 50, L-SRTDE-ALP retains the lowest overall Friedman rank. As shown in Table 5, it yields 10/4/15 versus L-SRTDE and achieves dominant outcomes against EBOwithCMAR (22/3/4), jSO (24/2/3), and LSHADE-SPACMA (21/3/5). Across the other seven baselines, L-SRTDE-ALP achieves 24 statistically significant wins and 2 losses against each of the other seven recent baselines. The Friedman test again ranks L-SRTDE-ALP first overall, with an average rank of 2.483, as shown in Fig 2. In Table 6, post-hoc comparisons confirm statistical significance against all competitors except L-SRTDE (e.g., *p* = 0.466).

**Table 5:** Wilcoxon rank-sum results vs. L-SRTDE-ALP on 50-dimensional problems.

L-SRTDE-ALP vs.	+	–	≈
L-SRTDE	10	4	15
BWDE	24	2	3
DPSDE	24	2	3
FODE	24	2	3
MSDE	24	2	3
RDE	24	2	3
RSDE	24	2	3
SMLDE	24	2	3
EBOwithCMAR	22	3	4
jSO	24	2	3
LSHADE-SPACMA	21	3	5

**Table 6:** Post-hoc p-values for compared algorithms on 50-dimensional problems.

L-SRTDE-ALP vs.	*z*	*p*	Bonf.	Holm	Hoch.
L-SRTDE	0.728	0.466	1.000	0.466	0.466
BWDE	4.461	**<0.001**	**<0.001**	**<0.001**	**<0.001**
DPSDE	5.335	**<0.001**	**<0.001**	**<0.001**	**<0.001**
FODE	4.716	**<0.001**	**<0.001**	**<0.001**	**<0.001**
MSDE	4.316	**<0.001**	**<0.001**	**<0.001**	**<0.001**
RDE	3.951	**<0.001**	**0.001**	**<0.001**	**<0.001**
RSDE	3.951	**<0.001**	**0.001**	**<0.001**	**<0.001**
SMLDE	4.971	**<0.001**	**<0.001**	**<0.001**	**<0.001**
EBOwithCMAR	5.918	**<0.001**	**<0.001**	**<0.001**	**<0.001**
jSO	6.428	**<0.001**	**<0.001**	**<0.001**	**<0.001**
LSHADE-SPACMA	6.136	**<0.001**	**<0.001**	**<0.001**	**<0.001**

#### 100-dimensional problems.

**Fig 3:**
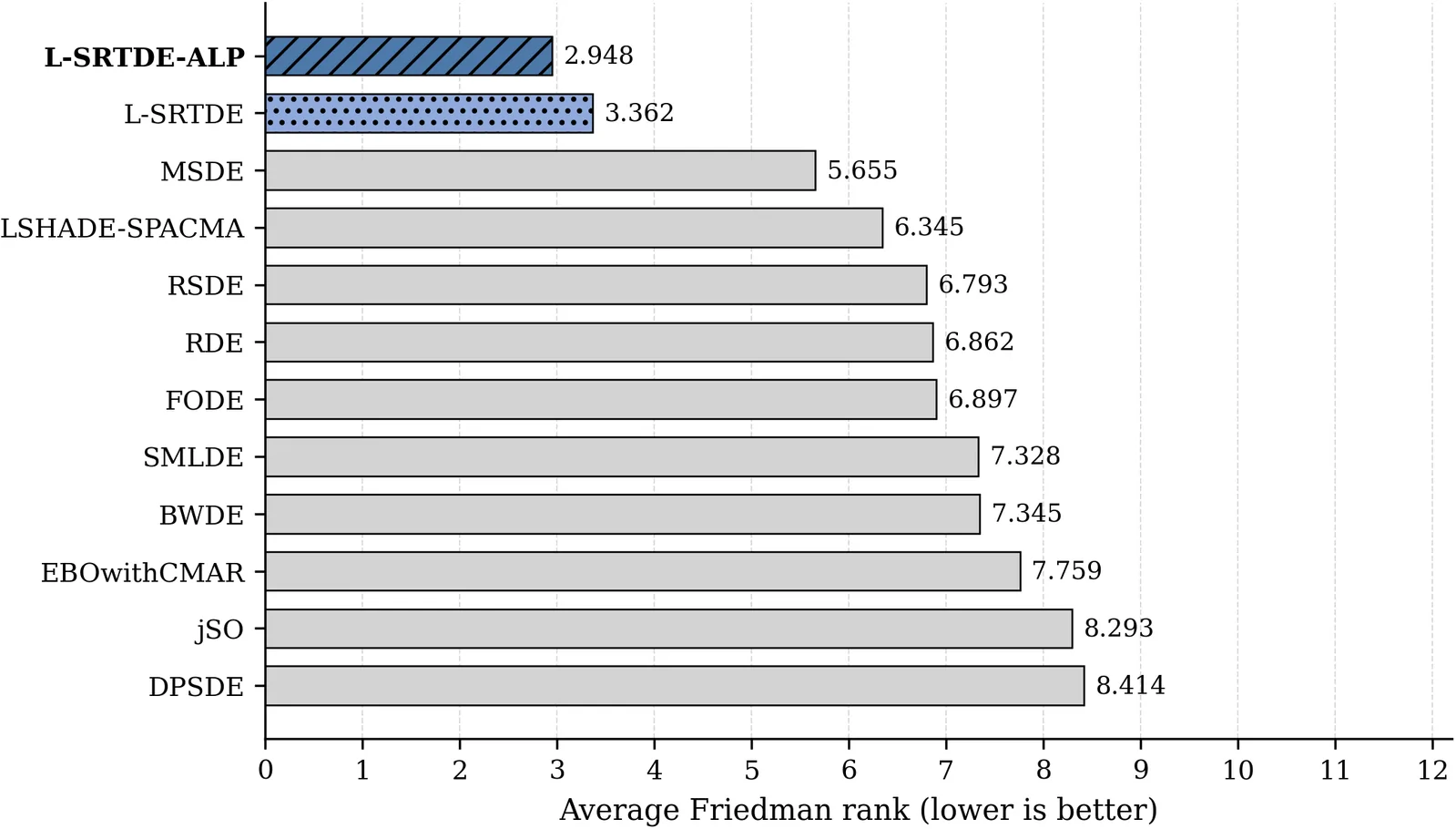
Algorithm rankings derived from Friedman test on 100-dimensional problems.

For the most challenging setting (*D* = 100), Table 7 shows that L-SRTDE-ALP maintains strong robustness against most algorithms, including 10/5/14 versus L-SRTDE, 25/3/1 versus EBOwithCMAR, 24/4/1 versus jSO, and 22/6/1 versus LSHADE-SPACMA. Against the remaining seven baselines, L-SRTDE-ALP records 23–25 statistically significant wins and only 2–3 losses. The Friedman test assigns L-SRTDE-ALP the best mean rank (2.948) in Fig 3. Post-hoc analysis in Table 8 shows statistically significant differences against all methods except L-SRTDE (e.g., *p* = 0.662).

**Table 7:** Wilcoxon rank-sum results vs. L-SRTDE-ALP on 100-dimensional problems.

L-SRTDE-ALP vs.	+	–	≈
L-SRTDE	10	5	14
BWDE	24	3	2
DPSDE	24	3	2
FODE	25	3	1
MSDE	23	2	4
RDE	24	3	2
RSDE	24	3	2
SMLDE	25	3	1
EBOwithCMAR	25	3	1
jSO	24	4	1
LSHADE-SPACMA	22	6	1

**Table 8:** Post-hoc p-values for compared algorithms on 100-dimensional problems.

L-SRTDE-ALP vs.	*z*	*p*	Bonf.	Holm	Hoch.
L-SRTDE	0.437	0.662	1.000	0.662	0.662
BWDE	4.643	**<0.001**	**<0.001**	**<0.001**	**<0.001**
DPSDE	5.772	**<0.001**	**<0.001**	**<0.001**	**<0.001**
FODE	4.170	**<0.001**	**<0.001**	**<0.001**	**<0.001**
MSDE	2.859	**0.004**	**0.047**	**0.009**	**0.009**
RDE	4.133	**<0.001**	**<0.001**	**<0.001**	**<0.001**
RSDE	4.061	**<0.001**	**0.001**	**<0.001**	**<0.001**
SMLDE	4.625	**<0.001**	**<0.001**	**<0.001**	**<0.001**
EBOwithCMAR	5.080	**<0.001**	**<0.001**	**<0.001**	**<0.001**
jSO	5.645	**<0.001**	**<0.001**	**<0.001**	**<0.001**
LSHADE-SPACMA	3.587	**<0.001**	**0.004**	**0.001**	**0.001**

#### Summary.

Across all three dimensionalities, L-SRTDE-ALP obtains the lowest Friedman average rank and a positive Wilcoxon win–loss balance against every comparator. The post hoc analyses identify statistically significant rank differences between L-SRTDE-ALP and all competing methods except the direct baseline, L-SRTDE. These findings demonstrate that the proposed method is highly competitive with both recent optimizers and the leading CEC 2017 methods under the tested benchmark conditions. At the same time, the absence of a significant post hoc difference from L-SRTDE indicates that ALP should be interpreted as a conservative refinement of the underlying framework rather than as a uniformly superior redesign.

#### Convergence behavior.

**Fig 4:**
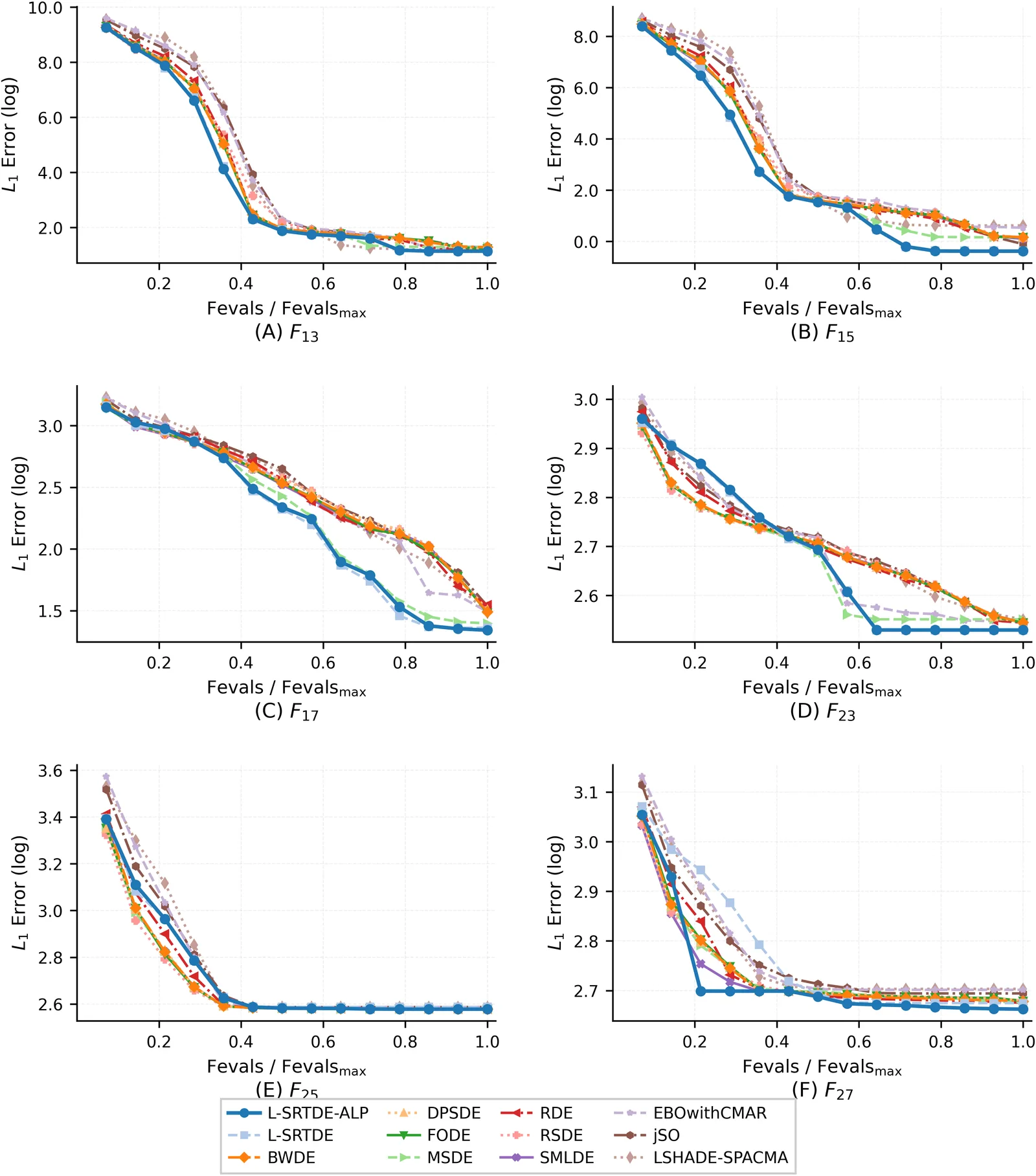
Median convergence curves on six 30-dimensional CEC 2017 functions. Curves show the median best-so-far objective error over 51 independent runs.

**Fig 5:**
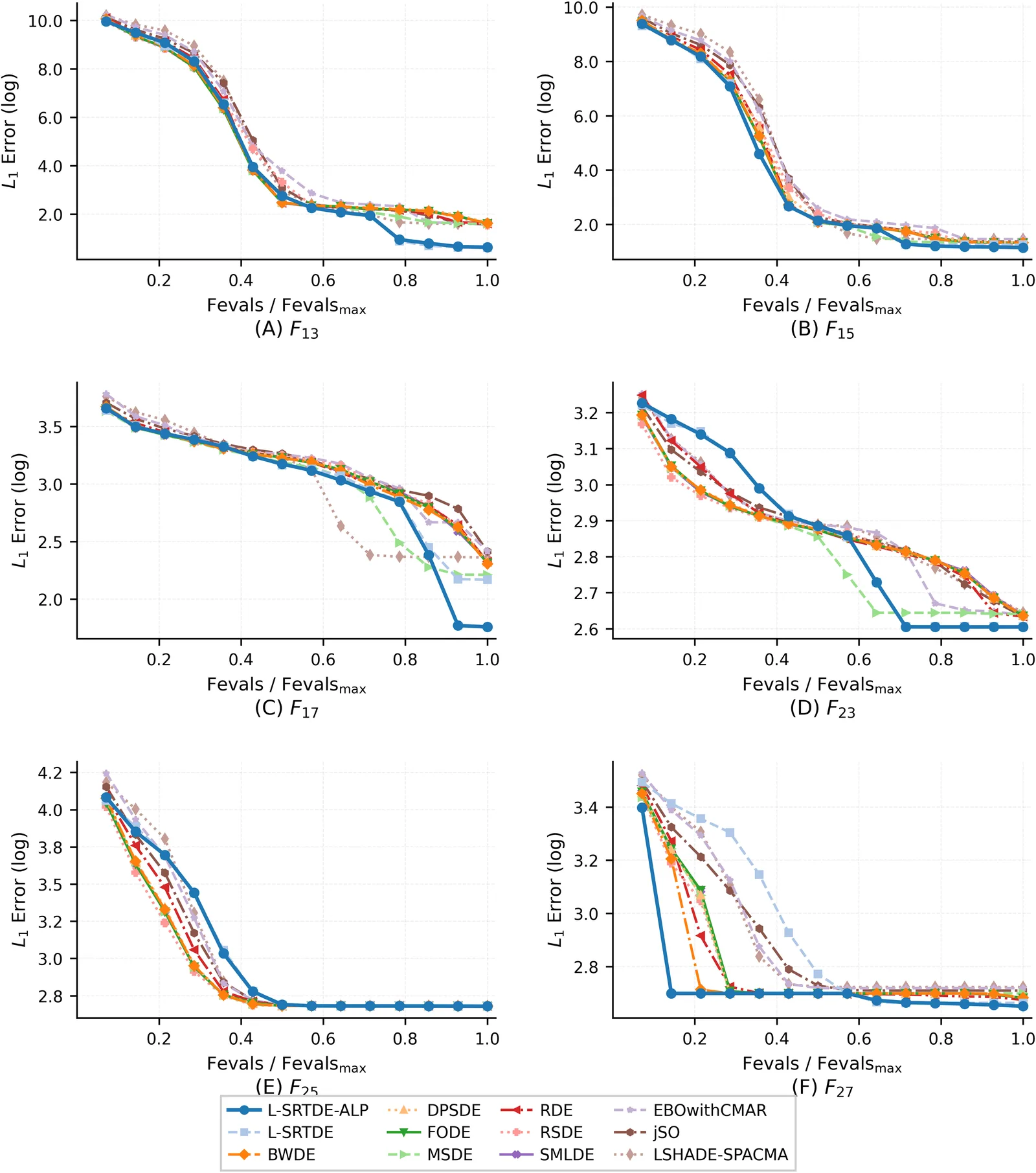
Median convergence curves on six 50-dimensional CEC 2017 functions. Curves show the median best-so-far objective error over 51 independent runs.

**Fig 6:**
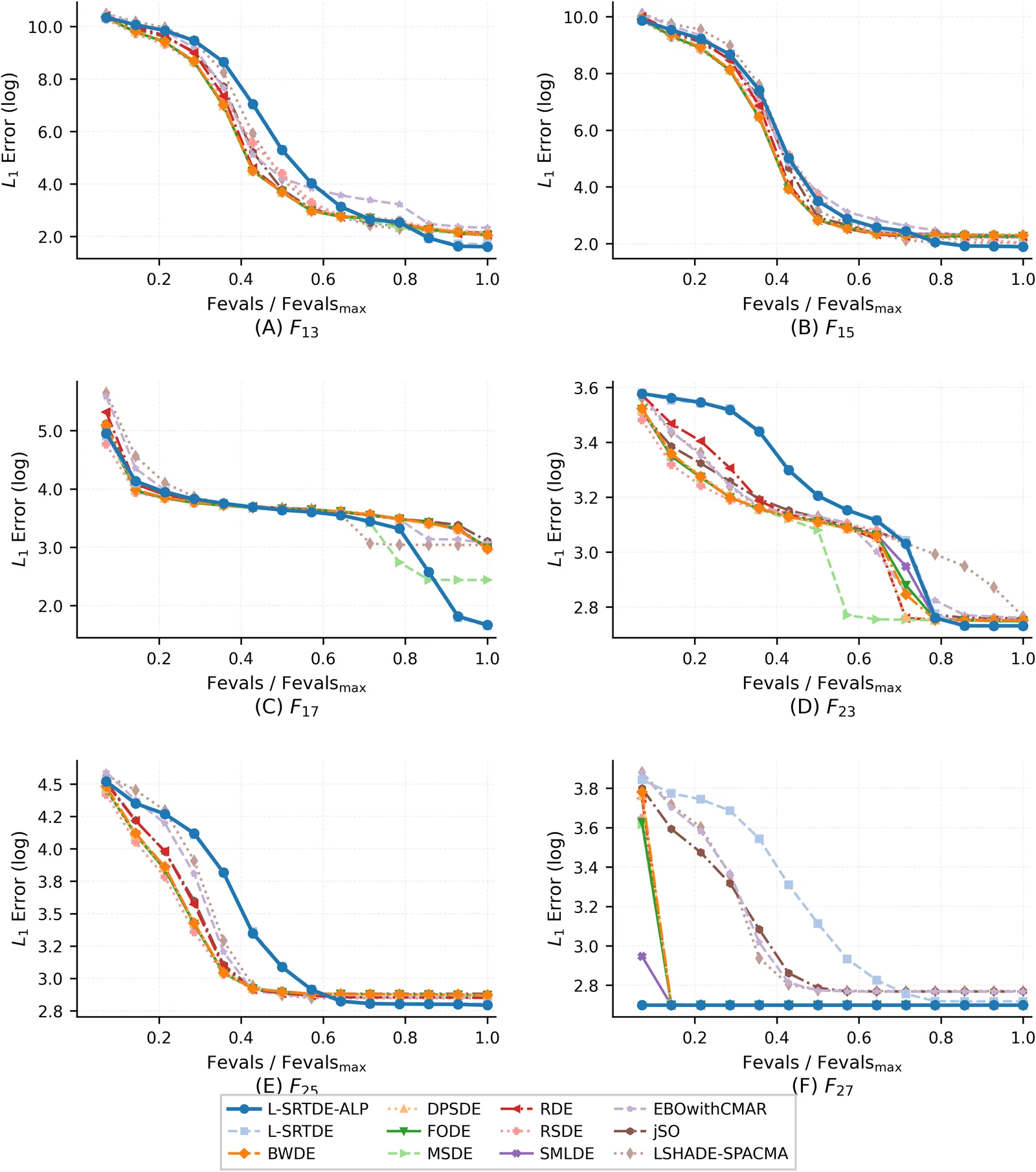
Median convergence curves on six 100-dimensional CEC 2017 functions. Curves show the median best-so-far objective error over 51 independent runs.

Figs 4, 5, 6 show the median optimization error trajectories for L-SRTDE-ALP and the 11 baselines on six representative CEC 2017 problems (*F*_13_, *F*_15_, *F*_17_, *F*_23_, *F*_25_, and *F*_27_). In most cases, L-SRTDE-ALP achieves a lower final error at termination, indicating more effective progress within the fixed evaluation budget.

#### Distribution of final errors.

**Fig 7:**
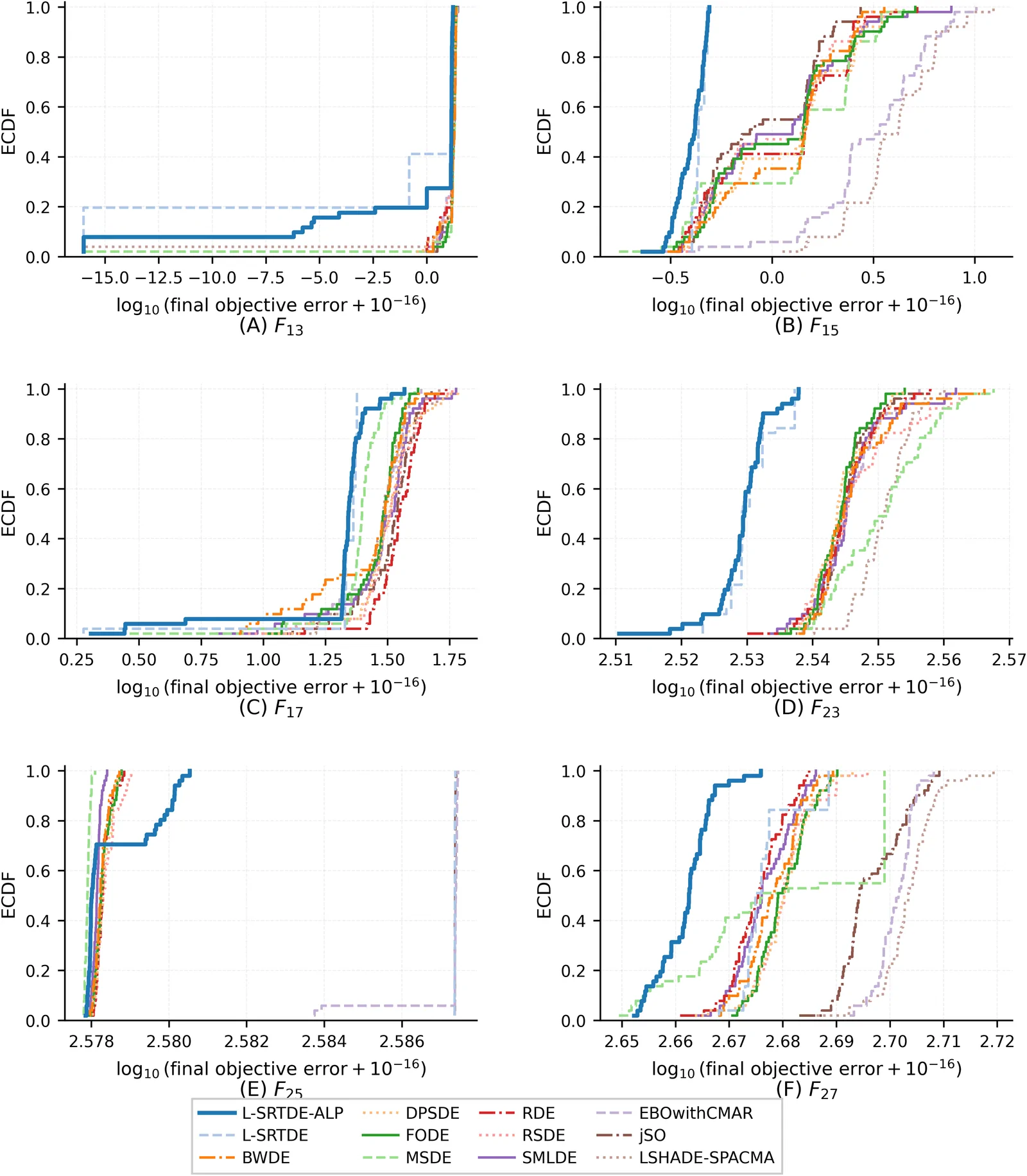
ECDFs of the final objective errors obtained by L-SRTDE-ALP and eleven competing algorithms on six 30-dimensional CEC 2017 functions over 51 independent runs.

**Fig 8:**
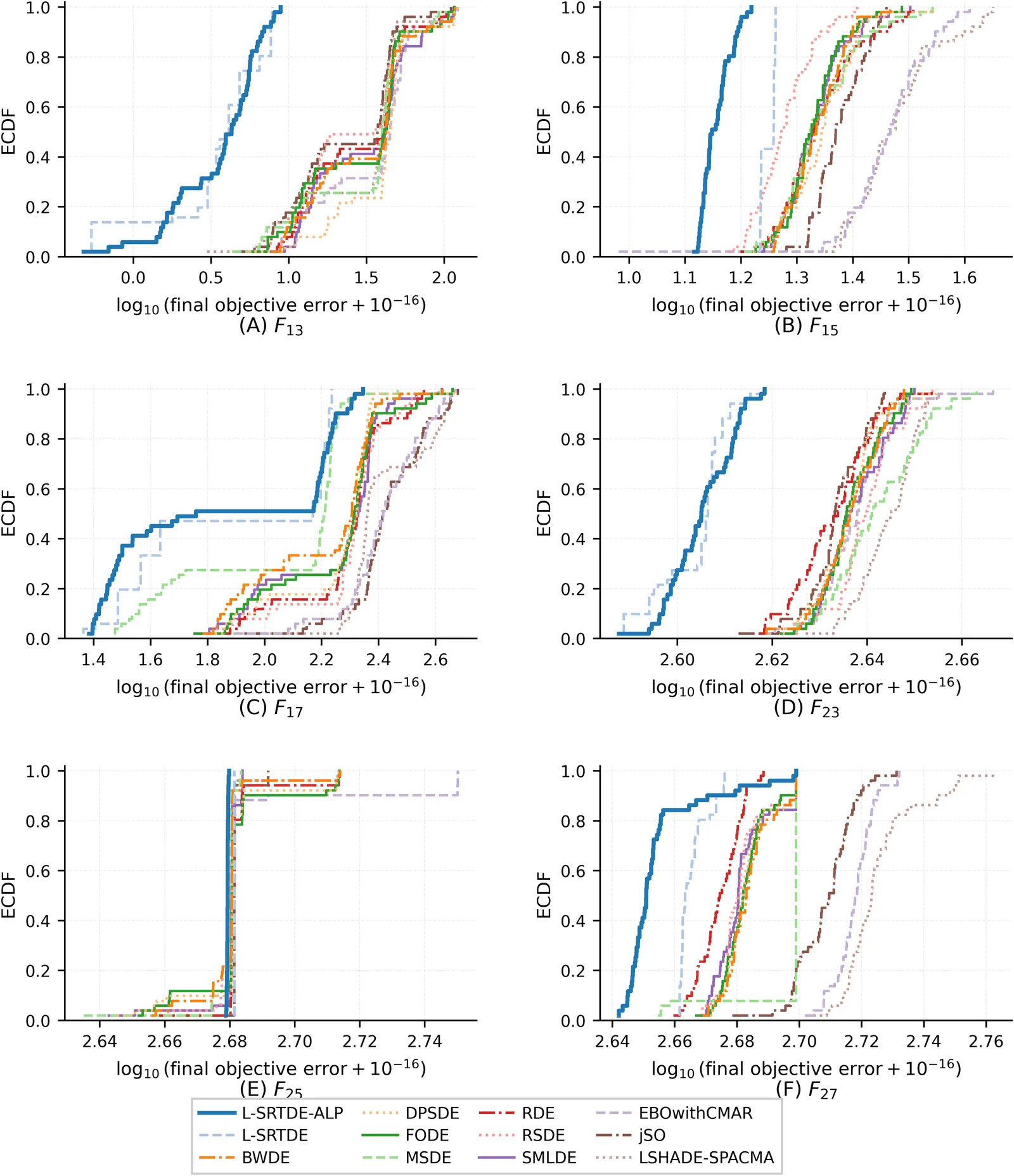
ECDFs of the final objective errors obtained by L-SRTDE-ALP and eleven competing algorithms on six 50-dimensional CEC 2017 functions over 51 independent runs.

**Fig 9:**
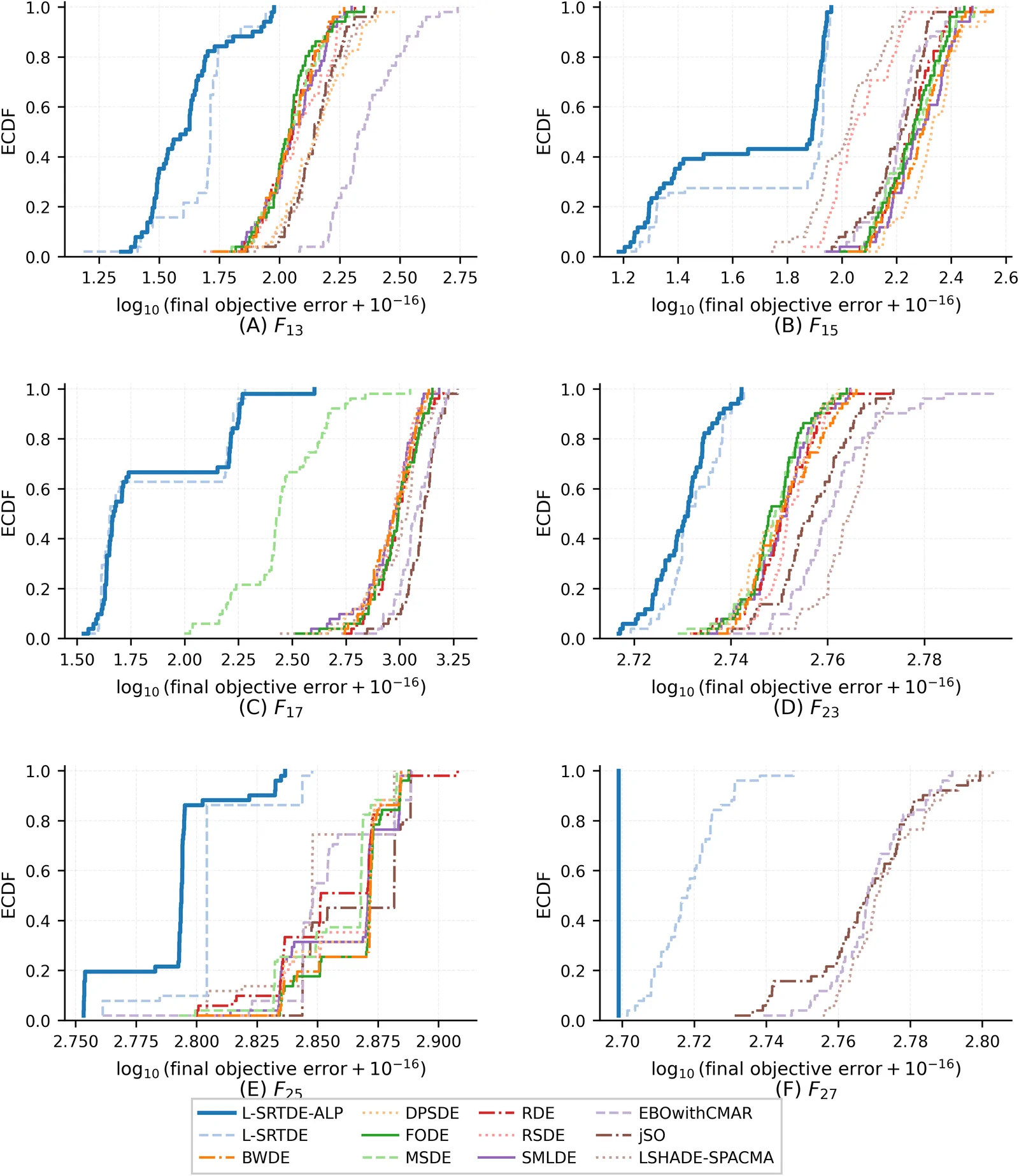
ECDFs of the final objective errors obtained by L-SRTDE-ALP and eleven competing algorithms on six 100-dimensional CEC 2017 functions over 51 independent runs.

Figs 7, 8, 9 show the empirical cumulative distribution functions (ECDFs) for L-SRTDE-ALP and the 11 baselines on six representative CEC 2017 problems (*F*_13_, *F*_15_, *F*_17_, *F*_23_, *F*_25_, and *F*_27_). In many of the CEC 2017 problems, the curve of L-SRTDE-ALP is shifted toward the upper-left relative to those of the competing methods, indicating a higher probability of attaining lower final errors. Overall, the ECDF results are consistent with the mean- and median-based comparisons and suggest that the favorable performance of L-SRTDE-ALP is generally reflected across the distribution of independent runs rather than being driven only by a small number of exceptionally successful trials.

### Comparison with cauchy perturbation variants

This experiment aims to isolate the effect of the perturbation operation on performance. To that end, we keep the underlying L-SRTDE pipeline unchanged and vary only the crossover perturbation component. We evaluate three within-framework variants: (i) L-SRTDE-CP, which employs a conventional (fixed-shape) Cauchy perturbation, (ii) L-SRTDE-ADCP, which incorporates dimension-wise jumping probabilities but still draws from a Cauchy distribution, and (iii) L-SRTDE-ALP (proposed), which follows the same dimension-wise jumping schedule while adapting the perturbation shape via a fitness-rank-controlled symmetric α-stable draw.

For each dimensionality (D∈{30,50,100}), we conducted pairwise comparisons across all 29 CEC 2017 functions using the Wilcoxon rank-sum test over 51 independent runs per function. Detailed per-function statistics are provided in the supplementary material.

#### Results at *D* = 30.

Table 9 shows that L-SRTDE-ALP achieves a slight net advantage over L-SRTDE-ADCP, with 9/7/13 wins/losses/ties. Against L-SRTDE-CP, the outcome is 4/0/25, indicating that ALP improves performance on a subset of functions while remaining statistically indistinguishable from the baseline on most problems at this dimensionality.

**Table 9:** Wilcoxon rank-sum test results comparing L-SRTDE-ALP against L-SRTDE-ADCP and L-SRTDE-CP on 30-dimensional problems.

L-SRTDE-ALP vs.	+	–	≈
L-SRTDE-ADCP	9	7	13
L-SRTDE-CP	4	0	25

#### Results at *D* = 50.

The differences become more pronounced (Table 10). L-SRTDE-ALP records 11/5/13 versus L-SRTDE-ADCP and 7/1/21 versus L-SRTDE-CP, showing a clearer positive win-loss balance in both comparisons. This indicates that introducing rank-guided control of tail heaviness is particularly beneficial in this mid-dimensional regime under the CEC evaluation budget.

**Table 10:** Wilcoxon rank-sum test results comparing L-SRTDE-ALP against L-SRTDE-ADCP and L-SRTDE-CP on 50-dimensional problems.

L-SRTDE-ALP vs.	+	–	≈
L-SRTDE-ADCP	11	5	13
L-SRTDE-CP	7	1	21

#### Results at *D* = 100.

For *D* = 100 (Table 11), the three variants often exhibit statistically similar performance, as reflected by the high number of ties. Even so, L-SRTDE-ALP maintains a positive net outcome: 8/2/19 against L-SRTDE-ADCP and 7/4/18 against L-SRTDE-CP. Thus, while gains narrow in this high-dimensional setting, ALP remains competitive and does not introduce systematic regressions.

**Table 11:** Wilcoxon rank-sum test results comparing L-SRTDE-ALP against L-SRTDE-ADCP and L-SRTDE-CP on 100-dimensional problems.

L-SRTDE-ALP vs.	+	–	≈
L-SRTDE-ADCP	8	2	19
L-SRTDE-CP	7	4	18

#### Discussion.

These controlled comparisons help clarify the impact of the proposed mechanism. In L-SRTDE-CP, perturbations are continuously sampled from a fixed Cauchy distribution: while the tails are heavy, their behavior is not adjustable. L-SRTDE-ADCP introduces adaptivity by varying the activation probability for each coordinate, thereby concentrating perturbations on dimensions that appear over-converged. However, the underlying perturbation distribution remains Cauchy, so the tail profile is unchanged. In contrast, L-SRTDE-ALP adds an extra degree of freedom by adapting the distribution shape according to fitness rank: higher-ranked individuals receive milder, less heavy-tailed perturbations to facilitate fine-tuning, while lower-ranked individuals are assigned heavier-tailed perturbations to encourage long-range exploration. This within-population heterogeneity in step-lengths provides a plausible explanation for the consistent (albeit sometimes modest) win–loss advantages observed across *D* = 30,50,100.

### Algorithm complexity

We evaluated the computational overhead of ALP following the timing procedure recommended by the CEC 2017 benchmark guidelines. The protocol separates (i) machine-dependent baseline time, (ii) the time required for pure objective evaluations, and (iii) the total runtime when an algorithm drives these evaluations.

First, we compute a calibration unit *T*_0_ using the standard micro-benchmark loop shown below.



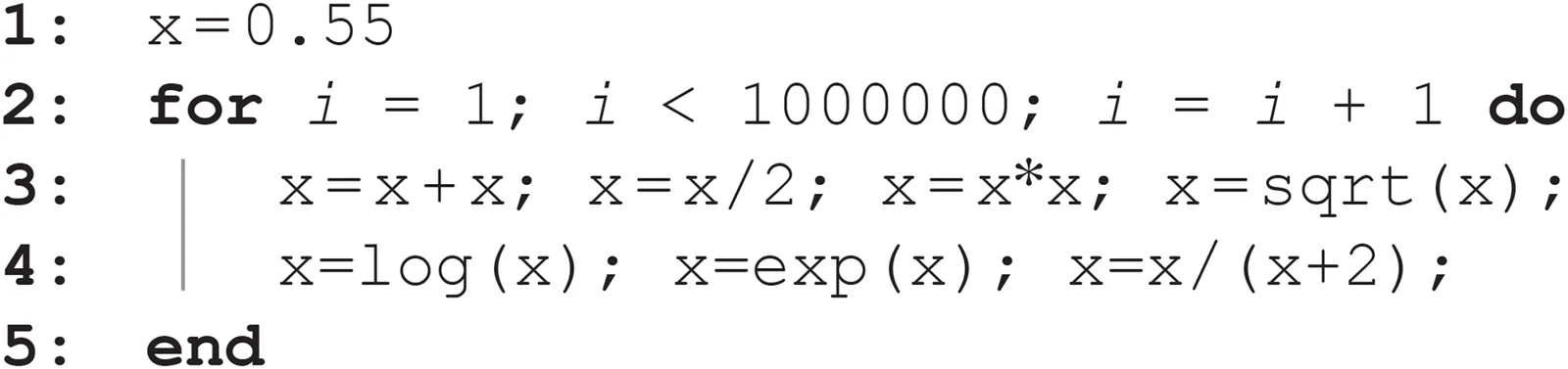



Next, we measure *T*_1_ as the elapsed time for exactly 200,000 calls to the CEC 2017 function *f*_18_ approximating the cost of objective evaluations alone. Finally, for each algorithm, we recorded the wall-clock time *T*_2_ required to complete the same 200,000 evaluations while executing the full evolutionary loop. Each measurement was repeated five times, and the average is denoted by ${\bar{T}}_{2}$. Following the CEC convention, the normalized algorithmic overhead is computed as


$$\begin{array}{c} \frac{{\bar{T}}_{2}-T_{1}}{T_{0}}, \end{array}$$

which estimates the additional computation beyond function evaluations and expresses it in units of the calibration loop.

Table 12 summarizes the resulting overheads for L-SRTDE-ALP, L-SRTDE-ADCP, and L-SRTDE-CP at D∈{10,30,50}. L-SRTDE-ALP achieves normalized overheads of 13.329603 (10D), 30.573932 (30D), and 30.081779 (50D), which are comparable to L-SRTDE-ADCP (13.159637, 27.122482, 33.397870) and L-SRTDE-CP (11.648815, 27.358663, 31.060145). Overall, the addition of rank computation and rank-conditioned α-stable sampling does not result in a marked runtime increase relative to the two Cauchy-based variants under this protocol.

**Table 12:** Algorithmic complexity results.

Algorithm	*D*	*T*_0_	*T*_1_	${\hat{T}}_{2}$	$\frac{{\hat{T}}_{2}-T_{1}}{T_{0}}$
L-SRTDE-ALP	10	0.023001	0.107212	0.413806	13.329603
	30		0.585013	1.288244	30.573932
	50		1.643128	2.335039	30.081779
L-SRTDE-ADCP	10	0.023001	0.107212	0.409897	13.159637
	30		0.585013	1.208857	27.122482
	50		1.643128	2.411312	33.397870
L-SRTDE-CP	10	0.023001	0.107212	0.375146	11.648815
	30		0.585013	1.214290	27.358663
	50		1.643128	2.357542	31.060145

#### Time complexity.

We adopt the same computational cost model used in the analyses of L-SRTDE and L-SRTDE-ADCP. Let *D* denote the problem dimension, $N_{g}$ the population size at generation *g* with $N_{g}\leq N_{\max}=20D$,$N_{FE}^{\max}$ the maximum number of function evaluations, and $T_{f}$ the cost of a single objective evaluation.

The core L-SRTDE loop, comprising mutation, crossover, selection, and the sorting or ranking operations required for elite management, incurs $𝒪(N_{g}D+N_{g}\log N_{g})$ arithmetic and comparison operations per generation, in addition to $𝒪(N_{g}T_{f})$ time for objective evaluations. Over the run, this results in the familiar overall bound of $𝒪(N_{FE}^{\max}D)+𝒪(N_{FE}^{\max}T_{f})$.

Relative to L-SRTDE-ADCP, ALP introduces only two additional computational steps. First, once fitness ranks are available, the stability parameters $\{\alpha_{i}^{g}{\}}_{i=1}^{N_{g}}$ are computed via a single pass through the population according to Eq. (9), which requires $𝒪(N_{g})$ time per generation. Second, ALP replaces the Cauchy random draw with a symmetric α-stable draw (e.g., using the Chambers–Mallows–Stuck sampler). This procedure involves a constant number of elementary operations and random variate generations per perturbed coordinate. As a result, the recombination step remains 𝒪(D) per individual and $𝒪(N_{g}D)$ per generation.

Consequently, L-SRTDE-ALP preserves the same asymptotic time complexity as both L-SRTDE and L-SRTDE-ADCP:


$$\begin{array}{c} T_{gen}^{ALP}=𝒪(N_{g}D+N_{g}\log N_{g}+N_{g}T_{f}),\;T_{ALP}=𝒪(N_{FE}^{\max}D)+𝒪(N_{FE}^{\max}T_{f}), \end{array}$$

while potentially incurring only a modest constant-factor overhead due to the more computationally intensive RNG required for α-stable sampling.

#### Space complexity.

The memory footprint is dominated by storing the population vectors, yielding a space complexity $𝒪(N_{g}D)$. ALP introduces an additional $𝒪(N_{g})$ storage cost for the stability parameters $\left\{\alpha_{i}^{g}\right\}$ and 𝒪(D) storage for $\left\{JR_{j}\right\}$; these additions do not alter the overall asymptotic space complexity.

### Analysis of parameter settings

We analyze the sensitivity of L-SRTDE-ALP to the stability-index bounds $\left(\alpha_{\min},\alpha_{\max}\right)$, which define the interval from which individual-specific stability indices $\alpha_{i}^{g}$ are assigned based on fitness rank. Smaller values of α induce heavier-tailed perturbations, enabling occasional long-range exploratory moves, whereas larger values of α lead to milder, more localized variations. Accordingly, the choice of $\left(\alpha_{\min},\alpha_{\max}\right)$ governs the degree to which ALP differentiates exploration-oriented and exploitation-oriented behaviors across the population.

We evaluated 20 parameter configurations, defined by $\alpha_{\min}\in \{0.3,0.5,0.7,1.0\}$ and $\alpha_{\max}\in \{1.0,1.3,1.5,1.7,2.0\}$ on the CEC 2017 benchmark functions with *D* = 50.

To assess whether performance differences among configurations are systematic across the full grid, we applied the Friedman test [43] together with the Iman–Davenport correction [47]. As reported in Table 13, neither test identifies a statistically significant difference among the 20 settings ($\chi_{F}^{2}=23.561$, *p* = 0.214; $F_{F}=1.251$, *p* = 0.211 at the 0.05 significance level), indicating that ALP is largely insensitive to the explored bound ranges. The low-level concordance (Kendall’s W≈0.043) further indicates that rank variations across configurations are modest.

**Table 13:** Omnibus nonparametric tests across 20 $\left(\alpha_{\min},\alpha_{\max}\right)$ configurations (CEC 2017, *D* = 50).

Friedman $\chi_{F}^{2}$	*p*	Iman–Davenport $F_{F}$	*p*	Kendall’s *W*
23.561	0.214	1.251	0.211	0.043

**Fig 10:**
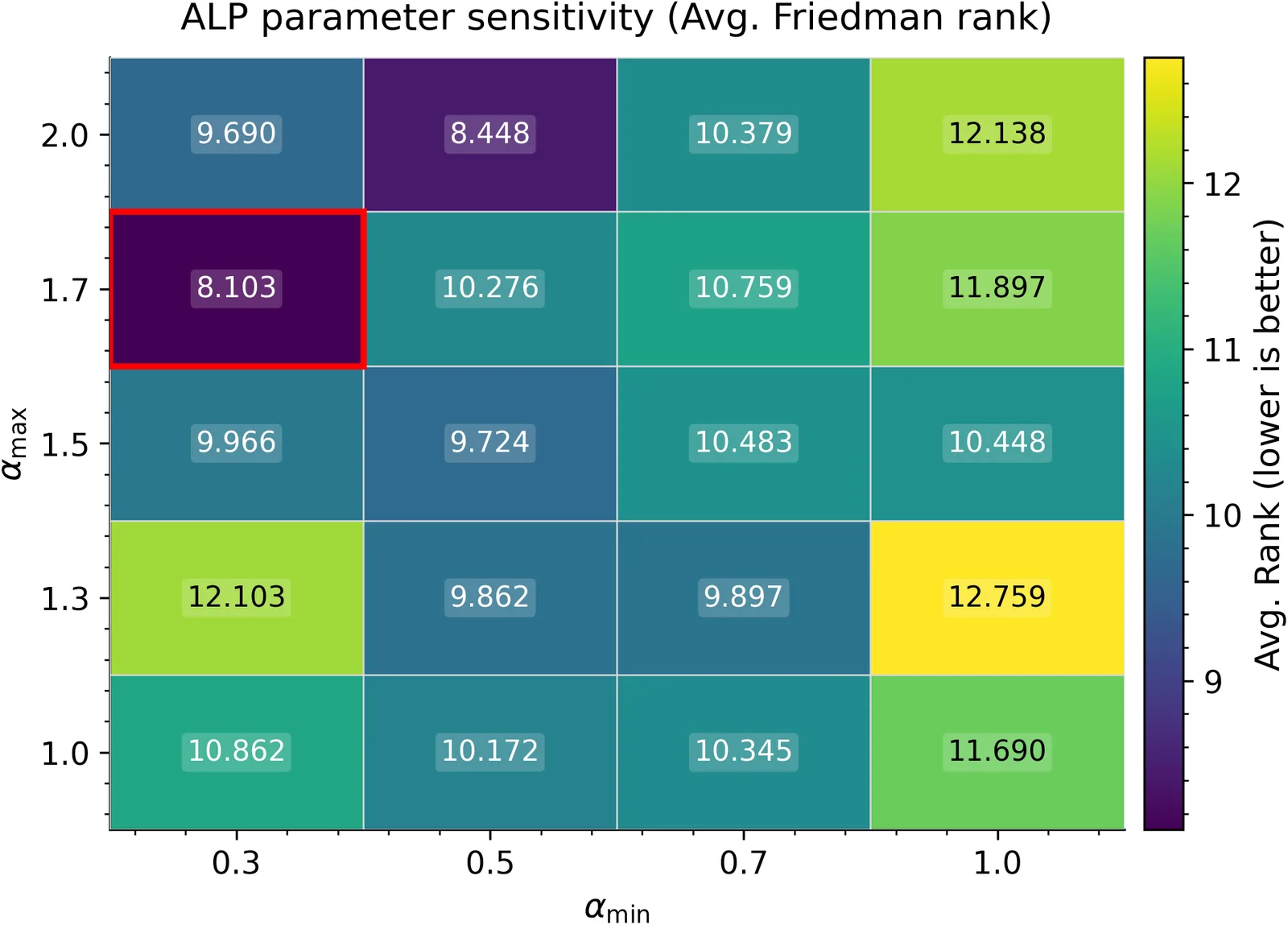
Sensitivity of ALP to $\left(\alpha_{\min},\alpha_{\max}\right)$ on the CEC 2017 suite (50D). Each tile corresponds to one tested configuration and is annotated with its average Friedman rank over 29 functions (lower is better). The best-performing configuration is highlighted.

Fig 10 summarizes the resulting response surface using the average Friedman rank for each pair. Although the omnibus tests are not statistically significant, the configuration $\left(\alpha_{\min}=0.3,\alpha_{\max}=1.7\right)$ attains the lowest mean rank (8.103) and is therefore adopted as the default setting for the remainder of the paper. A qualitative trend is also apparent: configurations with larger $\alpha_{\min}$ (e.g., $\alpha_{\min}=1.0$) tend to yield poorer ranks, which is consistent with excessively restricting heavy-tailed exploration for lower-ranked individuals.

Although the omnibus tests do not reveal statistically significant differences, we additionally report pairwise comparisons to verify that the chosen default is not a fragile selection. As shown in Table 14, most test functions fall into the tie category (22–28 ties), while the remaining wins and losses are few. Similarly, the post-hoc analyses reported in Table 15 show no statistically significant differences after family-wise error corrections (Bonferroni–Dunn, Holm, and Hochberg). Taken together, these results indicate that L-SRTDE-ALP remains stable across a broad range of $\left(\alpha_{\min},\alpha_{\max}\right)$ values, and that the choice (0.3,1.7) provides a robust default without requiring fine-tuning.

**Table 14:** Wilcoxon rank-sum summaries versus the default $\left(\alpha_{\min}=0.3,\alpha_{\max}=1.7\right)$ on 50-dimensional problems.

Comparison (vs. default)	+	–	≈
$\left(\alpha_{\min}=0.5,\alpha_{\max}=2.0\right)$	3	2	24
$\left(\alpha_{\min}=0.3,\alpha_{\max}=2.0\right)$	0	1	28
$\left(\alpha_{\min}=0.5,\alpha_{\max}=1.5\right)$	5	2	22
$\left(\alpha_{\min}=0.5,\alpha_{\max}=1.3\right)$	3	1	25

**Table 15:** Post-hoc comparisons against the default among the top five $\left(\alpha_{\min},\alpha_{\max}\right)$ configurations (50D).

Comparison (vs. default)	*z*	*p*	Bonf.	Holm	Hoch.
$\left(\alpha_{\min}=0.5,\alpha_{\max}=2.0\right)$	0.000	1.000	1.000	1.000	1.000
$\left(\alpha_{\min}=0.3,\alpha_{\max}=2.0\right)$	0.747	0.455	1.000	1.000	1.000
$\left(\alpha_{\min}=0.5,\alpha_{\max}=1.5\right)$	0.831	0.406	1.000	1.000	1.000
$\left(\alpha_{\min}=0.5,\alpha_{\max}=1.3\right)$	0.498	0.618	1.000	1.000	1.000

## Conclusion

This paper proposed a rank-conditioned α-stable perturbation mechanism for differential evolution. The main idea was to separate two aspects of heavy-tailed perturbation that are usually treated together: the probability of applying perturbation and the tail behavior of the perturbation distribution. Building on dimension-wise activation, the proposed method assigns each individual a symmetric α-stable perturbation kernel according to its fitness rank. In this way, highly ranked individuals are perturbed using lighter-tailed distributions suitable for local refinement, while lower-ranked individuals are assigned heavier-tailed distributions that support occasional long-distance exploration.

The proposed operator was embedded into L-SRTDE and evaluated on the IEEE CEC 2017 benchmark suite. The results indicate that rank-guided tail-shape adaptation can improve the robustness of heavy-tailed perturbation within a competitive DE framework. Across the tested dimensionalities, the proposed method obtained the best average Friedman rankings and favorable pairwise Wilcoxon outcomes against several recent DE variants. Controlled comparisons with conventional Cauchy perturbation and ADCP further suggest that adapting the perturbation distribution itself can complement probability-based activation. At the same time, comparisons with the base L-SRTDE show that the proposed mechanism should be viewed as a stable, low-overhead refinement rather than a complete redesign of the underlying optimizer.

Future work should examine whether rank-conditioned α-stable perturbation transfers to other DE frameworks and to other population-based metaheuristics. Another direction is to combine rank information with stagnation, diversity, or landscape indicators so that the stability index can respond not only to solution quality but also to the state of the search process. Applications to constrained, noisy, expensive, and real-world optimization problems would also help clarify the practical value of adaptive tail-shape control.

## Supporting information

S1 File Complete numerical results, additional convergence curves, and ECDF analyses for the CEC 2017 experiments. The file contains per-function best, median, mean, and standard deviation results, Wilcoxon rank-sum test outcomes, and additional convergence curves and ECDFs for the 30-, 50-, and 100-dimensional experiments.
(PDF)
