# Supplementary material for: A rank-conditioned α-stable perturbation for differential evolution: Separating perturbation frequency and tail heaviness
Source: PLoS One. 2026 Sep 9;21(9):e0357907. doi: 10.1371/journal.pone.0357907 (PMC13557417; doi:10.1371/journal.pone.0357907)
Supplement: Supplementary file 1 [file pone.0357907.s001.pdf]

Supplementary Material for
"A rank-conditioned α-stable perturbation for differential evolution: Separating perturbation frequency and tail heaviness"

S1 Table. Comparison of L-SRTDE-ALP with competing methods on the CEC 2017 test suite (30-dimensional). Results are based on Wilcoxon rank-sum tests at the significance level $\alpha = 0.05$.

	L-SRTDE-ALP MEAN (STD DEV)	L-SRTDE MEAN (STD DEV)	BWDE MEAN (STD DEV)	DPSDE MEAN (STD DEV)	FODE MEAN (STD DEV)	MSDE MEAN (STD DEV)
F1	0.000e+00 (0.000e+00)	0.000e+00 (0.000e+00) ≈	0.000e+00 (0.000e+00) ≈	0.000e+00 (0.000e+00) ≈	0.000e+00 (0.000e+00) ≈	0.000e+00 (0.000e+00) ≈
F3	0.000e+00 (0.000e+00)	0.000e+00 (0.000e+00) ≈	0.000e+00 (0.000e+00) ≈	0.000e+00 (0.000e+00) ≈	0.000e+00 (0.000e+00) ≈	0.000e+00 (0.000e+00) ≈
F4	1.545e+01 (1.675e+00)	5.856e+01 (6.459e-14) +	2.282e+01 (9.722e-01) +	2.310e+01 (1.029e+00) +	2.288e+01 (8.402e-01) +	2.330e+01 (8.754e-01) +
F5	2.503e+00 (1.449e+00)	2.205e+00 (7.787e-01) ≈	6.971e+00 (1.880e+00) +	7.421e+00 (1.963e+00) +	7.143e+00 (2.149e+00) +	1.098e+01 (4.947e+00) +
F6	5.626e-07 (6.538e-07)	0.000e+00 (0.000e+00) -	2.684e-09 (1.917e-08) -	2.684e-09 (1.917e-08) -	0.000e+00 (0.000e+00) -	0.000e+00 (0.000e+00) -
F7	3.568e+01 (1.887e+00)	3.497e+01 (1.088e+00) ≈	3.827e+01 (1.896e+00) +	3.728e+01 (1.777e+00) +	3.786e+01 (1.791e+00) +	4.081e+01 (6.321e+00) +
F8	2.302e+00 (1.752e+00)	2.009e+00 (6.745e-01) ≈	6.817e+00 (1.711e+00) +	7.285e+00 (1.614e+00) +	7.126e+00 (1.881e+00) +	1.091e+01 (3.848e+00) +
F9	0.000e+00 (0.000e+00)	0.000e+00 (0.000e+00) ≈	0.000e+00 (0.000e+00) ≈	0.000e+00 (0.000e+00) ≈	0.000e+00 (0.000e+00) ≈	0.000e+00 (0.000e+00) ≈
F10	3.560e+02 (2.464e+02)	2.666e+02 (1.681e+02) ≈	1.411e+03 (2.548e+02) +	1.470e+03 (3.077e+02) +	1.407e+03 (2.987e+02) +	9.541e+02 (5.454e+02) +
F11	1.680e+00 (2.076e+00)	1.778e+00 (1.830e+00) ≈	3.155e+00 (3.196e+00) +	4.002e+00 (5.135e+00) +	3.895e+00 (3.230e+00) +	4.735e+00 (2.469e+00) +
F12	1.003e+00 (1.582e+00)	1.789e+00 (1.799e+00) +	3.354e+02 (2.258e+02) +	3.039e+02 (1.897e+02) +	3.479e+02 (1.892e+02) +	3.485e+02 (1.974e+02) +
F13	1.046e+01 (6.366e+00)	8.748e+00 (7.346e+00) ≈	1.699e+01 (6.375e+00) +	1.617e+01 (6.567e+00) +	1.684e+01 (5.130e+00) +	1.773e+01 (5.545e+00) +
F14	3.710e+00 (7.742e+00)	8.482e+00 (1.067e+01) ≈	1.995e+01 (5.940e+00) +	1.983e+01 (6.794e+00) +	1.743e+01 (8.148e+00) +	2.234e+01 (1.610e+00) +
F15	4.000e-01 (6.358e-02)	4.420e-01 (2.676e-02) +	1.398e+00 (7.889e-01) +	1.488e+00 (1.001e+00) +	1.430e+00 (1.129e+00) +	1.657e+00 (1.089e+00) +
F16	5.120e+00 (2.785e+00)	6.649e+00 (6.083e+00) ≈	1.273e+01 (5.151e+00) +	1.721e+01 (2.693e+01) +	2.518e+01 (3.798e+01) +	2.303e+01 (3.952e+01) +
F17	2.159e+01 (6.255e+00)	2.188e+01 (4.202e+00) ≈	2.806e+01 (9.861e+00) +	3.271e+01 (9.411e+00) +	2.901e+01 (7.282e+00) +	2.494e+01 (5.676e+00) +
F18	1.623e+01 (8.340e+00)	2.068e+01 (5.177e-02) +	2.061e+01 (3.236e-01) ≈	2.077e+01 (4.598e-01) +	1.996e+01 (3.889e+00) +	2.064e+01 (2.994e+00) ≈
F19	1.962e+00 (4.593e-01)	1.963e+00 (5.082e-01) ≈	3.291e+00 (8.202e-01) +	3.499e+00 (9.716e-01) +	3.299e+00 (1.104e+00) +	3.535e+00 (1.166e+00) +
F20	6.427e+00 (1.786e+01)	3.932e+01 (5.033e+01) +	2.628e+01 (5.301e+00) +	2.497e+01 (5.689e+00) +	2.640e+01 (5.860e+00) +	2.517e+01 (2.636e+01) +
F21	2.024e+02 (1.405e+00)	2.022e+02 (1.256e+00) ≈	2.070e+02 (1.886e+00) +	2.073e+02 (2.003e+00) +	2.074e+02 (1.695e+00) +	2.109e+02 (4.331e+00) +
F22	1.000e+02 (0.000e+00)	1.000e+02 (0.000e+00) ≈	1.000e+02 (0.000e+00) ≈	1.000e+02 (0.000e+00) ≈	1.000e+02 (0.000e+00) ≈	1.000e+02 (0.000e+00) ≈
F23	3.382e+02 (3.546e+00)	3.392e+02 (3.030e+00) ≈	3.516e+02 (4.608e+00) +	3.503e+02 (3.439e+00) +	3.501e+02 (3.029e+00) +	3.552e+02 (6.071e+00) +
F24	4.158e+02 (3.929e+00)	4.186e+02 (3.356e+00) +	4.269e+02 (2.524e+00) +	4.265e+02 (2.014e+00) +	4.267e+02 (2.799e+00) +	4.303e+02 (4.890e+00) +
F25	3.789e+02 (8.131e-01)	3.867e+02 (4.808e-03) +	3.787e+02 (1.581e-01) +	3.787e+02 (1.636e-01) +	3.787e+02 (1.767e-01) +	3.784e+02 (5.512e-02) -
F26	6.218e+02 (1.373e+02)	5.921e+02 (1.516e+02) ≈	9.244e+02 (4.184e+01) +	9.306e+02 (4.875e+01) +	9.199e+02 (4.301e+01) +	9.465e+02 (5.851e+01) +
F27	4.591e+02 (5.312e+00)	4.753e+02 (6.000e+00) +	4.766e+02 (5.676e+00) +	4.787e+02 (6.047e+00) +	4.788e+02 (5.387e+00) +	4.797e+02 (2.003e+01) +
F28	3.020e+02 (1.446e+01)	3.081e+02 (2.804e+01) ≈	3.152e+02 (3.857e+01) -	3.130e+02 (3.595e+01) -	3.217e+02 (4.445e+01) -	3.271e+02 (5.101e+01) -
F29	3.707e+02 (8.762e+00)	3.995e+02 (4.513e+00) +	4.020e+02 (3.615e+01) +	3.995e+02 (3.407e+01) +	4.017e+02 (2.443e+01) +	3.805e+02 (2.138e+01) ≈
F30	3.737e+02 (4.206e+01)	1.987e+03 (5.104e+00) +	8.834e+02 (3.712e+02) +	8.534e+02 (3.415e+02) +	8.726e+02 (3.404e+02) +	5.478e+02 (3.085e+02) +
+/-/≈	-	10/1/18	22/2/5	23/2/4	23/2/4	20/3/6

	RDE MEAN (STD DEV)	RSDE MEAN (STD DEV)	SMLDE MEAN (STD DEV)	EBOwithCMAR MEAN (STD DEV)	jSO MEAN (STD DEV)	LSHADE-SPACMA MEAN (STD DEV)
F1	0.000e+00 (0.000e+00) ≈	0.000e+00 (0.000e+00) ≈	0.000e+00 (0.000e+00) ≈	0.000e+00 (0.000e+00) ≈	0.000e+00 (0.000e+00) ≈	0.000e+00 (0.000e+00) ≈
F3	0.000e+00 (0.000e+00) ≈	0.000e+00 (0.000e+00) ≈	0.000e+00 (0.000e+00) ≈	0.000e+00 (0.000e+00) ≈	0.000e+00 (0.000e+00) ≈	0.000e+00 (0.000e+00) ≈
F4	2.146e+01 (1.313e+00) +	2.279e+01 (1.175e+00) +	2.309e+01 (8.096e-01) +	5.645e+01 (1.115e+01) +	5.867e+01 (7.780e-01) +	5.856e+01 (0.000e+00) +
F5	7.163e+00 (2.061e+00) +	6.616e+00 (2.151e+00) +	6.877e+00 (2.345e+00) +	2.776e+00 (1.744e+00) ≈	8.557e+00 (2.098e+00) +	3.447e+00 (2.630e+00) +
F6	0.000e+00 (0.000e+00) -	0.000e+00 (0.000e+00) -	2.684e-09 (1.917e-08) -	0.000e+00 (0.000e+00) -	6.039e-09 (2.712e-08) -	0.000e+00 (0.000e+00) -
F7	3.852e+01 (1.662e+00) +	3.706e+01 (1.554e+00) +	3.808e+01 (1.717e+00) +	3.346e+01 (8.369e-01) -	3.893e+01 (1.459e+00) +	3.380e+01 (8.490e-01) -
F8	7.024e+00 (1.751e+00) +	6.701e+00 (2.042e+00) +	7.208e+00 (1.558e+00) +	2.022e+00 (1.317e+00) ≈	9.092e+00 (1.840e+00) +	3.200e+00 (1.597e+00) +
F9	0.000e+00 (0.000e+00) ≈	0.000e+00 (0.000e+00) ≈	0.000e+00 (0.000e+00) ≈	0.000e+00 (0.000e+00) ≈	0.000e+00 (0.000e+00) ≈	0.000e+00 (0.000e+00) ≈
F10	1.419e+03 (2.138e+02) +	1.403e+03 (2.959e+02) +	1.405e+03 (2.893e+02) +	1.408e+03 (2.149e+02) +	1.528e+03 (2.772e+02) +	1.441e+03 (2.520e+02) +
F11	4.891e+00 (5.599e+00) +	3.140e+00 (2.685e+00) +	4.789e+00 (8.516e+00) +	4.493e+00 (8.768e+00) +	3.037e+00 (2.646e+00) +	1.780e+01 (2.482e+01) +
F12	2.728e+02 (1.450e+02) +	1.608e+02 (1.174e+02) +	3.164e+02 (1.868e+02) +	4.629e+02 (2.629e+02) +	1.704e+02 (1.019e+02) +	6.152e+02 (3.132e+02) +
F13	1.423e+01 (5.886e+00) +	1.501e+01 (6.531e+00) +	1.741e+01 (6.079e+00) +	1.489e+01 (6.248e+00) +	1.484e+01 (4.831e+00) +	1.462e+01 (4.985e+00) +
F14	1.664e+01 (9.232e+00) +	1.748e+01 (8.488e+00) +	2.040e+01 (5.819e+00) +	2.190e+01 (3.839e+00) +	2.183e+01 (1.246e+00) +	2.338e+01 (1.693e+00) +
F15	1.439e+00 (1.035e+00) +	1.263e+00 (8.128e-01) +	1.426e+00 (1.309e+00) +	3.685e+00 (2.152e+00) +	1.088e+00 (6.913e-01) +	4.459e+00 (2.292e+00) +
F16	2.518e+01 (4.715e+01) +	2.562e+01 (4.602e+01) +	2.621e+01 (3.917e+01) +	4.262e+01 (5.694e+01) +	7.892e+01 (8.477e+01) +	2.523e+01 (1.753e+01) +
F17	3.556e+01 (7.449e+00) +	3.266e+01 (8.542e+00) +	3.129e+01 (1.004e+01) +	2.975e+01 (7.496e+00) +	3.292e+01 (8.077e+00) +	3.041e+01 (8.123e+00) +
F18	2.001e+01 (4.041e+00) +	2.060e+01 (2.529e-01) ≈	2.075e+01 (4.698e-01) +	2.214e+01 (1.093e+00) +	2.041e+01 (2.873e+00) +	2.343e+01 (1.859e+00) +
F19	3.266e+00 (1.046e+00) +	3.445e+00 (8.871e-01) +	3.432e+00 (8.747e-01) +	8.040e+00 (2.278e+00) +	4.503e+00 (1.732e+00) +	1.032e+01 (2.239e+00) +
F20	2.760e+01 (8.848e+00) +	2.474e+01 (7.391e+00) +	2.637e+01 (6.361e+00) +	3.572e+01 (7.495e+00) +	2.937e+01 (5.855e+00) +	8.383e+01 (5.546e+01) +
F21	2.071e+02 (2.625e+00) +	2.066e+02 (1.849e+00) +	2.073e+02 (2.052e+00) +	1.989e+02 (2.024e+01) ≈	2.093e+02 (1.955e+00) +	2.074e+02 (3.516e+00) +
F22	1.000e+02 (0.000e+00) ≈	1.000e+02 (0.000e+00) ≈	1.000e+02 (0.000e+00) ≈	1.000e+02 (0.000e+00) ≈	1.000e+02 (0.000e+00) ≈	1.000e+02 (0.000e+00) ≈
F23	3.507e+02 (4.031e+00) +	3.520e+02 (5.707e+00) +	3.511e+02 (4.711e+00) +	3.512e+02 (3.512e+00) +	3.507e+02 (3.299e+00) +	3.555e+02 (3.417e+00) +
F24	4.250e+02 (3.091e+00) +	4.275e+02 (2.693e+00) +	4.269e+02 (3.100e+00) +	4.181e+02 (4.546e+01) +	4.265e+02 (2.466e+00) +	4.292e+02 (2.816e+00) +
F25	3.787e+02 (1.619e-01) +	3.788e+02 (2.312e-01) +	3.786e+02 (9.440e-02) +	3.865e+02 (7.565e-01) +	3.867e+02 (7.720e-03) +	3.867e+02 (1.062e-02) +
F26	9.098e+02 (4.021e+01) +	9.188e+02 (5.108e+01) +	9.248e+02 (4.721e+01) +	5.373e+02 (3.058e+02) ≈	9.202e+02 (4.295e+01) +	9.530e+02 (5.262e+01) +
F27	4.736e+02 (5.655e+00) +	4.790e+02 (5.769e+00) +	4.748e+02 (5.925e+00) +	5.024e+02 (4.027e+00) +	4.974e+02 (7.002e+00) +	5.051e+02 (6.373e+00) +
F28	3.197e+02 (4.298e+01) -	3.383e+02 (5.537e+01) -	3.243e+02 (4.692e+01) -	3.083e+02 (2.880e+01) ≈	3.087e+02 (3.025e+01) ≈	3.112e+02 (3.423e+01) ≈
F29	4.093e+02 (2.959e+01) +	4.058e+02 (2.551e+01) +	3.899e+02 (3.063e+01) +	4.328e+02 (1.131e+01) +	4.337e+02 (1.364e+01) +	4.445e+02 (1.314e+01) +
F30	7.557e+02 (3.040e+02) +	9.848e+02 (3.825e+02) +	7.141e+02 (2.361e+02) +	1.988e+03 (4.211e+01) +	1.971e+03 (1.896e+01) +	2.006e+03 (6.280e+01) +
+/-/≈	23/2/4	22/2/5	23/2/4	18/2/9	23/1/5	22/2/5

Entries are mean (standard deviation) objective values computed over independent runs. Symbols denote the outcome of two-sided Wilcoxon rank-sum tests using L-SRTDE-ALP as the reference: "+" indicates that L-SRTDE-ALP performed significantly better than the comparator; "-" indicates that L-SRTDE-ALP performed significantly worse; and "≈" indicates no statistically significant difference. The final row reports per-configuration counts of wins/losses/ties versus the reference (format: wins/losses/ties).

S2 Table. Best and median objective errors obtained by the compared algorithms on the 30-dimensional CEC 2017 test suite.

	L-SRTDE-ALP BEST / MEDIAN	L-SRTDE BEST / MEDIAN	BWDE BEST / MEDIAN	DPSDE BEST / MEDIAN	FODE BEST / MEDIAN	MSDE BEST / MEDIAN
F1	0.000e+00 / 0.000e+00	0.000e+00 / 0.000e+00	0.000e+00 / 0.000e+00	0.000e+00 / 0.000e+00	0.000e+00 / 0.000e+00	0.000e+00 / 0.000e+00
F3	0.000e+00 / 0.000e+00	0.000e+00 / 0.000e+00	0.000e+00 / 0.000e+00	0.000e+00 / 0.000e+00	0.000e+00 / 0.000e+00	0.000e+00 / 0.000e+00
F4	1.454e+01 / 1.465e+01	5.856e+01 / 5.856e+01	2.069e+01 / 2.302e+01	2.080e+01 / 2.295e+01	2.021e+01 / 2.307e+01	2.173e+01 / 2.325e+01
F5	0.000e+00 / 2.985e+00	9.950e-01 / 1.990e+00	1.990e+00 / 6.966e+00	3.980e+00 / 6.977e+00	1.991e+00 / 6.965e+00	1.990e+00 / 1.094e+01
F6	0.000e+00 / 3.080e-07	0.000e+00 / 0.000e+00	0.000e+00 / 0.000e+00	0.000e+00 / 0.000e+00	0.000e+00 / 0.000e+00	0.000e+00 / 0.000e+00
F7	3.296e+01 / 3.539e+01	3.358e+01 / 3.424e+01	3.452e+01 / 3.807e+01	3.438e+01 / 3.722e+01	3.516e+01 / 3.776e+01	3.475e+01 / 3.879e+01
F8	0.000e+00 / 1.990e+00	9.950e-01 / 1.990e+00	3.980e+00 / 6.965e+00	3.980e+00 / 6.968e+00	3.980e+00 / 6.968e+00	9.950e-01 / 9.950e+00
F9	0.000e+00 / 0.000e+00	0.000e+00 / 0.000e+00	0.000e+00 / 0.000e+00	0.000e+00 / 0.000e+00	0.000e+00 / 0.000e+00	0.000e+00 / 0.000e+00
F10	1.419e+01 / 2.759e+02	2.610e+00 / 2.496e+02	9.060e+02 / 1.413e+03	6.207e+02 / 1.495e+03	5.739e+02 / 1.401e+03	8.663e+00 / 8.902e+02
F11	0.000e+00 / 0.000e+00	0.000e+00 / 9.950e-01	0.000e+00 / 1.990e+00	0.000e+00 / 2.985e+00	0.000e+00 / 3.980e+00	9.950e-01 / 4.982e+00
F12	2.082e-01 / 3.477e-01	2.776e-01 / 4.164e-01	1.138e+01 / 2.537e+02	7.658e+00 / 2.537e+02	4.003e+00 / 2.746e+02	1.321e+01 / 3.503e+02
F13	0.000e+00 / 1.384e+01	0.000e+00 / 1.383e+01	9.955e-01 / 1.924e+01	3.310e-01 / 1.737e+01	1.085e+00 / 1.731e+01	0.000e+00 / 1.939e+01
F14	0.000e+00 / 0.000e+00	0.000e+00 / 0.000e+00	1.068e+00 / 2.130e+01	3.640e-04 / 2.206e+01	9.955e-01 / 2.103e+01	2.001e+01 / 2.202e+01
F15	2.296e-01 / 4.150e-01	4.032e-01 / 4.343e-01	3.045e-01 / 1.456e+00	3.445e-01 / 1.447e+00	3.170e-01 / 1.427e+00	1.760e-01 / 1.451e+00
F16	1.549e+00 / 5.894e+00	9.586e-01 / 4.809e+00	4.318e+00 / 1.269e+01	3.391e+00 / 1.048e+01	4.024e+00 / 1.167e+01	2.271e+00 / 8.872e+00
F17	2.023e+00 / 2.199e+01	1.890e+00 / 2.304e+01	7.969e+00 / 3.089e+01	1.012e+01 / 3.230e+01	1.053e+01 / 3.042e+01	2.560e+00 / 2.495e+01
F18	4.694e-01 / 2.053e+01	2.057e+01 / 2.068e+01	2.028e+01 / 2.053e+01	2.031e+01 / 2.058e+01	4.792e-01 / 2.054e+01	3.455e-01 / 2.052e+01
F19	1.039e+00 / 1.865e+00	1.460e+00 / 1.915e+00	1.828e+00 / 3.152e+00	1.889e+00 / 3.171e+00	2.081e+00 / 3.034e+00	1.351e+00 / 3.418e+00
F20	0.000e+00 / 4.163e-01	3.123e-01 / 2.069e+01	3.308e+00 / 2.676e+01	6.596e+00 / 2.465e+01	6.094e+00 / 2.717e+01	1.307e+00 / 2.152e+01
F21	2.000e+02 / 2.020e+02	2.000e+02 / 2.030e+02	2.030e+02 / 2.069e+02	2.030e+02 / 2.073e+02	2.032e+02 / 2.074e+02	2.044e+02 / 2.097e+02
F22	1.000e+02 / 1.000e+02	1.000e+02 / 1.000e+02	1.000e+02 / 1.000e+02	1.000e+02 / 1.000e+02	1.000e+02 / 1.000e+02	1.000e+02 / 1.000e+02
F23	3.240e+02 / 3.385e+02	3.336e+02 / 3.382e+02	3.451e+02 / 3.506e+02	3.448e+02 / 3.498e+02	3.428e+02 / 3.502e+02	3.431e+02 / 3.560e+02
F24	4.060e+02 / 4.163e+02	4.100e+02 / 4.191e+02	4.226e+02 / 4.265e+02	4.219e+02 / 4.262e+02	4.223e+02 / 4.264e+02	4.213e+02 / 4.290e+02
F25	3.783e+02 / 3.785e+02	3.867e+02 / 3.867e+02	3.784e+02 / 3.786e+02	3.785e+02 / 3.787e+02	3.784e+02 / 3.787e+02	3.783e+02 / 3.784e+02
F26	4.000e+02 / 6.536e+02	4.000e+02 / 6.522e+02	8.049e+02 / 9.258e+02	8.169e+02 / 9.272e+02	8.287e+02 / 9.223e+02	8.167e+02 / 9.427e+02
F27	4.490e+02 / 4.597e+02	4.658e+02 / 4.731e+02	4.655e+02 / 4.764e+02	4.609e+02 / 4.789e+02	4.684e+02 / 4.777e+02	4.461e+02 / 4.747e+02
F28	3.000e+02 / 3.000e+02	3.000e+02 / 3.000e+02	3.000e+02 / 3.000e+02	3.000e+02 / 3.000e+02	3.000e+02 / 3.000e+02	3.000e+02 / 3.000e+02
F29	3.529e+02 / 3.700e+02	3.925e+02 / 4.006e+02	2.658e+02 / 3.989e+02	2.568e+02 / 3.989e+02	3.563e+02 / 4.022e+02	3.477e+02 / 3.742e+02
F30	3.102e+02 / 3.646e+02	1.955e+03 / 1.988e+03	4.315e+02 / 7.530e+02	3.967e+02 / 7.898e+02	3.896e+02 / 7.808e+02	3.205e+02 / 4.352e+02
N_{med}	19	13	6	6	6	8
N_{best}	16	12	7	8	7	10

	RDE BEST / MEDIAN	RSDE BEST / MEDIAN	SMLDE BEST / MEDIAN	EBOwithCMAR BEST / MEDIAN	jSO BEST / MEDIAN	LSHADE-SPACMA BEST / MEDIAN
F1	0.000e+00 / 0.000e+00	0.000e+00 / 0.000e+00	0.000e+00 / 0.000e+00	0.000e+00 / 0.000e+00	0.000e+00 / 0.000e+00	0.000e+00 / 0.000e+00
F3	0.000e+00 / 0.000e+00	0.000e+00 / 0.000e+00	0.000e+00 / 0.000e+00	0.000e+00 / 0.000e+00	0.000e+00 / 0.000e+00	0.000e+00 / 0.000e+00
F4	1.473e+01 / 2.116e+01	2.076e+01 / 2.288e+01	2.110e+01 / 2.307e+01	0.000e+00 / 5.856e+01	5.856e+01 / 5.856e+01	5.856e+01 / 5.856e+01
F5	1.990e+00 / 6.965e+00	1.990e+00 / 5.971e+00	2.985e+00 / 6.965e+00	0.000e+00 / 2.985e+00	3.980e+00 / 8.017e+00	0.000e+00 / 2.985e+00
F6	0.000e+00 / 0.000e+00	0.000e+00 / 0.000e+00	0.000e+00 / 0.000e+00	0.000e+00 / 0.000e+00	0.000e+00 / 0.000e+00	0.000e+00 / 0.000e+00
F7	3.532e+01 / 3.821e+01	3.406e+01 / 3.694e+01	3.549e+01 / 3.767e+01	3.166e+01 / 3.336e+01	3.611e+01 / 3.906e+01	3.198e+01 / 3.364e+01
F8	3.980e+00 / 6.965e+00	1.990e+00 / 6.965e+00	3.980e+00 / 6.966e+00	0.000e+00 / 1.990e+00	4.975e+00 / 8.956e+00	0.000e+00 / 2.985e+00
F9	0.000e+00 / 0.000e+00	0.000e+00 / 0.000e+00	0.000e+00 / 0.000e+00	0.000e+00 / 0.000e+00	0.000e+00 / 0.000e+00	0.000e+00 / 0.000e+00
F10	1.006e+03 / 1.416e+03	5.136e+02 / 1.425e+03	7.144e+02 / 1.472e+03	9.836e+02 / 1.378e+03	1.039e+03 / 1.493e+03	7.335e+02 / 1.442e+03
F11	0.000e+00 / 2.985e+00	0.000e+00 / 1.990e+00	0.000e+00 / 2.985e+00	0.000e+00 / 1.990e+00	0.000e+00 / 1.990e+00	0.000e+00 / 5.970e+00
F12	3.470e-01 / 2.556e+02	4.003e+00 / 1.394e+02	1.145e+01 / 2.670e+02	1.504e+01 / 4.351e+02	3.864e+00 / 1.389e+02	1.297e+02 / 5.582e+02
F13	9.950e-01 / 1.593e+01	1.119e+00 / 1.558e+01	9.958e-01 / 1.948e+01	1.384e+00 / 1.659e+01	9.930e-01 / 1.587e+01	0.000e+00 / 1.577e+01
F14	2.070e-02 / 2.108e+01	1.812e-01 / 2.130e+01	3.890e-02 / 2.200e+01	2.515e+00 / 2.206e+01	2.006e+01 / 2.124e+01	2.100e+01 / 2.300e+01
F15	3.341e-01 / 1.447e+00	3.748e-01 / 1.390e+00	3.201e-01 / 1.262e+00	2.785e-01 / 3.392e+00	3.249e-01 / 7.794e-01	1.126e+00 / 4.169e+00
F16	4.190e+00 / 1.395e+01	2.569e+00 / 1.243e+01	4.302e+00 / 1.249e+01	3.253e+00 / 2.106e+01	4.018e+00 / 2.593e+01	2.336e+00 / 1.866e+01
F17	1.365e+01 / 3.533e+01	8.176e+00 / 3.242e+01	6.518e+00 / 3.296e+01	9.951e+00 / 3.102e+01	1.163e+01 / 3.459e+01	1.123e+01 / 3.048e+01
F18	2.587e-01 / 2.055e+01	2.029e+01 / 2.053e+01	2.032e+01 / 2.057e+01	2.040e+01 / 2.198e+01	4.859e-01 / 2.065e+01	2.041e+01 / 2.331e+01
F19	1.713e+00 / 2.969e+00	2.036e+00 / 3.264e+00	2.386e+00 / 3.069e+00	3.946e+00 / 8.143e+00	2.068e+00 / 4.085e+00	3.148e+00 / 1.038e+01
F20	2.511e+00 / 2.567e+01	2.864e+00 / 2.482e+01	8.401e+00 / 2.642e+01	2.339e+01 / 3.486e+01	1.367e+01 / 2.930e+01	2.465e+01 / 5.849e+01
F21	2.000e+02 / 2.070e+02	2.033e+02 / 2.064e+02	2.031e+02 / 2.070e+02	1.000e+02 / 2.029e+02	2.051e+02 / 2.095e+02	2.000e+02 / 2.064e+02
F22	1.000e+02 / 1.000e+02	1.000e+02 / 1.000e+02	1.000e+02 / 1.000e+02	1.000e+02 / 1.000e+02	1.000e+02 / 1.000e+02	1.000e+02 / 1.000e+02
F23	3.389e+02 / 3.505e+02	3.438e+02 / 3.508e+02	3.416e+02 / 3.509e+02	3.450e+02 / 3.506e+02	3.423e+02 / 3.505e+02	3.468e+02 / 3.556e+02
F24	4.179e+02 / 4.251e+02	4.227e+02 / 4.271e+02	4.220e+02 / 4.267e+02	1.000e+02 / 4.243e+02	4.220e+02 / 4.267e+02	4.231e+02 / 4.292e+02
F25	3.784e+02 / 3.787e+02	3.784e+02 / 3.787e+02	3.783e+02 / 3.786e+02	3.835e+02 / 3.867e+02	3.867e+02 / 3.867e+02	3.867e+02 / 3.867e+02
F26	8.395e+02 / 9.064e+02	8.268e+02 / 9.176e+02	8.395e+02 / 9.258e+02	2.000e+02 / 3.000e+02	8.392e+02 / 9.304e+02	8.318e+02 / 9.548e+02
F27	4.580e+02 / 4.736e+02	4.691e+02 / 4.789e+02	4.608e+02 / 4.743e+02	4.925e+02 / 5.030e+02	4.820e+02 / 4.947e+02	4.842e+02 / 5.052e+02
F28	3.000e+02 / 3.000e+02	3.000e+02 / 3.000e+02	3.000e+02 / 3.000e+02	3.000e+02 / 3.000e+02	3.000e+02 / 3.000e+02	3.000e+02 / 3.000e+02
F29	3.629e+02 / 4.125e+02	3.643e+02 / 4.036e+02	2.572e+02 / 3.834e+02	4.074e+02 / 4.340e+02	3.567e+02 / 4.331e+02	4.229e+02 / 4.431e+02
F30	4.566e+02 / 6.342e+02	4.781e+02 / 8.701e+02	4.730e+02 / 6.141e+02	1.942e+03 / 1.972e+03	1.955e+03 / 1.971e+03	1.941e+03 / 1.980e+03
N_{med}	6	6	6	8	6	6
N_{best}	8	7	7	14	7	10

Entries are best / median objective errors computed over 51 independent runs. Lower values indicate better performance. Best denotes the minimum error observed across the 51 runs. N_{med} and N_{best} denote the numbers of functions for which each algorithm attained the lowest median and lowest best value, respectively. Ties are counted for all tied algorithms.

S3 Table. Comparison of L-SRTDE-ALP with competing methods on the CEC 2017 test suite (50-dimensional). Results are based on Wilcoxon rank-sum tests at the significance level $\alpha = 0.05$.

	L-SRTDE-ALP MEAN (STD DEV)	L-SRTDE MEAN (STD DEV)	BWDE MEAN (STD DEV)	DPSDE MEAN (STD DEV)	FODE MEAN (STD DEV)	MSDE MEAN (STD DEV)
F1	0.000e+00 (0.000e+00)	0.000e+00 (0.000e+00) ≈	0.000e+00 (0.000e+00) ≈	0.000e+00 (0.000e+00) ≈	0.000e+00 (0.000e+00) ≈	0.000e+00 (0.000e+00) ≈
F3	0.000e+00 (0.000e+00)	0.000e+00 (0.000e+00) ≈	0.000e+00 (0.000e+00) ≈	0.000e+00 (0.000e+00) ≈	0.000e+00 (0.000e+00) ≈	0.000e+00 (0.000e+00) ≈
F4	2.523e+01 (1.796e+01)	2.851e+01 (2.512e-14) +	5.758e+01 (4.942e+01) +	5.523e+01 (4.758e+01) +	6.221e+01 (5.197e+01) +	5.734e+01 (3.949e+01) +
F5	6.301e+00 (2.204e+00)	5.775e+00 (1.780e+00) ≈	1.392e+01 (3.467e+00) +	1.448e+01 (2.957e+00) +	1.475e+01 (3.079e+00) +	1.496e+01 (4.581e+00) +
F6	1.943e-05 (1.016e-05)	1.079e-06 (1.228e-06) -	6.754e-08 (1.532e-07) -	1.951e-08 (6.731e-08) -	2.102e-08 (7.628e-08) -	2.563e-08 (1.093e-07) -
F7	6.046e+01 (2.456e+00)	5.994e+01 (4.080e+00) -	6.648e+01 (4.523e+00) +	6.690e+01 (3.948e+00) +	6.639e+01 (3.367e+00) +	6.412e+01 (4.702e+00) +
F8	6.399e+00 (2.118e+00)	5.482e+00 (2.091e+00) ≈	1.442e+01 (3.349e+00) +	1.480e+01 (3.255e+00) +	1.434e+01 (2.954e+00) +	1.609e+01 (5.631e+00) +
F9	0.000e+00 (0.000e+00)	0.000e+00 (0.000e+00) ≈	0.000e+00 (0.000e+00) ≈	0.000e+00 (0.000e+00) ≈	0.000e+00 (0.000e+00) ≈	0.000e+00 (0.000e+00) ≈
F10	5.045e+02 (3.071e+02)	3.020e+02 (1.555e+02) -	3.083e+03 (3.671e+02) +	3.316e+03 (5.141e+02) +	3.117e+03 (4.396e+02) +	2.268e+03 (1.080e+03) +
F11	1.121e+01 (9.163e-01)	1.903e+01 (8.807e-01) +	2.210e+01 (3.674e+00) +	2.203e+01 (4.366e+00) +	2.124e+01 (4.239e+00) +	2.002e+01 (3.914e+00) +
F12	1.016e+02 (7.437e+01)	9.512e+01 (6.150e+01) ≈	1.817e+03 (4.261e+02) +	1.829e+03 (3.707e+02) +	1.898e+03 (3.775e+02) +	1.904e+03 (4.434e+02) +
F13	4.161e+00 (2.114e+00)	4.236e+00 (2.287e+00) ≈	3.863e+01 (2.729e+01) +	4.534e+01 (2.571e+01) +	3.768e+01 (2.685e+01) +	3.882e+01 (2.352e+01) +
F14	1.919e+01 (6.589e+00)	1.909e+01 (7.359e+00) ≈	2.486e+01 (2.307e+00) +	2.486e+01 (2.208e+00) +	2.471e+01 (2.385e+00) +	2.474e+01 (2.325e+00) +
F15	1.434e+01 (8.719e-01)	1.777e+01 (5.052e-01) +	2.185e+01 (2.609e+00) +	2.236e+01 (3.264e+00) +	2.151e+01 (2.689e+00) +	2.221e+01 (3.944e+00) +
F16	1.250e+02 (2.196e+00)	1.255e+02 (1.917e+00) ≈	2.433e+02 (1.166e+02) +	2.405e+02 (1.047e+02) +	2.476e+02 (1.024e+02) +	2.238e+02 (8.414e+01) +
F17	9.991e+01 (7.158e+01)	1.023e+02 (6.434e+01) ≈	1.766e+02 (7.450e+01) +	1.920e+02 (6.475e+01) +	1.962e+02 (8.208e+01) +	1.354e+02 (6.319e+01) +
F18	2.024e+01 (2.824e+00)	2.106e+01 (1.905e-01) +	2.537e+01 (2.763e+00) +	2.556e+01 (2.232e+00) +	2.578e+01 (2.025e+00) +	2.535e+01 (2.900e+00) +
F19	3.819e+00 (6.960e-01)	4.027e+00 (3.976e-01) ≈	1.243e+01 (2.605e+00) +	1.321e+01 (2.503e+00) +	1.254e+01 (2.351e+00) +	1.231e+01 (2.576e+00) +
F20	2.725e+01 (4.889e+00)	2.453e+01 (3.108e+00) -	7.994e+01 (3.658e+01) +	7.544e+01 (1.642e+01) +	6.633e+01 (1.240e+01) +	3.429e+01 (8.646e+00) +
F21	2.063e+02 (2.597e+00)	2.062e+02 (1.981e+00) ≈	2.142e+02 (3.065e+00) +	2.154e+02 (2.811e+00) +	2.157e+02 (3.230e+00) +	2.153e+02 (4.607e+00) +
F22	1.000e+02 (0.000e+00)	1.000e+02 (0.000e+00) ≈	1.189e+03 (1.583e+03) -	1.679e+03 (1.861e+03) -	1.265e+03 (1.618e+03) -	1.092e+03 (1.098e+03) -
F23	4.029e+02 (6.300e+00)	4.018e+02 (6.756e+00) ≈	4.328e+02 (6.719e+00) +	4.326e+02 (6.668e+00) +	4.331e+02 (6.389e+00) +	4.386e+02 (9.798e+00) +
F24	4.854e+02 (4.602e+00)	4.836e+02 (5.435e+00) ≈	5.097e+02 (3.765e+00) +	5.095e+02 (4.427e+00) +	5.096e+02 (3.368e+00) +	5.125e+02 (4.431e+00) +
F25	4.779e+02 (2.032e-01)	4.802e+02 (2.351e-03) +	4.784e+02 (1.023e+01) +	4.798e+02 (1.401e+01) +	4.804e+02 (1.445e+01) +	4.773e+02 (6.850e+00) +
F26	5.289e+02 (1.315e+02)	5.795e+02 (1.333e+02) +	1.148e+03 (7.148e+01) +	1.139e+03 (5.888e+01) +	1.138e+03 (5.849e+01) +	1.137e+03 (5.811e+01) +
F27	4.517e+02 (1.408e+01)	4.632e+02 (5.070e+00) +	4.831e+02 (8.813e+00) +	4.823e+02 (8.530e+00) +	4.820e+02 (8.485e+00) +	4.963e+02 (1.275e+01) +
F28	4.510e+02 (9.747e+00)	4.588e+02 (1.722e-13) +	4.619e+02 (1.954e+01) +	4.609e+02 (1.899e+01) +	4.687e+02 (2.313e+01) +	4.695e+02 (2.474e+01) +
F29	2.452e+02 (4.111e+00)	3.054e+02 (8.092e+00) +	2.755e+02 (1.182e+01) +	2.896e+02 (1.933e+01) +	2.750e+02 (1.413e+01) +	2.546e+02 (9.231e+00) +
F30	8.250e+02 (1.404e+02)	5.801e+05 (4.935e+03) +	2.087e+03 (2.376e+03) +	2.359e+03 (2.780e+03) +	2.868e+03 (5.583e+03) +	4.588e+03 (3.357e+03) +
+/-/≈	-	10/4/15	24/2/3	24/2/3	24/2/3	24/2/3

	RDE MEAN (STD DEV)	RSDE MEAN (STD DEV)	SMLDE MEAN (STD DEV)	EBOwithCMAR MEAN (STD DEV)	jSO MEAN (STD DEV)	LSHADE-SPACMA MEAN (STD DEV)
F1	0.000e+00 (0.000e+00) ≈	0.000e+00 (0.000e+00) ≈	0.000e+00 (0.000e+00) ≈	0.000e+00 (0.000e+00) ≈	0.000e+00 (0.000e+00) ≈	0.000e+00 (0.000e+00) ≈
F3	0.000e+00 (0.000e+00) ≈	0.000e+00 (0.000e+00) ≈	0.000e+00 (0.000e+00) ≈	0.000e+00 (0.000e+00) ≈	0.000e+00 (0.000e+00) ≈	0.000e+00 (0.000e+00) ≈
F4	5.175e+01 (4.173e+01) +	7.381e+01 (4.779e+01) +	6.485e+01 (5.305e+01) +	4.286e+01 (3.323e+01) +	5.621e+01 (4.876e+01) +	2.945e+01 (2.978e+01) +
F5	1.456e+01 (2.958e+00) +	1.518e+01 (3.256e+00) +	1.486e+01 (3.165e+00) +	7.585e+00 (2.422e+00) +	1.641e+01 (3.462e+00) +	5.989e+00 (2.024e+00) ≈
F6	3.519e-08 (1.412e-07) -	3.256e-08 (9.878e-08) -	5.100e-08 (1.690e-07) -	8.537e-08 (1.141e-07) -	1.093e-06 (2.626e-06) -	0.000e+00 (0.000e+00) -
F7	6.535e+01 (3.543e+00) +	6.603e+01 (3.885e+00) +	6.693e+01 (3.584e+00) +	5.788e+01 (1.529e+00) -	6.650e+01 (3.473e+00) +	5.697e+01 (1.093e+00) -
F8	1.472e+01 (3.037e+00) +	1.460e+01 (3.037e+00) +	1.538e+01 (3.226e+00) +	7.911e+00 (2.469e+00) +	1.696e+01 (3.135e+00) +	5.814e+00 (1.881e+00) ≈
F9	0.000e+00 (0.000e+00) ≈	0.000e+00 (0.000e+00) ≈	0.000e+00 (0.000e+00) ≈	0.000e+00 (0.000e+00) ≈	0.000e+00 (0.000e+00) ≈	0.000e+00 (0.000e+00) ≈
F10	3.240e+03 (3.793e+02) +	3.035e+03 (4.336e+02) +	3.098e+03 (3.559e+02) +	3.115e+03 (4.007e+02) +	3.140e+03 (3.672e+02) +	3.492e+03 (6.636e+02) +
F11	2.114e+01 (3.725e+00) +	1.847e+01 (3.040e+00) +	2.157e+01 (3.002e+00) +	2.636e+01 (3.363e+00) +	2.794e+01 (3.328e+00) +	3.222e+01 (4.752e+00) +
F12	1.650e+03 (4.851e+02) +	1.547e+03 (4.217e+02) +	1.965e+03 (4.966e+02) +	1.939e+03 (8.339e+02) +	1.681e+03 (5.229e+02) +	1.564e+03 (3.991e+02) +
F13	3.556e+01 (2.473e+01) +	3.379e+01 (2.604e+01) +	3.977e+01 (2.813e+01) +	4.140e+01 (2.477e+01) +	3.060e+01 (2.123e+01) +	3.713e+01 (2.323e+01) +
F14	2.651e+01 (1.851e+00) +	2.438e+01 (1.855e+00) +	2.457e+01 (1.825e+00) +	3.121e+01 (3.517e+00) +	2.496e+01 (1.873e+00) +	2.945e+01 (3.391e+00) +
F15	2.221e+01 (3.816e+00) +	1.909e+01 (2.373e+00) +	2.169e+01 (2.925e+00) +	2.936e+01 (5.196e+00) +	2.386e+01 (2.488e+00) +	3.040e+01 (5.627e+00) +
F16	2.677e+02 (1.234e+02) +	2.507e+02 (1.065e+02) +	2.338e+02 (1.162e+02) +	3.464e+02 (1.458e+02) +	4.505e+02 (1.378e+02) +	3.352e+02 (2.012e+02) +
F17	2.060e+02 (6.985e+01) +	2.101e+02 (6.683e+01) +	1.932e+02 (7.495e+01) +	2.748e+02 (8.635e+01) +	2.829e+02 (8.614e+01) +	2.768e+02 (9.489e+01) +
F18	2.490e+01 (2.188e+00) +	2.349e+01 (1.830e+00) +	2.467e+01 (1.826e+00) +	3.203e+01 (5.987e+00) +	2.428e+01 (2.017e+00) +	3.242e+01 (6.074e+00) +
F19	1.254e+01 (2.386e+00) +	1.089e+01 (2.371e+00) +	1.264e+01 (2.099e+00) +	2.448e+01 (3.936e+00) +	1.414e+01 (2.262e+00) +	2.171e+01 (3.205e+00) +
F20	7.414e+01 (3.806e+01) +	7.299e+01 (1.485e+01) +	7.684e+01 (3.344e+01) +	1.472e+02 (7.445e+01) +	1.401e+02 (7.738e+01) +	1.681e+02 (1.027e+02) +
F21	2.136e+02 (3.691e+00) +	2.143e+02 (3.300e+00) +	2.156e+02 (3.708e+00) +	2.106e+02 (4.057e+00) +	2.192e+02 (3.766e+00) +	2.151e+02 (9.314e+00) +
F22	1.614e+03 (1.789e+03) -	1.527e+03 (1.732e+03) -	8.781e+02 (1.511e+03) -	3.654e+02 (9.243e+02) -	1.487e+03 (1.753e+03) -	1.378e+03 (1.936e+03) -
F23	4.294e+02 (8.231e+00) +	4.363e+02 (6.822e+00) +	4.340e+02 (6.875e+00) +	4.340e+02 (8.162e+00) +	4.301e+02 (6.236e+00) +	4.409e+02 (6.451e+00) +
F24	5.054e+02 (4.497e+00) +	5.113e+02 (4.419e+00) +	5.097e+02 (3.492e+00) +	5.065e+02 (3.847e+00) +	5.075e+02 (4.127e+00) +	5.126e+02 (6.870e+00) +
F25	4.816e+02 (9.757e+00) +	4.794e+02 (9.193e+00) +	4.782e+02 (7.312e+00) +	4.886e+02 (2.468e+01) +	4.809e+02 (2.800e+00) +	4.807e+02 (2.319e+00) +
F26	1.095e+03 (5.532e+01) +	1.142e+03 (6.261e+01) +	1.137e+03 (5.958e+01) +	7.056e+02 (4.064e+02) ≈	1.129e+03 (5.617e+01) +	1.143e+03 (4.769e+01) +
F27	4.728e+02 (7.663e+00) +	4.808e+02 (9.200e+00) +	4.808e+02 (9.907e+00) +	5.224e+02 (7.755e+00) +	5.113e+02 (1.108e+01) +	5.317e+02 (1.476e+01) +
F28	4.536e+02 (9.126e+00) +	4.638e+02 (2.061e+01) +	4.668e+02 (2.227e+01) +	4.665e+02 (1.794e+01) +	4.598e+02 (6.840e+00) +	4.598e+02 (6.840e+00) +
F29	2.798e+02 (3.177e+01) +	2.842e+02 (2.455e+01) +	2.771e+02 (1.215e+01) +	3.473e+02 (1.969e+01) +	3.629e+02 (1.316e+01) +	3.920e+02 (7.151e+01) +
F30	2.031e+03 (1.845e+03) +	1.626e+03 (9.227e+02) +	2.106e+03 (2.018e+03) +	6.182e+05 (3.618e+04) +	6.011e+05 (2.986e+04) +	6.681e+05 (6.850e+04) +
+/-/≈	24/2/3	24/2/3	24/2/3	22/3/4	24/2/3	21/3/5

Entries are mean (standard deviation) objective values computed over independent runs. Symbols denote the outcome of two-sided Wilcoxon rank-sum tests using L-SRTDE-ALP as the reference: "+" indicates that L-SRTDE-ALP performed significantly better than the comparator; "-" indicates that L-SRTDE-ALP performed significantly worse; and "≈" indicates no statistically significant difference. The final row reports per-configuration counts of wins/losses/ties versus the reference (format: wins/losses/ties).

S4 Table. Best and median objective errors obtained by the compared algorithms on the 50-dimensional CEC 2017 test suite.

	L-SRTDE-ALP BEST / MEDIAN	L-SRTDE BEST / MEDIAN	BWDE BEST / MEDIAN	DPSDE BEST / MEDIAN	FODE BEST / MEDIAN	MSDE BEST / MEDIAN
F1	0.000e+00 / 0.000e+00	0.000e+00 / 0.000e+00	0.000e+00 / 0.000e+00	0.000e+00 / 0.000e+00	0.000e+00 / 0.000e+00	0.000e+00 / 0.000e+00
F3	0.000e+00 / 0.000e+00	0.000e+00 / 0.000e+00	0.000e+00 / 0.000e+00	0.000e+00 / 0.000e+00	0.000e+00 / 0.000e+00	0.000e+00 / 0.000e+00
F4	2.072e+01 / 2.080e+01	2.851e+01 / 2.851e+01	0.000e+00 / 2.208e+01	4.002e-07 / 2.208e+01	0.000e+00 / 2.217e+01	2.063e+01 / 2.117e+01
F5	2.985e+00 / 5.970e+00	1.990e+00 / 5.970e+00	7.985e+00 / 1.414e+01	7.998e+00 / 1.497e+01	9.573e+00 / 1.510e+01	6.965e+00 / 1.393e+01
F6	2.735e-06 / 1.695e-05	0.000e+00 / 1.919e-07	0.000e+00 / 0.000e+00	0.000e+00 / 0.000e+00	0.000e+00 / 0.000e+00	0.000e+00 / 0.000e+00
F7	5.525e+01 / 6.018e+01	5.588e+01 / 5.925e+01	5.966e+01 / 6.602e+01	6.007e+01 / 6.619e+01	5.975e+01 / 6.594e+01	5.755e+01 / 6.308e+01
F8	2.985e+00 / 5.970e+00	1.990e+00 / 5.970e+00	4.987e+00 / 1.493e+01	9.018e+00 / 1.444e+01	8.228e+00 / 1.418e+01	6.965e+00 / 1.492e+01
F9	0.000e+00 / 0.000e+00	0.000e+00 / 0.000e+00	0.000e+00 / 0.000e+00	0.000e+00 / 0.000e+00	0.000e+00 / 0.000e+00	0.000e+00 / 0.000e+00
F10	1.254e+02 / 4.287e+02	1.267e+02 / 3.056e+02	2.261e+03 / 3.048e+03	1.930e+03 / 3.375e+03	2.235e+03 / 3.050e+03	7.852e+02 / 2.073e+03
F11	1.008e+01 / 1.118e+01	1.826e+01 / 1.926e+01	1.434e+01 / 2.217e+01	1.411e+01 / 2.190e+01	1.328e+01 / 2.039e+01	1.349e+01 / 1.970e+01
F12	5.591e+00 / 1.341e+02	9.973e+00 / 1.345e+02	6.792e+02 / 1.867e+03	9.921e+02 / 1.794e+03	1.158e+03 / 1.931e+03	8.933e+02 / 1.889e+03
F13	4.780e-01 / 4.285e+00	5.393e-01 / 4.118e+00	7.734e+00 / 4.227e+01	5.443e+00 / 4.403e+01	5.867e+00 / 4.133e+01	4.335e+00 / 3.989e+01
F14	3.588e-02 / 2.105e+01	9.957e-01 / 2.155e+01	2.027e+01 / 2.436e+01	2.059e+01 / 2.460e+01	2.110e+01 / 2.410e+01	2.104e+01 / 2.405e+01
F15	1.308e+01 / 1.407e+01	1.717e+01 / 1.814e+01	1.795e+01 / 2.161e+01	1.662e+01 / 2.210e+01	1.667e+01 / 2.122e+01	1.606e+01 / 2.109e+01
F16	1.210e+02 / 1.250e+02	1.226e+02 / 1.245e+02	1.272e+02 / 2.509e+02	1.245e+02 / 2.502e+02	1.303e+02 / 2.511e+02	1.230e+02 / 2.451e+02
F17	2.419e+01 / 5.738e+01	2.312e+01 / 1.476e+02	6.049e+01 / 2.026e+02	6.329e+01 / 2.107e+02	5.686e+01 / 2.068e+02	2.968e+01 / 1.628e+02
F18	5.426e-01 / 2.055e+01	2.091e+01 / 2.104e+01	2.033e+01 / 2.525e+01	2.161e+01 / 2.545e+01	2.240e+01 / 2.552e+01	2.141e+01 / 2.440e+01
F19	2.350e+00 / 3.800e+00	3.265e+00 / 4.065e+00	7.103e+00 / 1.219e+01	8.602e+00 / 1.307e+01	7.057e+00 / 1.325e+01	5.785e+00 / 1.225e+01
F20	2.020e+01 / 2.565e+01	2.113e+01 / 2.373e+01	4.237e+01 / 7.102e+01	4.790e+01 / 7.299e+01	4.242e+01 / 6.459e+01	2.265e+01 / 3.397e+01
F21	2.015e+02 / 2.066e+02	2.016e+02 / 2.067e+02	2.085e+02 / 2.142e+02	2.097e+02 / 2.159e+02	2.090e+02 / 2.154e+02	2.060e+02 / 2.143e+02
F22	1.000e+02 / 1.000e+02	1.000e+02 / 1.000e+02	1.000e+02 / 1.000e+02	1.000e+02 / 1.000e+02	1.000e+02 / 1.000e+02	1.000e+02 / 1.000e+02
F23	3.870e+02 / 4.028e+02	3.879e+02 / 4.041e+02	4.155e+02 / 4.331e+02	4.190e+02 / 4.328e+02	4.187e+02 / 4.322e+02	4.195e+02 / 4.375e+02
F24	4.712e+02 / 4.867e+02	4.716e+02 / 4.854e+02	4.990e+02 / 5.100e+02	5.001e+02 / 5.091e+02	5.020e+02 / 5.093e+02	5.043e+02 / 5.119e+02
F25	4.774e+02 / 4.793e+02	4.802e+02 / 4.802e+02	4.528e+02 / 4.793e+02	4.417e+02 / 4.794e+02	4.489e+02 / 4.793e+02	4.315e+02 / 4.784e+02
F26	4.000e+02 / 5.647e+02	4.000e+02 / 6.161e+02	9.520e+02 / 1.146e+03	1.009e+03 / 1.143e+03	1.037e+03 / 1.139e+03	9.862e+02 / 1.132e+03
F27	4.386e+02 / 4.475e+02	4.588e+02 / 4.606e+02	4.666e+02 / 4.821e+02	4.691e+02 / 4.808e+02	4.645e+02 / 4.809e+02	4.514e+02 / 5.000e+02
F28	4.484e+02 / 4.491e+02	4.588e+02 / 4.588e+02	4.512e+02 / 4.518e+02	4.511e+02 / 4.517e+02	4.511e+02 / 4.519e+02	4.490e+02 / 4.502e+02
F29	2.378e+02 / 2.444e+02	2.925e+02 / 3.046e+02	2.496e+02 / 2.763e+02	2.528e+02 / 2.923e+02	2.536e+02 / 2.736e+02	2.363e+02 / 2.522e+02
F30	4.872e+02 / 8.086e+02	5.794e+05 / 5.794e+05	9.003e+02 / 1.372e+03	1.025e+03 / 1.320e+03	9.273e+02 / 1.210e+03	7.775e+02 / 4.243e+03
N_{med}	22	11	5	5	5	5
N_{best}	19	7	6	5	6	7

	RDE BEST / MEDIAN	RSDE BEST / MEDIAN	SMLDE BEST / MEDIAN	EBOwithCMAR BEST / MEDIAN	jSO BEST / MEDIAN	LSHADE-SPACMA BEST / MEDIAN
F1	0.000e+00 / 0.000e+00	0.000e+00 / 0.000e+00	0.000e+00 / 0.000e+00	0.000e+00 / 0.000e+00	0.000e+00 / 0.000e+00	0.000e+00 / 0.000e+00
F3	0.000e+00 / 0.000e+00	0.000e+00 / 0.000e+00	0.000e+00 / 0.000e+00	0.000e+00 / 0.000e+00	0.000e+00 / 0.000e+00	0.000e+00 / 0.000e+00
F4	0.000e+00 / 2.211e+01	6.948e+00 / 8.965e+01	2.127e+01 / 2.181e+01	0.000e+00 / 2.851e+01	1.318e-04 / 2.851e+01	1.757e-01 / 2.851e+01
F5	1.047e+01 / 1.395e+01	9.063e+00 / 1.470e+01	7.158e+00 / 1.505e+01	2.985e+00 / 6.965e+00	8.961e+00 / 1.620e+01	1.990e+00 / 5.970e+00
F6	0.000e+00 / 0.000e+00	0.000e+00 / 0.000e+00	0.000e+00 / 0.000e+00	0.000e+00 / 4.794e-08	0.000e+00 / 3.107e-07	0.000e+00 / 0.000e+00
F7	5.838e+01 / 6.482e+01	5.664e+01 / 6.514e+01	6.034e+01 / 6.654e+01	5.528e+01 / 5.788e+01	5.752e+01 / 6.664e+01	5.457e+01 / 5.695e+01
F8	7.960e+00 / 1.496e+01	9.795e+00 / 1.443e+01	7.054e+00 / 1.524e+01	3.980e+00 / 7.960e+00	9.951e+00 / 1.697e+01	9.950e-01 / 5.970e+00
F9	0.000e+00 / 0.000e+00	0.000e+00 / 0.000e+00	0.000e+00 / 0.000e+00	0.000e+00 / 0.000e+00	0.000e+00 / 0.000e+00	0.000e+00 / 0.000e+00
F10	2.268e+03 / 3.281e+03	1.706e+03 / 3.016e+03	2.098e+03 / 3.156e+03	2.141e+03 / 3.160e+03	2.405e+03 / 3.232e+03	1.651e+03 / 3.466e+03
F11	1.348e+01 / 2.061e+01	1.244e+01 / 1.922e+01	1.533e+01 / 2.226e+01	1.926e+01 / 2.622e+01	2.125e+01 / 2.852e+01	2.423e+01 / 3.313e+01
F12	7.236e+02 / 1.590e+03	7.575e+02 / 1.517e+03	9.259e+02 / 1.918e+03	1.229e+03 / 1.784e+03	7.519e+02 / 1.653e+03	8.159e+02 / 1.525e+03
F13	7.782e+00 / 4.078e+01	6.976e+00 / 3.497e+01	7.170e+00 / 4.189e+01	7.960e+00 / 4.492e+01	4.585e+00 / 3.726e+01	2.985e+00 / 3.996e+01
F14	2.311e+01 / 2.668e+01	2.011e+01 / 2.437e+01	2.207e+01 / 2.435e+01	2.500e+01 / 3.095e+01	2.030e+01 / 2.435e+01	2.398e+01 / 2.896e+01
F15	1.583e+01 / 2.138e+01	1.501e+01 / 1.873e+01	1.730e+01 / 2.119e+01	9.588e+00 / 2.934e+01	1.888e+01 / 2.342e+01	2.242e+01 / 2.916e+01
F16	1.272e+02 / 2.579e+02	1.238e+02 / 2.502e+02	1.261e+02 / 2.444e+02	1.255e+02 / 3.435e+02	1.347e+02 / 4.772e+02	1.263e+02 / 3.023e+02
F17	6.967e+01 / 2.113e+02	5.645e+01 / 2.115e+02	5.782e+01 / 2.132e+02	6.277e+01 / 2.609e+02	8.543e+01 / 2.599e+02	7.999e+01 / 2.296e+02
F18	2.079e+01 / 2.459e+01	2.040e+01 / 2.294e+01	2.138e+01 / 2.431e+01	2.248e+01 / 3.047e+01	2.053e+01 / 2.387e+01	2.341e+01 / 3.128e+01
F19	7.268e+00 / 1.218e+01	6.844e+00 / 1.118e+01	9.378e+00 / 1.274e+01	1.705e+01 / 2.443e+01	1.039e+01 / 1.386e+01	1.412e+01 / 2.173e+01
F20	4.202e+01 / 6.562e+01	4.949e+01 / 7.233e+01	4.663e+01 / 7.468e+01	5.757e+01 / 1.205e+02	5.119e+01 / 1.133e+02	7.725e+01 / 1.115e+02
F21	2.077e+02 / 2.136e+02	2.083e+02 / 2.140e+02	2.054e+02 / 2.161e+02	2.016e+02 / 2.103e+02	2.117e+02 / 2.192e+02	2.028e+02 / 2.105e+02
F22	1.000e+02 / 1.000e+02	1.000e+02 / 1.000e+02	1.000e+02 / 1.000e+02	1.000e+02 / 1.000e+02	1.000e+02 / 1.000e+02	1.000e+02 / 1.000e+02
F23	4.142e+02 / 4.304e+02	4.216e+02 / 4.361e+02	4.203e+02 / 4.328e+02	4.148e+02 / 4.344e+02	4.101e+02 / 4.298e+02	4.237e+02 / 4.417e+02
F24	4.962e+02 / 5.052e+02	5.029e+02 / 5.117e+02	5.031e+02 / 5.098e+02	4.989e+02 / 5.059e+02	4.996e+02 / 5.073e+02	5.022e+02 / 5.111e+02
F25	4.552e+02 / 4.793e+02	4.505e+02 / 4.793e+02	4.393e+02 / 4.792e+02	4.802e+02 / 4.803e+02	4.773e+02 / 4.802e+02	4.774e+02 / 4.802e+02
F26	9.807e+02 / 1.099e+03	1.026e+03 / 1.134e+03	9.792e+02 / 1.145e+03	3.000e+02 / 9.061e+02	9.663e+02 / 1.133e+03	1.046e+03 / 1.148e+03
F27	4.567e+02 / 4.726e+02	4.663e+02 / 4.797e+02	4.666e+02 / 4.792e+02	5.033e+02 / 5.231e+02	4.770e+02 / 5.125e+02	5.064e+02 / 5.288e+02
F28	4.510e+02 / 4.518e+02	4.510e+02 / 4.520e+02	4.510e+02 / 4.516e+02	4.588e+02 / 4.588e+02	4.588e+02 / 4.588e+02	4.588e+02 / 4.588e+02
F29	2.371e+02 / 2.686e+02	2.485e+02 / 2.773e+02	2.544e+02 / 2.782e+02	3.093e+02 / 3.453e+02	3.287e+02 / 3.636e+02	3.363e+02 / 3.718e+02
F30	9.770e+02 / 1.310e+03	9.988e+02 / 1.277e+03	9.796e+02 / 1.362e+03	5.794e+05 / 6.144e+05	5.794e+05 / 5.905e+05	5.794e+05 / 6.620e+05
N_{med}	5	5	5	4	4	6
N_{best}	6	5	5	8	5	7

Entries are best / median objective errors computed over 51 independent runs. Lower values indicate better performance. Best denotes the minimum error observed across the 51 runs. N_{med} and N_{best} denote the numbers of functions for which each algorithm attained the lowest median and lowest best value, respectively. Ties are counted for all tied algorithms.

S5 Table. Comparison of L-SRTDE-ALP with competing methods on the CEC 2017 test suite (100-dimensional). Results are based on Wilcoxon rank-sum tests at the significance level $\alpha = 0.05$.

	L-SRTDE-ALP MEAN (STD DEV)	L-SRTDE MEAN (STD DEV)	BWDE MEAN (STD DEV)	DPSDE MEAN (STD DEV)	FODE MEAN (STD DEV)	MSDE MEAN (STD DEV)
F1	2.397e+03 (2.196e+03)	6.397e+01 (1.183e+02) -	0.000e+00 (0.000e+00) -	0.000e+00 (0.000e+00) -	0.000e+00 (0.000e+00) -	0.000e+00 (0.000e+00) -
F3	5.059e-02 (3.885e-02)	4.604e-03 (4.783e-03) -	2.936e-06 (2.965e-06) -	6.655e-06 (5.387e-06) -	3.967e-06 (5.724e-06) -	3.276e-06 (4.504e-06) -
F4	1.862e+02 (2.069e+01)	2.036e+02 (9.029e+00) +	1.801e+02 (4.614e+01) ≈	1.690e+02 (4.761e+01) ≈	1.880e+02 (3.096e+01) +	1.818e+02 (4.301e+01) +
F5	1.732e+01 (3.271e+00)	1.707e+01 (3.338e+00) ≈	3.998e+01 (1.080e+01) +	4.006e+01 (9.774e+00) +	3.958e+01 (9.965e+00) +	3.012e+01 (4.838e+00) +
F6	3.290e-04 (6.194e-05)	4.557e-05 (1.900e-05) -	9.962e-04 (2.015e-03) -	7.898e-04 (1.451e-03) -	4.644e-04 (9.343e-04) -	8.604e-04 (1.440e-03) ≈
F7	1.167e+02 (5.076e+00)	1.159e+02 (4.583e+00) ≈	1.570e+02 (1.097e+01) +	1.645e+02 (1.371e+01) +	1.591e+02 (1.145e+01) +	1.283e+02 (5.234e+00) +
F8	1.664e+01 (2.864e+00)	1.615e+01 (2.725e+00) ≈	3.560e+01 (8.681e+00) +	3.809e+01 (1.112e+01) +	3.754e+01 (9.101e+00) +	2.999e+01 (6.713e+00) +
F9	0.000e+00 (0.000e+00)	0.000e+00 (0.000e+00) ≈	0.000e+00 (0.000e+00) ≈	0.000e+00 (0.000e+00) ≈	0.000e+00 (0.000e+00) ≈	0.000e+00 (0.000e+00) ≈
F10	1.577e+03 (5.879e+02)	1.500e+03 (5.399e+02) ≈	1.089e+04 (8.386e+02) +	1.155e+04 (8.851e+02) +	1.066e+04 (8.902e+02) +	6.642e+03 (2.055e+03) +
F11	2.984e+01 (2.474e+01)	3.053e+01 (2.536e+01) ≈	1.086e+02 (3.101e+01) +	1.419e+02 (4.365e+01) +	1.241e+02 (4.024e+01) +	1.523e+02 (5.400e+01) +
F12	2.435e+03 (1.399e+03)	2.345e+03 (1.382e+03) ≈	2.135e+04 (1.046e+04) +	2.039e+04 (8.942e+03) +	1.689e+04 (7.859e+03) +	1.794e+04 (1.064e+04) +
F13	4.370e+01 (1.876e+01)	5.107e+01 (1.578e+01) +	1.157e+02 (2.976e+01) +	1.513e+02 (5.063e+01) +	1.141e+02 (3.224e+01) +	1.167e+02 (3.338e+01) +
F14	2.572e+01 (1.856e+00)	2.702e+01 (1.931e+00) +	6.258e+01 (1.036e+01) +	7.754e+01 (1.603e+01) +	6.113e+01 (1.013e+01) +	6.716e+01 (1.072e+01) +
F15	5.644e+01 (3.075e+01)	6.736e+01 (2.934e+01) +	1.979e+02 (5.153e+01) +	2.172e+02 (5.152e+01) +	1.843e+02 (4.349e+01) +	1.859e+02 (4.752e+01) +
F16	1.131e+02 (6.851e+01)	9.967e+01 (7.011e+01) ≈	1.248e+03 (3.276e+02) +	1.135e+03 (2.581e+02) +	1.169e+03 (3.215e+02) +	4.967e+02 (2.653e+02) +
F17	9.062e+01 (7.340e+01)	8.890e+01 (6.058e+01) ≈	9.451e+02 (2.179e+02) +	9.477e+02 (2.447e+02) +	9.846e+02 (2.476e+02) +	3.157e+02 (1.720e+02) +
F18	2.541e+01 (1.612e+00)	2.612e+01 (1.722e+00) +	2.232e+02 (4.858e+01) +	2.261e+02 (4.598e+01) +	2.151e+02 (5.190e+01) +	2.033e+02 (4.328e+01) +
F19	1.514e+01 (2.471e+00)	1.590e+01 (2.309e+00) ≈	1.291e+02 (2.302e+01) +	1.584e+02 (2.278e+01) +	1.344e+02 (2.635e+01) +	1.274e+02 (2.069e+01) +
F20	1.796e+02 (4.692e+01)	1.728e+02 (2.355e+01) ≈	1.117e+03 (2.320e+02) +	1.164e+03 (1.681e+02) +	1.081e+03 (2.015e+02) +	3.884e+02 (1.506e+02) +
F21	2.368e+02 (3.468e+00)	2.381e+02 (4.103e+00) ≈	2.585e+02 (9.035e+00) +	2.581e+02 (7.747e+00) +	2.590e+02 (9.806e+00) +	2.553e+02 (6.568e+00) +
F22	2.262e+03 (1.093e+03)	2.100e+03 (5.700e+02) -	1.099e+04 (9.489e+02) +	1.218e+04 (1.191e+03) +	1.101e+04 (1.098e+03) +	5.560e+03 (1.186e+03) +
F23	5.371e+02 (7.703e+00)	5.400e+02 (7.144e+00) ≈	5.641e+02 (9.687e+00) +	5.617e+02 (9.353e+00) +	5.612e+02 (8.481e+00) +	5.614e+02 (9.345e+00) +
F24	8.087e+02 (1.310e+01)	8.142e+02 (1.563e+01) +	9.101e+02 (8.386e+00) +	9.099e+02 (8.747e+00) +	9.058e+02 (7.317e+00) +	9.112e+02 (7.034e+00) +
F25	6.178e+02 (3.151e+01)	6.403e+02 (2.874e+01) +	7.333e+02 (2.958e+01) +	7.293e+02 (3.084e+01) +	7.353e+02 (2.695e+01) +	7.209e+02 (3.395e+01) +
F26	2.244e+03 (1.884e+02)	2.185e+03 (1.623e+02) ≈	3.269e+03 (1.148e+02) +	3.315e+03 (1.131e+02) +	3.290e+03 (1.017e+02) +	3.284e+03 (1.026e+02) +
F27	5.000e+02 (7.095e-04)	5.227e+02 (1.152e+01) +	5.000e+02 (2.637e-04) +	5.000e+02 (2.978e-04) +	5.000e+02 (3.412e-04) +	5.000e+02 (1.132e-03) +
F28	5.000e+02 (6.607e-04)	4.980e+02 (1.312e+01) -	4.992e+02 (2.860e+00) +	4.985e+02 (4.875e+00) +	4.981e+02 (6.070e+00) +	5.000e+02 (9.242e-04) ≈
F29	5.527e+02 (3.466e+01)	6.926e+02 (3.902e+01) +	8.687e+02 (1.384e+02) +	9.043e+02 (1.984e+02) +	9.065e+02 (1.809e+02) +	7.466e+02 (1.869e+02) +
F30	5.902e+02 (6.770e+01)	2.065e+03 (1.632e+01) +	1.109e+03 (2.106e+02) +	1.158e+03 (2.058e+02) +	1.102e+03 (1.848e+02) +	6.659e+02 (2.249e+02) ≈
+/-/≈	-	10/5/14	24/3/2	24/3/2	25/3/1	23/2/4

	RDE MEAN (STD DEV)	RSDE MEAN (STD DEV)	SMLDE MEAN (STD DEV)	EBOwithCMAR MEAN (STD DEV)	jSO MEAN (STD DEV)	LSHADE-SPACMA MEAN (STD DEV)
F1	0.000e+00 (0.000e+00) -	0.000e+00 (0.000e+00) -	3.807e-09 (2.719e-08) -	1.329e-09 (7.518e-09) -	0.000e+00 (0.000e+00) -	0.000e+00 (0.000e+00) -
F3	1.006e-05 (1.269e-05) -	7.643e-04 (9.429e-04) -	6.747e-06 (6.392e-06) -	2.991e-07 (7.042e-07) -	2.391e-06 (2.725e-06) -	0.000e+00 (0.000e+00) -
F4	1.602e+02 (4.657e+01) -	1.848e+02 (3.669e+01) ≈	1.833e+02 (3.787e+01) +	1.934e+02 (3.090e+01) +	1.896e+02 (2.892e+01) ≈	2.012e+02 (1.109e+01) +
F5	4.105e+01 (8.988e+00) +	3.876e+01 (9.590e+00) +	4.121e+01 (1.146e+01) +	2.866e+01 (5.276e+00) +	4.391e+01 (5.607e+00) +	1.217e+01 (2.989e+00) -
F6	9.087e-04 (1.604e-03) ≈	7.947e-04 (1.365e-03) -	5.394e-04 (1.194e-03) -	1.629e-05 (7.133e-06) -	2.024e-04 (6.199e-04) -	0.000e+00 (0.000e+00) -
F7	1.577e+02 (8.858e+00) +	1.576e+02 (1.154e+01) +	1.631e+02 (1.379e+01) +	1.222e+02 (4.467e+00) +	1.449e+02 (6.703e+00) +	1.118e+02 (1.545e+00) -
F8	4.085e+01 (1.005e+01) +	3.866e+01 (9.710e+00) +	3.827e+01 (1.060e+01) +	2.967e+01 (7.477e+00) +	4.215e+01 (5.522e+00) +	1.016e+01 (2.980e+00) -
F9	0.000e+00 (0.000e+00) ≈	0.000e+00 (0.000e+00) ≈	0.000e+00 (0.000e+00) ≈	1.755e-03 (1.254e-02) ≈	4.590e-02 (1.149e-01) -	0.000e+00 (0.000e+00) ≈
F10	1.044e+04 (8.224e+02) +	1.053e+04 (9.015e+02) +	1.086e+04 (8.305e+02) +	9.905e+03 (1.912e+03) +	9.704e+03 (6.816e+02) +	1.002e+04 (9.088e+02) +
F11	1.193e+02 (4.206e+01) +	8.212e+01 (3.558e+01) +	1.114e+02 (3.884e+01) +	6.558e+01 (2.001e+01) +	1.132e+02 (4.320e+01) +	5.199e+01 (2.767e+01) +
F12	1.544e+04 (6.824e+03) +	2.004e+04 (8.195e+03) +	1.899e+04 (1.067e+04) +	4.185e+03 (7.894e+02) +	1.843e+04 (8.350e+03) +	4.800e+03 (7.472e+02) +
F13	1.155e+02 (3.071e+01) +	1.255e+02 (3.768e+01) +	1.189e+02 (3.305e+01) +	2.447e+02 (8.838e+01) +	1.449e+02 (3.801e+01) +	1.481e+02 (4.098e+01) +
F14	6.672e+01 (1.120e+01) +	4.537e+01 (5.376e+00) +	6.343e+01 (1.104e+01) +	1.384e+02 (2.962e+01) +	6.434e+01 (1.089e+01) +	7.137e+01 (1.235e+01) +
F15	1.814e+02 (3.963e+01) +	1.199e+02 (3.285e+01) +	1.974e+02 (5.049e+01) +	1.654e+02 (3.867e+01) +	1.621e+02 (3.805e+01) +	1.075e+02 (3.111e+01) +
F16	1.516e+03 (3.086e+02) +	1.112e+03 (3.648e+02) +	1.118e+03 (2.869e+02) +	1.414e+03 (3.765e+02) +	1.859e+03 (3.486e+02) +	1.248e+03 (4.720e+02) +
F17	1.007e+03 (2.416e+02) +	9.515e+02 (2.147e+02) +	9.284e+02 (2.467e+02) +	1.214e+03 (2.569e+02) +	1.278e+03 (2.384e+02) +	1.031e+03 (2.981e+02) +
F18	2.137e+02 (4.020e+01) +	1.643e+02 (4.000e+01) +	2.087e+02 (4.655e+01) +	2.371e+02 (5.939e+01) +	1.673e+02 (3.650e+01) +	1.349e+02 (3.641e+01) +
F19	1.244e+02 (2.088e+01) +	5.994e+01 (8.244e+00) +	1.336e+02 (2.154e+01) +	1.153e+02 (1.878e+01) +	1.049e+02 (2.008e+01) +	7.350e+01 (1.095e+01) +
F20	1.179e+03 (1.963e+02) +	1.046e+03 (1.973e+02) +	1.121e+03 (2.415e+02) +	1.362e+03 (3.093e+02) +	1.376e+03 (2.428e+02) +	1.468e+03 (3.190e+02) +
F21	2.575e+02 (6.450e+00) +	2.597e+02 (8.031e+00) +	2.565e+02 (8.386e+00) +	2.595e+02 (1.057e+01) +	2.638e+02 (6.429e+00) +	2.438e+02 (1.440e+01) +
F22	1.160e+04 (7.586e+02) +	1.122e+04 (6.738e+02) +	1.126e+04 (1.086e+03) +	1.019e+04 (2.699e+03) +	1.021e+04 (2.182e+03) +	9.985e+03 (1.084e+03) +
F23	5.634e+02 (9.898e+00) +	5.656e+02 (7.174e+00) +	5.630e+02 (8.903e+00) +	5.774e+02 (1.314e+01) +	5.712e+02 (1.070e+01) +	5.807e+02 (7.952e+00) +
F24	8.860e+02 (1.120e+01) +	9.137e+02 (8.225e+00) +	9.077e+02 (8.383e+00) +	9.185e+02 (1.322e+01) +	9.022e+02 (7.893e+00) +	9.187e+02 (1.880e+01) +
F25	7.186e+02 (4.060e+01) +	7.281e+02 (3.156e+01) +	7.286e+02 (3.560e+01) +	7.161e+02 (3.705e+01) +	7.361e+02 (3.530e+01) +	7.086e+02 (3.773e+01) +
F26	3.128e+03 (8.657e+01) +	3.295e+03 (9.433e+01) +	3.281e+03 (1.006e+02) +	2.775e+03 (1.085e+03) +	3.267e+03 (8.019e+01) +	3.137e+03 (8.387e+01) +
F27	5.000e+02 (3.952e-04) +	5.000e+02 (2.866e-04) +	5.000e+02 (2.885e-04) +	5.881e+02 (1.527e+01) +	5.855e+02 (2.167e+01) +	5.932e+02 (1.537e+01) +
F28	4.994e+02 (2.666e+00) +	4.973e+02 (7.153e+00) +	4.989e+02 (4.701e+00) +	5.098e+02 (6.009e+01) +	5.268e+02 (2.731e+01) +	5.168e+02 (2.163e+01) +
F29	8.457e+02 (1.720e+02) +	9.273e+02 (1.828e+02) +	9.194e+02 (1.843e+02) +	1.279e+03 (2.421e+02) +	1.257e+03 (1.913e+02) +	1.587e+03 (3.342e+02) +
F30	1.047e+03 (2.179e+02) +	1.077e+03 (1.906e+02) +	9.742e+02 (1.624e+02) +	2.397e+03 (1.511e+02) +	2.325e+03 (1.188e+02) +	2.382e+03 (1.748e+02) +
+/-/≈	24/3/2	24/3/2	25/3/1	25/3/1	24/4/1	22/6/1

Entries are mean (standard deviation) objective values computed over independent runs. Symbols denote the outcome of two-sided Wilcoxon rank-sum tests using L-SRTDE-ALP as the reference: "+" indicates that L-SRTDE-ALP performed significantly better than the comparator; "-" indicates that L-SRTDE-ALP performed significantly worse; and "≈" indicates no statistically significant difference. The final row reports per-configuration counts of wins/losses/ties versus the reference (format: wins/losses/ties).

S6 Table. Best and median objective errors obtained by the compared algorithms on the 100-dimensional CEC 2017 test suite.

	L-SRTDE-ALP<br>BEST / MEDIAN	L-SRTDE<br>BEST / MEDIAN	BWDE<br>BEST / MEDIAN	DPSDE<br>BEST / MEDIAN	FODE<br>BEST / MEDIAN	MSDE<br>BEST / MEDIAN
F1	1.068e+01 / 1.710e+03	1.679e-01 / 2.238e+01	0.000e+00 / 0.000e+00	0.000e+00 / 0.000e+00	0.000e+00 / 0.000e+00	0.000e+00 / 0.000e+00
F3	1.386e-02 / 4.334e-02	2.322e-04 / 2.513e-03	1.261e-07 / 1.920e-06	9.726e-07 / 5.665e-06	2.736e-07 / 2.143e-06	1.036e-07 / 1.866e-06
F4	1.407e+02 / 1.961e+02	1.879e+02 / 2.058e+02	6.251e-01 / 2.030e+02	3.213e-01 / 1.552e+02	1.192e+02 / 2.087e+02	6.945e+01 / 2.033e+02
F5	1.094e+01 / 1.691e+01	1.094e+01 / 1.691e+01	1.791e+01 / 3.975e+01	2.288e+01 / 3.904e+01	2.189e+01 / 3.879e+01	1.890e+01 / 2.985e+01
F6	2.394e-04 / 3.162e-04	1.746e-05 / 4.365e-05	1.486e-07 / 6.396e-06	4.949e-07 / 6.407e-06	1.144e-06 / 5.731e-06	5.469e-07 / 8.187e-06
F7	1.090e+02 / 1.155e+02	1.097e+02 / 1.158e+02	1.370e+02 / 1.575e+02	1.342e+02 / 1.609e+02	1.315e+02 / 1.591e+02	1.178e+02 / 1.279e+02
F8	1.094e+01 / 1.691e+01	1.293e+01 / 1.592e+01	1.990e+01 / 3.716e+01	1.890e+01 / 3.681e+01	1.990e+01 / 3.838e+01	1.791e+01 / 2.885e+01
F9	0.000e+00 / 0.000e+00	0.000e+00 / 0.000e+00	0.000e+00 / 0.000e+00	0.000e+00 / 0.000e+00	0.000e+00 / 0.000e+00	0.000e+00 / 0.000e+00
F10	4.701e+02 / 1.400e+03	7.655e+02 / 1.459e+03	8.836e+03 / 1.095e+04	9.996e+03 / 1.160e+04	7.573e+03 / 1.079e+04	2.709e+03 / 5.983e+03
F11	1.175e+01 / 1.942e+01	7.976e+00 / 1.974e+01	5.976e+01 / 1.031e+02	6.234e+01 / 1.373e+02	4.339e+01 / 1.224e+02	6.721e+01 / 1.483e+02
F12	7.465e+02 / 2.041e+03	8.586e+02 / 2.004e+03	7.629e+03 / 1.909e+04	7.024e+03 / 1.916e+04	5.849e+03 / 1.436e+04	5.662e+03 / 1.526e+04
F13	2.192e+01 / 4.076e+01	1.531e+01 / 5.160e+01	5.257e+01 / 1.109e+02	5.306e+01 / 1.440e+02	6.329e+01 / 1.098e+02	6.267e+01 / 1.114e+02
F14	2.132e+01 / 2.606e+01	2.287e+01 / 2.731e+01	4.304e+01 / 6.181e+01	5.218e+01 / 7.577e+01	4.202e+01 / 6.099e+01	4.394e+01 / 6.579e+01
F15	1.523e+01 / 7.785e+01	1.685e+01 / 8.449e+01	1.049e+02 / 1.977e+02	1.249e+02 / 2.126e+02	9.877e+01 / 1.827e+02	1.166e+02 / 1.814e+02
F16	1.031e+01 / 1.332e+02	1.213e+01 / 1.360e+02	5.212e+02 / 1.247e+03	6.771e+02 / 1.106e+03	5.026e+02 / 1.197e+03	1.383e+02 / 4.348e+02
F17	3.383e+01 / 4.667e+01	3.497e+01 / 4.486e+01	5.427e+02 / 9.372e+02	3.635e+02 / 9.691e+02	3.300e+02 / 9.837e+02	9.943e+01 / 2.750e+02
F18	2.219e+01 / 2.538e+01	2.289e+01 / 2.593e+01	1.392e+02 / 2.120e+02	1.161e+02 / 2.258e+02	1.150e+02 / 2.156e+02	1.286e+02 / 1.917e+02
F19	1.088e+01 / 1.483e+01	1.218e+01 / 1.550e+01	8.851e+01 / 1.278e+02	1.029e+02 / 1.584e+02	7.255e+01 / 1.292e+02	7.679e+01 / 1.323e+02
F20	1.561e+02 / 1.677e+02	1.561e+02 / 1.684e+02	6.457e+02 / 1.142e+03	6.112e+02 / 1.180e+03	5.767e+02 / 1.089e+03	1.236e+02 / 3.251e+02
F21	2.309e+02 / 2.366e+02	2.297e+02 / 2.373e+02	2.448e+02 / 2.572e+02	2.465e+02 / 2.560e+02	2.413e+02 / 2.585e+02	2.420e+02 / 2.554e+02
F22	1.000e+02 / 2.440e+03	1.000e+02 / 1.998e+03	9.170e+03 / 1.087e+04	9.597e+03 / 1.206e+04	8.347e+03 / 1.117e+04	3.410e+03 / 5.376e+03
F23	5.209e+02 / 5.384e+02	5.240e+02 / 5.380e+02	5.462e+02 / 5.633e+02	5.392e+02 / 5.630e+02	5.410e+02 / 5.598e+02	5.358e+02 / 5.613e+02
F24	7.854e+02 / 8.052e+02	7.837e+02 / 8.175e+02	8.895e+02 / 9.100e+02	8.921e+02 / 9.098e+02	8.904e+02 / 9.051e+02	8.968e+02 / 9.116e+02
F25	5.665e+02 / 6.219e+02	5.770e+02 / 6.371e+02	6.317e+02 / 7.444e+02	6.528e+02 / 7.437e+02	6.826e+02 / 7.441e+02	6.205e+02 / 7.378e+02
F26	1.741e+03 / 2.255e+03	1.735e+03 / 2.146e+03	3.004e+03 / 3.278e+03	3.081e+03 / 3.308e+03	3.105e+03 / 3.279e+03	2.922e+03 / 3.292e+03
F27	5.000e+02 / 5.000e+02	5.024e+02 / 5.224e+02	5.000e+02 / 5.000e+02	5.000e+02 / 5.000e+02	5.000e+02 / 5.000e+02	5.000e+02 / 5.000e+02
F28	5.000e+02 / 5.000e+02	4.782e+02 / 4.933e+02	4.847e+02 / 5.000e+02	4.766e+02 / 5.000e+02	4.763e+02 / 5.000e+02	5.000e+02 / 5.000e+02
F29	4.331e+02 / 5.532e+02	5.737e+02 / 7.035e+02	6.147e+02 / 8.828e+02	5.212e+02 / 8.765e+02	6.700e+02 / 8.747e+02	5.692e+02 / 6.924e+02
F30	4.766e+02 / 5.811e+02	2.029e+03 / 2.066e+03	7.190e+02 / 1.100e+03	8.057e+02 / 1.145e+03	7.238e+02 / 1.114e+03	4.601e+02 / 5.849e+02
N_{med}	16	7	2	3	2	2
N_{best}	13	6	2	2	2	4

	RDE<br>BEST / MEDIAN	RSDE<br>BEST / MEDIAN	SMLDE<br>BEST / MEDIAN	EBOwithCMAR<br>BEST / MEDIAN	jSO<br>BEST / MEDIAN	LSHADE-SPACMA<br>BEST / MEDIAN
F1	0.000e+00 / 0.000e+00	0.000e+00 / 0.000e+00	0.000e+00 / 0.000e+00	0.000e+00 / 0.000e+00	0.000e+00 / 0.000e+00	0.000e+00 / 0.000e+00
F3	7.047e-07 / 5.507e-06	7.196e-05 / 4.695e-04	4.612e-07 / 4.707e-06	7.614e-08 / 1.859e-07	6.449e-08 / 1.448e-06	0.000e+00 / 0.000e+00
F4	4.265e+00 / 1.684e+02	8.242e+01 / 1.926e+02	7.502e+01 / 2.005e+02	0.000e+00 / 1.974e+02	8.452e+01 / 1.954e+02	1.692e+02 / 1.974e+02
F5	2.686e+01 / 3.980e+01	2.089e+01 / 3.910e+01	1.691e+01 / 4.043e+01	1.791e+01 / 2.786e+01	3.010e+01 / 4.405e+01	6.965e+00 / 1.194e+01
F6	9.145e-07 / 1.636e-05	3.000e-07 / 4.592e-06	2.085e-06 / 1.033e-05	5.126e-06 / 1.471e-05	7.673e-06 / 3.572e-05	0.000e+00 / 0.000e+00
F7	1.329e+02 / 1.580e+02	1.398e+02 / 1.563e+02	1.413e+02 / 1.610e+02	1.156e+02 / 1.215e+02	1.288e+02 / 1.442e+02	1.086e+02 / 1.117e+02
F8	2.388e+01 / 3.919e+01	2.288e+01 / 3.881e+01	1.890e+01 / 3.741e+01	1.791e+01 / 2.786e+01	2.726e+01 / 4.225e+01	3.980e+00 / 9.950e+00
F9	0.000e+00 / 0.000e+00	0.000e+00 / 0.000e+00	0.000e+00 / 0.000e+00	0.000e+00 / 0.000e+00	0.000e+00 / 0.000e+00	0.000e+00 / 0.000e+00
F10	8.327e+03 / 1.054e+04	8.121e+03 / 1.063e+04	8.873e+03 / 1.082e+04	6.770e+03 / 9.499e+03	7.541e+03 / 9.751e+03	7.915e+03 / 9.969e+03
F11	5.731e+01 / 1.051e+02	3.953e+01 / 6.900e+01	6.233e+01 / 1.001e+02	3.383e+01 / 6.146e+01	4.272e+01 / 1.042e+02	2.154e+01 / 4.255e+01
F12	6.301e+03 / 1.417e+04	6.670e+03 / 2.047e+04	6.951e+03 / 1.697e+04	2.452e+03 / 4.129e+03	6.174e+03 / 1.703e+04	3.265e+03 / 4.778e+03
F13	6.766e+01 / 1.131e+02	4.828e+01 / 1.192e+02	6.478e+01 / 1.139e+02	1.194e+02 / 2.135e+02	5.327e+01 / 1.396e+02	6.867e+01 / 1.419e+02
F14	4.732e+01 / 6.504e+01	3.555e+01 / 4.574e+01	3.816e+01 / 6.183e+01	8.169e+01 / 1.364e+02	4.407e+01 / 6.385e+01	4.686e+01 / 6.975e+01
F15	1.145e+02 / 1.810e+02	7.138e+01 / 1.091e+02	8.812e+01 / 1.881e+02	8.599e+01 / 1.625e+02	8.669e+01 / 1.647e+02	5.499e+01 / 1.063e+02
F16	5.190e+02 / 1.536e+03	2.928e+02 / 1.099e+03	6.626e+02 / 1.125e+03	7.859e+02 / 1.362e+03	1.031e+03 / 1.882e+03	4.796e+02 / 1.220e+03
F17	5.490e+02 / 9.839e+02	5.859e+02 / 9.309e+02	3.832e+02 / 9.608e+02	5.350e+02 / 1.182e+03	6.748e+02 / 1.273e+03	2.774e+02 / 1.082e+03
F18	1.153e+02 / 2.208e+02	1.068e+02 / 1.588e+02	8.432e+01 / 2.152e+02	1.080e+02 / 2.314e+02	1.014e+02 / 1.569e+02	8.204e+01 / 1.339e+02
F19	8.468e+01 / 1.231e+02	4.404e+01 / 5.930e+01	8.489e+01 / 1.315e+02	8.158e+01 / 1.121e+02	6.206e+01 / 1.064e+02	5.327e+01 / 7.369e+01
F20	7.808e+02 / 1.197e+03	5.517e+02 / 1.061e+03	3.701e+02 / 1.155e+03	7.795e+02 / 1.349e+03	6.302e+02 / 1.381e+03	5.216e+02 / 1.460e+03
F21	2.428e+02 / 2.578e+02	2.462e+02 / 2.573e+02	2.447e+02 / 2.539e+02	2.445e+02 / 2.581e+02	2.458e+02 / 2.636e+02	2.338e+02 / 2.417e+02
F22	9.177e+03 / 1.156e+04	9.977e+03 / 1.119e+04	8.561e+03 / 1.133e+04	1.000e+02 / 1.024e+04	1.000e+02 / 1.066e+04	8.159e+03 / 9.701e+03
F23	5.390e+02 / 5.629e+02	5.487e+02 / 5.645e+02	5.428e+02 / 5.643e+02	5.501e+02 / 5.761e+02	5.432e+02 / 5.712e+02	5.605e+02 / 5.819e+02
F24	8.520e+02 / 8.869e+02	8.913e+02 / 9.139e+02	8.917e+02 / 9.069e+02	8.987e+02 / 9.156e+02	8.826e+02 / 9.026e+02	8.891e+02 / 9.194e+02
F25	6.304e+02 / 7.102e+02	6.293e+02 / 7.441e+02	6.315e+02 / 7.428e+02	5.770e+02 / 7.049e+02	6.386e+02 / 7.616e+02	6.371e+02 / 7.044e+02
F26	2.946e+03 / 3.121e+03	3.134e+03 / 3.279e+03	3.038e+03 / 3.282e+03	3.000e+02 / 3.187e+03	3.119e+03 / 3.281e+03	2.978e+03 / 3.129e+03
F27	5.000e+02 / 5.000e+02	5.000e+02 / 5.000e+02	5.000e+02 / 5.000e+02	5.485e+02 / 5.862e+02	5.385e+02 / 5.865e+02	5.697e+02 / 5.897e+02
F28	4.833e+02 / 5.000e+02	4.761e+02 / 5.000e+02	4.764e+02 / 5.000e+02	3.000e+02 / 5.187e+02	4.782e+02 / 5.237e+02	4.782e+02 / 5.187e+02
F29	5.652e+02 / 8.121e+02	6.387e+02 / 9.277e+02	6.077e+02 / 8.703e+02	8.607e+02 / 1.276e+03	8.894e+02 / 1.238e+03	1.021e+03 / 1.528e+03
F30	7.304e+02 / 1.036e+03	7.440e+02 / 1.065e+03	6.888e+02 / 9.630e+02	2.180e+03 / 2.355e+03	2.115e+03 / 2.317e+03	2.102e+03 / 2.375e+03
N_{med}	2	2	2	2	2	7
N_{best}	2	2	2	6	3	7

Entries are best / median objective errors computed over 51 independent runs. Lower values indicate better performance. Best denotes the minimum error observed across the 51 runs. N_{med} and N_{best} denote the numbers of functions for which each algorithm attained the lowest median and lowest best value, respectively. Ties are counted for all tied algorithms.

S7 Table. Comparison of L-SRTDE-ALP, L-SRTDE-ADCP, and L-SRTDE-CP on the CEC 2017 test suite (30-dimensional). Results are based on Wilcoxon rank-sum tests at the significance level $\alpha = 0.05$.

	L-SRTDE-ALP MEAN (STD DEV)	L-SRTDE-ADCP MEAN (STD DEV)	L-SRTDE-CP MEAN (STD DEV)
F1	0.000e+00 (0.000e+00)	0.000e+00 (0.000e+00) ≈	0.000e+00 (0.000e+00) ≈
F3	0.000e+00 (0.000e+00)	0.000e+00 (0.000e+00) ≈	0.000e+00 (0.000e+00) ≈
F4	1.529e+01 (1.779e+00)	1.574e+01 (3.996e+00) +	1.666e+01 (2.677e+00) +
F5	3.056e+00 (1.835e+00)	2.653e+00 (1.330e+00) ≈	3.323e+00 (1.934e+00) ≈
F6	5.056e-07 (7.526e-07)	8.146e-07 (6.262e-07) +	3.630e-07 (6.403e-07) ≈
F7	3.556e+01 (1.646e+00)	3.628e+01 (2.629e+00) ≈	3.553e+01 (1.485e+00) ≈
F8	2.244e+00 (1.500e+00)	3.317e+00 (4.737e-01) +	2.068e+00 (1.391e+00) ≈
F9	0.000e+00 (0.000e+00)	0.000e+00 (0.000e+00) ≈	0.000e+00 (0.000e+00) ≈
F10	2.884e+02 (2.120e+02)	5.114e+02 (1.973e+02) +	2.776e+02 (2.000e+02) ≈
F11	1.602e+00 (2.024e+00)	8.979e-01 (1.021e+00) ≈	1.485e+00 (2.016e+00) ≈
F12	1.777e+00 (1.967e+00)	2.138e+00 (1.927e+00) -	1.420e+00 (1.839e+00) ≈
F13	9.594e+00 (6.749e+00)	8.836e+00 (6.937e+00) +	8.735e+00 (6.985e+00) ≈
F14	4.868e+00 (8.683e+00)	1.028e-02 (1.775e-02) -	5.678e+00 (9.068e+00) ≈
F15	4.708e-01 (2.905e-01)	3.883e-01 (3.100e-02) -	4.568e-01 (2.658e-01) ≈
F16	4.731e+00 (3.245e+00)	6.535e+00 (2.811e+00) ≈	5.065e+00 (3.222e+00) ≈
F17	2.166e+01 (4.611e+00)	2.267e+01 (8.677e-01) +	2.113e+01 (5.315e+00) ≈
F18	1.588e+01 (8.620e+00)	1.062e+01 (1.004e+01) -	1.703e+01 (7.729e+00) ≈
F19	2.098e+00 (5.749e-01)	1.656e+00 (1.359e-01) -	2.065e+00 (5.353e-01) ≈
F20	1.142e+01 (3.159e+01)	1.849e+00 (5.436e+00) ≈	6.009e+00 (9.075e+00) ≈
F21	2.023e+02 (1.911e+00)	2.023e+02 (4.911e-01) ≈	2.025e+02 (1.606e+00) ≈
F22	1.000e+02 (0.000e+00)	1.000e+02 (0.000e+00) ≈	1.000e+02 (0.000e+00) ≈
F23	3.402e+02 (3.718e+00)	3.368e+02 (5.302e+00) -	3.392e+02 (3.398e+00) ≈
F24	4.159e+02 (4.568e+00)	4.139e+02 (4.091e+00) -	4.145e+02 (4.312e+00) ≈
F25	3.792e+02 (9.469e-01)	3.789e+02 (9.271e-01) ≈	3.789e+02 (8.782e-01) ≈
F26	6.272e+02 (1.345e+02)	5.785e+02 (1.206e+02) ≈	6.180e+02 (1.360e+02) ≈
F27	4.584e+02 (4.843e+00)	4.614e+02 (4.889e+00) +	4.608e+02 (3.960e+00) +
F28	3.020e+02 (1.446e+01)	3.000e+02 (0.000e+00) ≈	3.043e+02 (2.129e+01) ≈
F29	3.707e+02 (9.628e+00)	3.954e+02 (9.076e+00) +	3.931e+02 (1.197e+01) +
F30	3.703e+02 (4.533e+01)	6.698e+02 (3.014e+02) +	8.606e+02 (3.804e+02) +
+/-/≈	-	9/7/13	4/0/25

Entries are mean (standard deviation) objective values computed over independent runs. Symbols denote the outcome of two-sided Wilcoxon rank-sum tests using L-SRTDE-ALP as the reference: "+" indicates that L-SRTDE-ALP performed significantly better than the comparator; "-" indicates that L-SRTDE-ALP performed significantly worse; and "≈" indicates no statistically significant difference. The final row reports per-configuration counts of wins/losses/ties versus the reference (format: wins/losses/ties).

S8 Table. Best and median objective errors obtained by L-SRTDE-ALP, L-SRTDE-ADCP, and L-SRTDE-CP on the CEC 2017 test suite (30-dimensional).

	L-SRTDE-ALP BEST / MEDIAN	L-SRTDE-ADCP BEST / MEDIAN	L-SRTDE-CP BEST / MEDIAN
F1	0.000e+00 / 0.000e+00	0.000e+00 / 0.000e+00	0.000e+00 / 0.000e+00
F3	0.000e+00 / 0.000e+00	0.000e+00 / 0.000e+00	0.000e+00 / 0.000e+00
F4	1.369e+01 / 1.466e+01	0.000e+00 / 1.485e+01	1.043e+01 / 1.493e+01
F5	0.000e+00 / 2.985e+00	9.950e-01 / 2.985e+00	0.000e+00 / 2.985e+00
F6	0.000e+00 / 1.369e-07	0.000e+00 / 3.080e-07	0.000e+00 / 1.369e-07
F7	3.323e+01 / 3.541e+01	3.247e+01 / 3.565e+01	3.290e+01 / 3.535e+01
F8	0.000e+00 / 1.990e+00	2.985e+00 / 2.985e+00	0.000e+00 / 1.990e+00
F9	0.000e+00 / 0.000e+00	0.000e+00 / 0.000e+00	0.000e+00 / 0.000e+00
F10	4.678e+00 / 2.467e+02	2.447e+02 / 4.756e+02	3.623e+00 / 2.437e+02
F11	0.000e+00 / 0.000e+00	0.000e+00 / 9.950e-01	0.000e+00 / 0.000e+00
F12	1.388e-01 / 4.164e-01	2.848e-01 / 3.544e-01	2.082e-01 / 4.164e-01
F13	0.000e+00 / 1.383e+01	4.350e-02 / 1.468e+01	0.000e+00 / 1.356e+01
F14	0.000e+00 / 1.152e-06	0.000e+00 / 0.000e+00	0.000e+00 / 0.000e+00
F15	5.467e-02 / 4.201e-01	3.498e-01 / 3.884e-01	7.281e-02 / 4.187e-01
F16	8.253e-01 / 2.684e+00	1.230e+00 / 7.924e+00	9.518e-01 / 2.927e+00
F17	2.136e+00 / 2.199e+01	2.102e+01 / 2.250e+01	6.256e-01 / 2.177e+01
F18	4.479e-01 / 2.053e+01	4.893e-01 / 2.013e+01	4.768e-01 / 2.053e+01
F19	1.043e+00 / 1.940e+00	1.500e+00 / 1.647e+00	1.437e+00 / 1.859e+00
F20	0.000e+00 / 3.123e-01	2.082e-01 / 3.123e-01	0.000e+00 / 4.163e-01
F21	2.000e+02 / 2.019e+02	2.020e+02 / 2.020e+02	2.000e+02 / 2.030e+02
F22	1.000e+02 / 1.000e+02	1.000e+02 / 1.000e+02	1.000e+02 / 1.000e+02
F23	3.331e+02 / 3.406e+02	3.298e+02 / 3.386e+02	3.309e+02 / 3.386e+02
F24	4.054e+02 / 4.165e+02	4.082e+02 / 4.152e+02	4.033e+02 / 4.145e+02
F25	3.783e+02 / 3.785e+02	3.784e+02 / 3.785e+02	3.783e+02 / 3.784e+02
F26	3.000e+02 / 6.522e+02	4.000e+02 / 5.685e+02	4.000e+02 / 6.522e+02
F27	4.477e+02 / 4.592e+02	4.509e+02 / 4.641e+02	4.522e+02 / 4.610e+02
F28	3.000e+02 / 3.000e+02	3.000e+02 / 3.000e+02	3.000e+02 / 3.000e+02
F29	3.520e+02 / 3.704e+02	3.789e+02 / 3.909e+02	3.661e+02 / 3.969e+02
F30	2.893e+02 / 3.649e+02	4.051e+02 / 5.286e+02	4.087e+02 / 7.598e+02
N_{med}	15	13	17
N_{best}	22	11	17

Entries are best / median objective errors computed over 51 independent runs. Lower values indicate better performance. Best denotes the minimum error observed across the 51 runs. N_{med} and N_{best} denote the numbers of functions for which each algorithm attained the lowest median and lowest best value, respectively. Ties are counted for all tied algorithms.

S9 Table. Comparison of L-SRTDE-ALP, L-SRTDE-ADCP, and L-SRTDE-CP on the CEC 2017 test suite (50-dimensional). Results are based on Wilcoxon rank-sum tests at the significance level $\alpha = 0.05$.

	L-SRTDE-ALP MEAN (STD DEV)	L-SRTDE-ADCP MEAN (STD DEV)	L-SRTDE-CP MEAN (STD DEV)
F1	3.767e-09 (1.095e-08)	7.783e-10 (5.558e-09) -	9.480e-10 (5.152e-09) ≈
F3	2.205e-10 (1.575e-09)	0.000e+00 (0.000e+00) ≈	0.000e+00 (0.000e+00) ≈
F4	2.373e+01 (1.467e+01)	2.086e+01 (5.675e-02) +	3.530e+01 (3.562e+01) +
F5	5.697e+00 (2.050e+00)	6.165e+00 (1.929e+00) +	5.892e+00 (1.698e+00) ≈
F6	1.773e-05 (8.122e-06)	2.228e-05 (1.307e-05) ≈	9.575e-06 (6.469e-06) -
F7	6.084e+01 (2.849e+00)	5.988e+01 (2.480e+00) ≈	6.070e+01 (2.787e+00) ≈
F8	5.658e+00 (2.082e+00)	6.204e+00 (1.204e+00) ≈	5.111e+00 (2.106e+00) ≈
F9	0.000e+00 (0.000e+00)	0.000e+00 (0.000e+00) ≈	0.000e+00 (0.000e+00) ≈
F10	4.994e+02 (2.561e+02)	6.643e+02 (2.563e+02) +	4.455e+02 (2.914e+02) ≈
F11	1.112e+01 (8.606e-01)	1.054e+01 (7.326e-01) -	1.126e+01 (1.071e+00) ≈
F12	1.072e+02 (8.108e+01)	9.592e+01 (5.649e+01) -	1.101e+02 (5.996e+01) ≈
F13	3.679e+00 (1.815e+00)	2.495e+00 (1.353e+00) -	3.995e+00 (2.392e+00) ≈
F14	1.969e+01 (5.521e+00)	2.101e+01 (9.424e-01) ≈	2.074e+01 (2.855e+00) ≈
F15	1.433e+01 (7.378e-01)	1.404e+01 (6.350e-01) ≈	1.451e+01 (8.727e-01) ≈
F16	1.272e+02 (1.654e+01)	1.253e+02 (8.892e-01) +	1.251e+02 (1.648e+00) ≈
F17	1.025e+02 (6.695e+01)	1.321e+02 (6.328e+01) +	1.016e+02 (6.885e+01) ≈
F18	2.056e+01 (6.438e-02)	2.087e+01 (4.728e-01) ≈	2.064e+01 (2.120e-01) +
F19	3.962e+00 (6.303e-01)	3.702e+00 (5.241e-01) -	3.967e+00 (5.735e-01) ≈
F20	2.615e+01 (6.254e+00)	2.522e+01 (6.593e+00) ≈	2.594e+01 (3.699e+00) ≈
F21	2.068e+02 (2.172e+00)	2.073e+02 (2.740e+00) ≈	2.064e+02 (2.281e+00) ≈
F22	1.000e+02 (0.000e+00)	1.000e+02 (0.000e+00) ≈	1.000e+02 (0.000e+00) ≈
F23	4.025e+02 (6.526e+00)	4.029e+02 (6.515e+00) ≈	4.039e+02 (6.202e+00) ≈
F24	4.847e+02 (4.080e+00)	4.870e+02 (2.998e+00) +	4.854e+02 (4.114e+00) ≈
F25	4.779e+02 (1.980e-01)	4.782e+02 (1.792e-01) +	4.792e+02 (2.173e-01) +
F26	5.193e+02 (1.404e+02)	4.865e+02 (1.230e+02) ≈	5.228e+02 (1.384e+02) ≈
F27	4.482e+02 (1.023e+01)	4.550e+02 (9.986e+00) +	4.536e+02 (9.339e+00) +
F28	4.502e+02 (7.086e+00)	4.502e+02 (3.355e-01) +	4.513e+02 (2.177e-01) +
F29	2.464e+02 (5.871e+00)	2.578e+02 (1.878e+01) +	2.574e+02 (1.453e+01) +
F30	8.452e+02 (1.406e+02)	2.238e+03 (2.629e+03) +	1.338e+04 (1.625e+04) +
+/-/≈	-	11/5/13	7/1/21

Entries are mean (standard deviation) objective values computed over independent runs. Symbols denote the outcome of two-sided Wilcoxon rank-sum tests using L-SRTDE-ALP as the reference: "+" indicates that L-SRTDE-ALP performed significantly better than the comparator; "-" indicates that L-SRTDE-ALP performed significantly worse; and "≈" indicates no statistically significant difference. The final row reports per-configuration counts of wins/losses/ties versus the reference (format: wins/losses/ties).

S10 Table. Best and median objective errors obtained by L-SRTDE-ALP, L-SRTDE-ADCP, and L-SRTDE-CP on the CEC 2017 test suite (50-dimensional).

	L-SRTDE-ALP BEST / MEDIAN	L-SRTDE-ADCP BEST / MEDIAN	L-SRTDE-CP BEST / MEDIAN
F1	0.000e+00 / 0.000e+00	0.000e+00 / 0.000e+00	0.000e+00 / 0.000e+00
F3	0.000e+00 / 0.000e+00	0.000e+00 / 0.000e+00	0.000e+00 / 0.000e+00
F4	2.073e+01 / 2.080e+01	2.078e+01 / 2.084e+01	2.105e+01 / 2.137e+01
F5	1.990e+00 / 4.975e+00	0.000e+00 / 6.965e+00	2.985e+00 / 5.970e+00
F6	5.133e-06 / 1.531e-05	1.246e-05 / 1.561e-05	9.402e-07 / 7.696e-06
F7	5.553e+01 / 6.044e+01	5.681e+01 / 5.982e+01	5.609e+01 / 6.052e+01
F8	9.950e-01 / 4.975e+00	3.980e+00 / 5.970e+00	9.950e-01 / 4.975e+00
F9	0.000e+00 / 0.000e+00	0.000e+00 / 0.000e+00	0.000e+00 / 0.000e+00
F10	1.260e+02 / 5.240e+02	1.284e+02 / 7.601e+02	1.228e+02 / 3.709e+02
F11	1.007e+01 / 1.116e+01	1.013e+01 / 1.024e+01	1.018e+01 / 1.122e+01
F12	1.370e+01 / 1.343e+02	1.495e+01 / 1.326e+02	7.027e+00 / 1.391e+02
F13	4.007e-01 / 3.796e+00	3.360e-01 / 2.912e+00	1.306e-01 / 4.038e+00
F14	1.567e-02 / 2.103e+01	2.003e+01 / 2.104e+01	2.023e+00 / 2.104e+01
F15	1.332e+01 / 1.436e+01	1.305e+01 / 1.391e+01	1.322e+01 / 1.421e+01
F16	1.217e+02 / 1.248e+02	1.237e+02 / 1.256e+02	1.211e+02 / 1.252e+02
F17	2.183e+01 / 1.486e+02	2.419e+01 / 1.625e+02	2.519e+01 / 1.470e+02
F18	2.052e+01 / 2.055e+01	2.052e+01 / 2.055e+01	2.052e+01 / 2.058e+01
F19	2.622e+00 / 4.014e+00	2.518e+00 / 3.774e+00	2.548e+00 / 3.897e+00
F20	2.031e+01 / 2.541e+01	2.138e+01 / 2.333e+01	2.136e+01 / 2.488e+01
F21	2.030e+02 / 2.072e+02	2.030e+02 / 2.079e+02	2.016e+02 / 2.064e+02
F22	1.000e+02 / 1.000e+02	1.000e+02 / 1.000e+02	1.000e+02 / 1.000e+02
F23	3.908e+02 / 4.027e+02	3.890e+02 / 4.038e+02	3.885e+02 / 4.039e+02
F24	4.766e+02 / 4.849e+02	4.757e+02 / 4.878e+02	4.716e+02 / 4.862e+02
F25	4.774e+02 / 4.779e+02	4.778e+02 / 4.782e+02	4.786e+02 / 4.792e+02
F26	4.000e+02 / 4.000e+02	4.000e+02 / 4.000e+02	4.000e+02 / 4.000e+02
F27	4.396e+02 / 4.459e+02	4.430e+02 / 4.553e+02	4.429e+02 / 4.517e+02
F28	4.485e+02 / 4.492e+02	4.497e+02 / 4.502e+02	4.508e+02 / 4.513e+02
F29	2.353e+02 / 2.457e+02	2.361e+02 / 2.491e+02	2.366e+02 / 2.545e+02
F30	5.352e+02 / 8.576e+02	7.454e+02 / 1.233e+03	1.238e+03 / 6.181e+03
N_{med}	18	12	10
N_{best}	17	9	14

Entries are best / median objective errors computed over 51 independent runs. Lower values indicate better performance. Best denotes the minimum error observed across the 51 runs. N_{med} and N_{best} denote the numbers of functions for which each algorithm attained the lowest median and lowest best value, respectively. Ties are counted for all tied algorithms.

S11 Table. Comparison of L-SRTDE-ALP, L-SRTDE-ADCP, and L-SRTDE-CP on the CEC 2017 test suite (100-dimensional). Results are based on Wilcoxon rank-sum tests at the significance level $\alpha = 0.05$.

	L-SRTDE-ALP MEAN (STD DEV)	L-SRTDE-ADCP MEAN (STD DEV)	L-SRTDE-CP MEAN (STD DEV)
F1	2.486e+03 (2.739e+03)	3.548e+03 (2.960e+03) +	7.185e+02 (1.361e+03) -
F3	5.690e-02 (5.352e-02)	5.073e-02 (2.044e-02) ≈	1.426e-02 (9.972e-03) -
F4	1.806e+02 (2.799e+01)	1.788e+02 (2.476e+01) ≈	1.901e+02 (2.317e+01) +
F5	1.727e+01 (3.641e+00)	1.732e+01 (3.430e+00) ≈	1.748e+01 (3.413e+00) ≈
F6	3.137e-04 (7.256e-05)	4.238e-04 (1.009e-04) +	2.411e-04 (5.864e-05) -
F7	1.165e+02 (5.480e+00)	1.155e+02 (3.748e+00) ≈	1.164e+02 (5.517e+00) ≈
F8	1.717e+01 (3.647e+00)	1.530e+01 (2.453e+00) -	1.621e+01 (3.431e+00) ≈
F9	0.000e+00 (0.000e+00)	0.000e+00 (0.000e+00) ≈	0.000e+00 (0.000e+00) ≈
F10	1.782e+03 (6.178e+02)	1.644e+03 (4.422e+02) ≈	1.565e+03 (6.123e+02) ≈
F11	2.820e+01 (2.226e+01)	2.739e+01 (2.347e+01) ≈	2.818e+01 (2.256e+01) ≈
F12	2.744e+03 (1.923e+03)	2.768e+03 (1.581e+03) ≈	2.098e+03 (9.542e+02) ≈
F13	4.321e+01 (2.003e+01)	4.599e+01 (1.703e+01) ≈	4.990e+01 (2.103e+01) +
F14	2.571e+01 (1.844e+00)	2.537e+01 (1.604e+00) ≈	2.554e+01 (1.586e+00) ≈
F15	5.769e+01 (3.159e+01)	5.349e+01 (3.108e+01) ≈	6.048e+01 (3.257e+01) ≈
F16	1.120e+02 (6.082e+01)	8.929e+01 (5.924e+01) ≈	9.668e+01 (8.472e+01) ≈
F17	7.575e+01 (5.010e+01)	7.407e+01 (5.250e+01) ≈	7.226e+01 (4.892e+01) ≈
F18	2.510e+01 (1.805e+00)	2.582e+01 (1.332e+00) +	2.550e+01 (1.706e+00) ≈
F19	1.556e+01 (2.183e+00)	1.448e+01 (2.165e+00) -	1.545e+01 (2.319e+00) ≈
F20	1.700e+02 (1.813e+01)	1.698e+02 (1.687e+01) ≈	1.761e+02 (3.099e+01) ≈
F21	2.385e+02 (3.568e+00)	2.383e+02 (4.432e+00) ≈	2.367e+02 (3.725e+00) -
F22	2.025e+03 (9.544e+02)	2.241e+03 (9.414e+02) ≈	2.297e+03 (9.047e+02) ≈
F23	5.370e+02 (8.015e+00)	5.359e+02 (9.468e+00) ≈	5.374e+02 (8.747e+00) ≈
F24	8.126e+02 (1.342e+01)	8.157e+02 (1.589e+01) ≈	8.137e+02 (1.354e+01) ≈
F25	6.245e+02 (3.572e+01)	6.240e+02 (3.079e+01) +	6.239e+02 (2.901e+01) +
F26	2.179e+03 (2.335e+02)	2.168e+03 (2.228e+02) ≈	2.203e+03 (2.111e+02) ≈
F27	5.000e+02 (4.675e-04)	5.000e+02 (2.456e-01) +	5.000e+02 (9.569e-04) +
F28	5.000e+02 (6.190e-04)	4.957e+02 (7.496e+00) +	4.973e+02 (7.624e+00) +
F29	5.440e+02 (4.389e+01)	5.626e+02 (4.202e+01) +	5.607e+02 (1.879e+01) +
F30	5.858e+02 (6.495e+01)	1.126e+03 (2.072e+02) +	1.210e+03 (1.473e+02) +
+/-/≈	-	8/2/19	7/4/18

Entries are mean (standard deviation) objective values computed over independent runs. Symbols denote the outcome of two-sided Wilcoxon rank-sum tests using L-SRTDE-ALP as the reference: "+" indicates that L-SRTDE-ALP performed significantly better than the comparator; "-" indicates that L-SRTDE-ALP performed significantly worse; and "≈" indicates no statistically significant difference. The final row reports per-configuration counts of wins/losses/ties versus the reference (format: wins/losses/ties).

S12 Table. Best and median objective errors obtained by L-SRTDE-ALP, L-SRTDE-ADCP, and L-SRTDE-CP on the CEC 2017 test suite (100-dimensional).

	L-SRTDE-ALP BEST / MEDIAN	L-SRTDE-ADCP BEST / MEDIAN	L-SRTDE-CP BEST / MEDIAN
F1	5.539e+01 / 1.708e+03	2.746e+02 / 2.504e+03	2.615e-01 / 3.106e+02
F3	1.191e-02 / 4.523e-02	1.683e-02 / 4.419e-02	2.970e-03 / 1.102e-02
F4	8.380e+01 / 1.970e+02	1.422e+02 / 1.879e+02	1.454e+02 / 2.027e+02
F5	1.094e+01 / 1.691e+01	1.194e+01 / 1.592e+01	1.293e+01 / 1.691e+01
F6	1.848e-04 / 3.000e-04	2.551e-04 / 3.953e-04	1.026e-04 / 2.404e-04
F7	1.090e+02 / 1.157e+02	1.099e+02 / 1.154e+02	1.090e+02 / 1.146e+02
F8	9.950e+00 / 1.791e+01	8.955e+00 / 1.492e+01	9.950e+00 / 1.592e+01
F9	0.000e+00 / 0.000e+00	0.000e+00 / 0.000e+00	0.000e+00 / 0.000e+00
F10	4.725e+02 / 1.829e+03	6.488e+02 / 1.657e+03	5.509e+02 / 1.640e+03
F11	1.046e+01 / 1.973e+01	1.240e+01 / 1.879e+01	1.404e+01 / 1.969e+01
F12	7.018e+02 / 2.187e+03	1.061e+03 / 2.369e+03	5.355e+02 / 1.948e+03
F13	2.024e+01 / 3.534e+01	2.208e+01 / 4.314e+01	1.406e+01 / 4.738e+01
F14	2.254e+01 / 2.537e+01	2.133e+01 / 2.532e+01	2.164e+01 / 2.538e+01
F15	1.359e+01 / 7.843e+01	1.601e+01 / 7.496e+01	1.586e+01 / 8.022e+01
F16	1.109e+01 / 1.335e+02	1.304e+01 / 1.318e+02	1.144e+01 / 1.278e+02
F17	3.727e+01 / 5.104e+01	3.113e+01 / 4.566e+01	3.309e+01 / 4.769e+01
F18	2.174e+01 / 2.499e+01	2.404e+01 / 2.544e+01	2.190e+01 / 2.539e+01
F19	1.056e+01 / 1.558e+01	1.031e+01 / 1.368e+01	1.147e+01 / 1.488e+01
F20	1.542e+02 / 1.678e+02	1.557e+02 / 1.674e+02	1.571e+02 / 1.661e+02
F21	2.305e+02 / 2.390e+02	2.303e+02 / 2.387e+02	2.285e+02 / 2.365e+02
F22	1.000e+02 / 2.154e+03	1.000e+02 / 2.227e+03	1.000e+02 / 2.317e+03
F23	5.168e+02 / 5.373e+02	5.199e+02 / 5.348e+02	5.221e+02 / 5.373e+02
F24	7.842e+02 / 8.117e+02	7.905e+02 / 8.131e+02	7.876e+02 / 8.151e+02
F25	5.666e+02 / 6.215e+02	5.668e+02 / 6.246e+02	5.688e+02 / 6.291e+02
F26	1.588e+03 / 2.227e+03	1.484e+03 / 2.193e+03	1.544e+03 / 2.240e+03
F27	5.000e+02 / 5.000e+02	4.983e+02 / 5.000e+02	5.000e+02 / 5.000e+02
F28	5.000e+02 / 5.000e+02	4.753e+02 / 5.000e+02	4.761e+02 / 5.000e+02
F29	4.079e+02 / 5.497e+02	3.987e+02 / 5.615e+02	5.269e+02 / 5.540e+02
F30	5.070e+02 / 5.688e+02	5.632e+02 / 1.157e+03	1.093e+03 / 1.170e+03
N_{med}	10	11	10
N_{best}	14	9	8

Entries are best / median objective errors computed over 51 independent runs. Lower values indicate better performance. Best denotes the minimum error observed across the 51 runs. N_{med} and N_{best} denote the numbers of functions for which each algorithm attained the lowest median and lowest best value, respectively. Ties are counted for all tied algorithms.

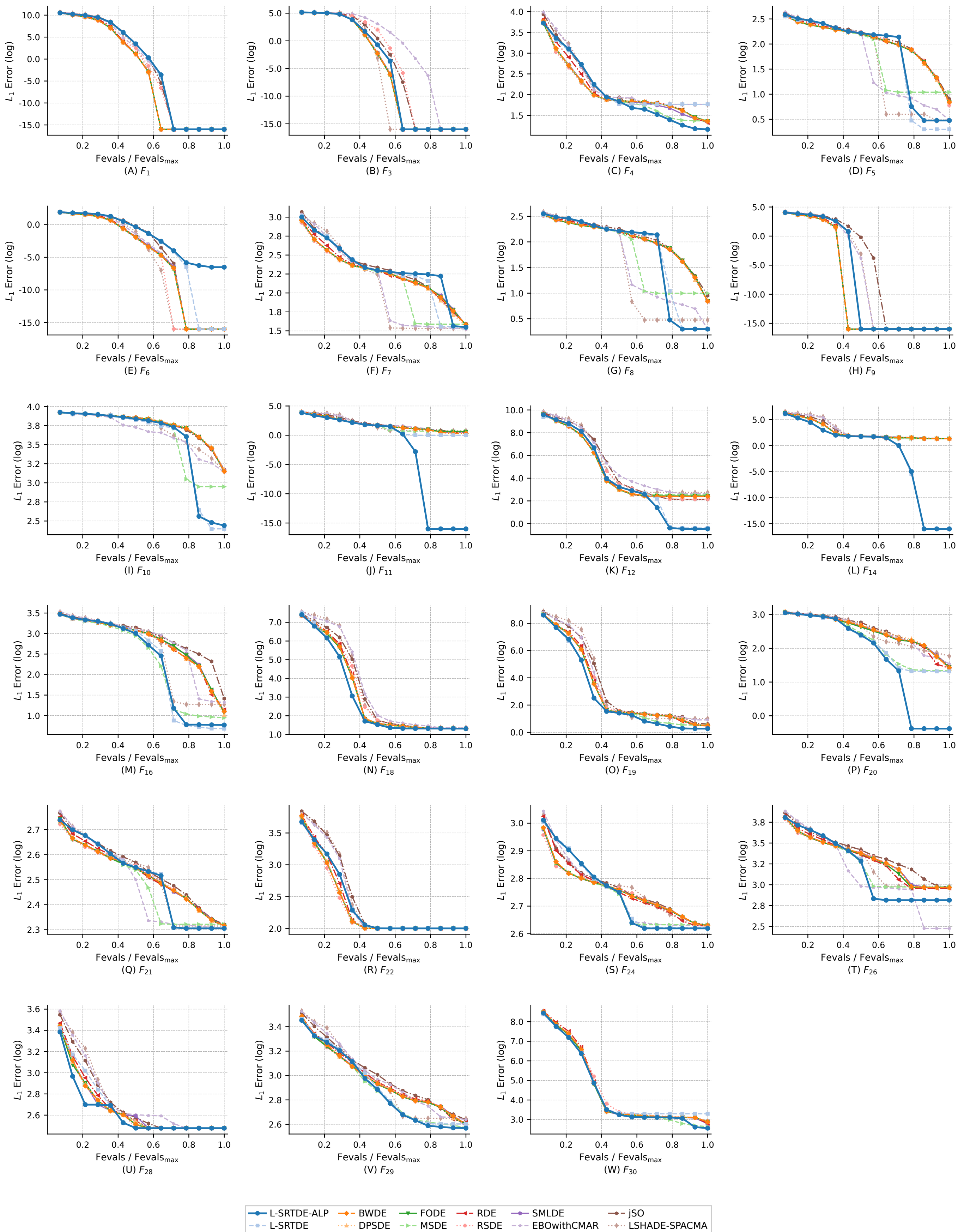

Fig A. Convergence curves of L-SRTDE-ALP and eleven competing algorithms on the 30-dimensional CEC 2017 test suite.

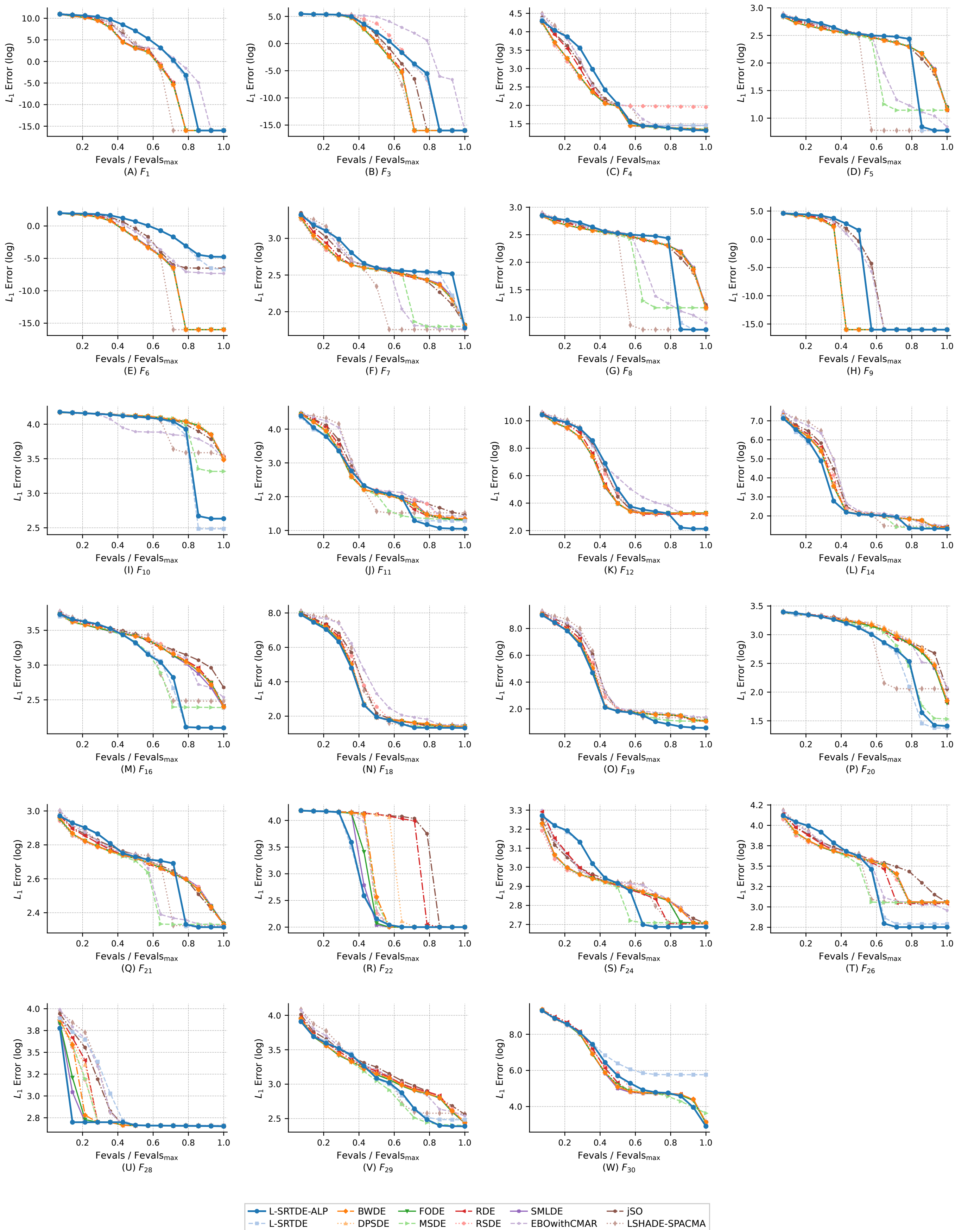

Fig B. Convergence curves of L-SRTDE-ALP and eleven competing algorithms on the 50-dimensional CEC 2017 test suite.

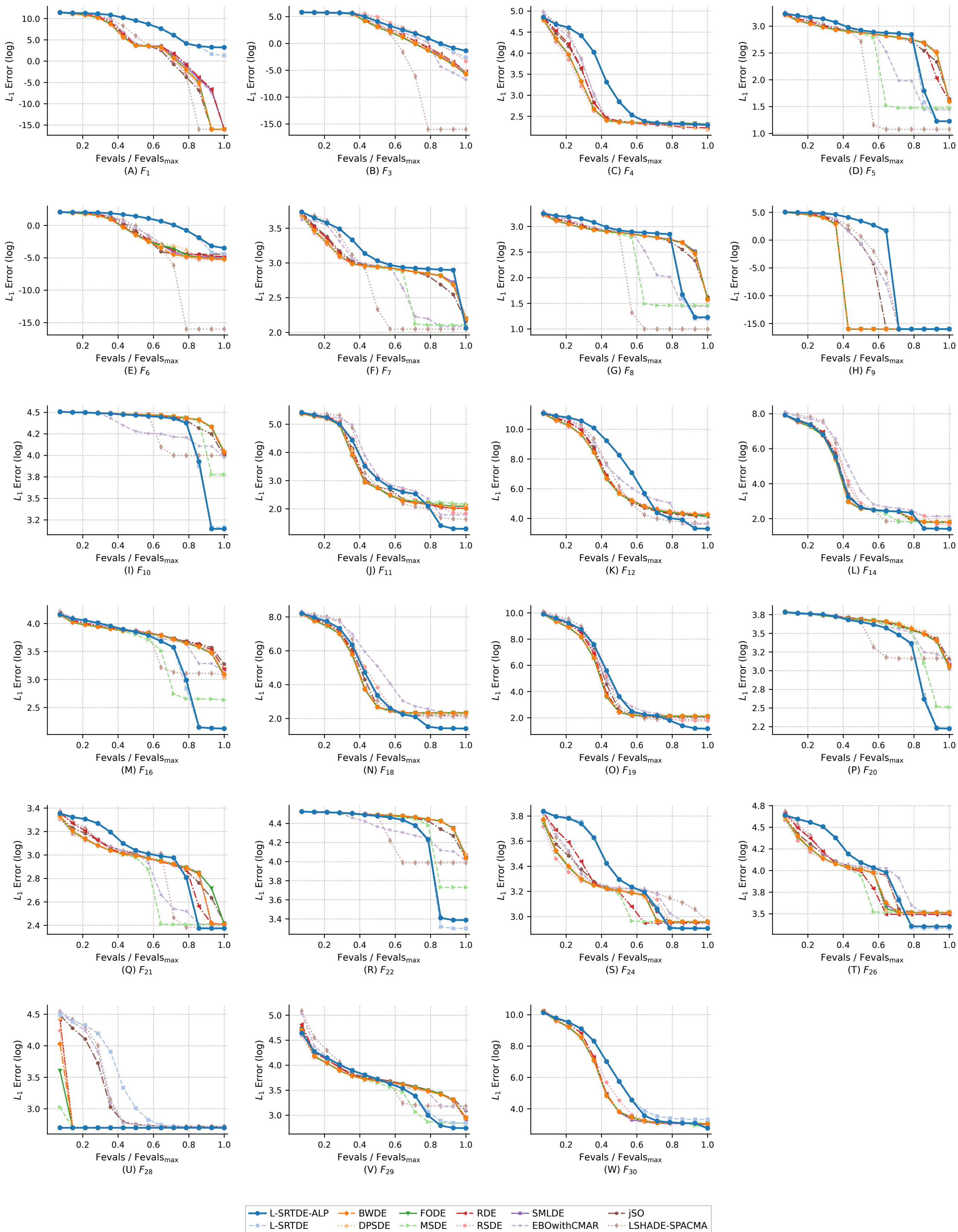

Fig C. Convergence curves of L-SRTDE-ALP and eleven competing algorithms on the 100-dimensional CEC 2017 test suite.

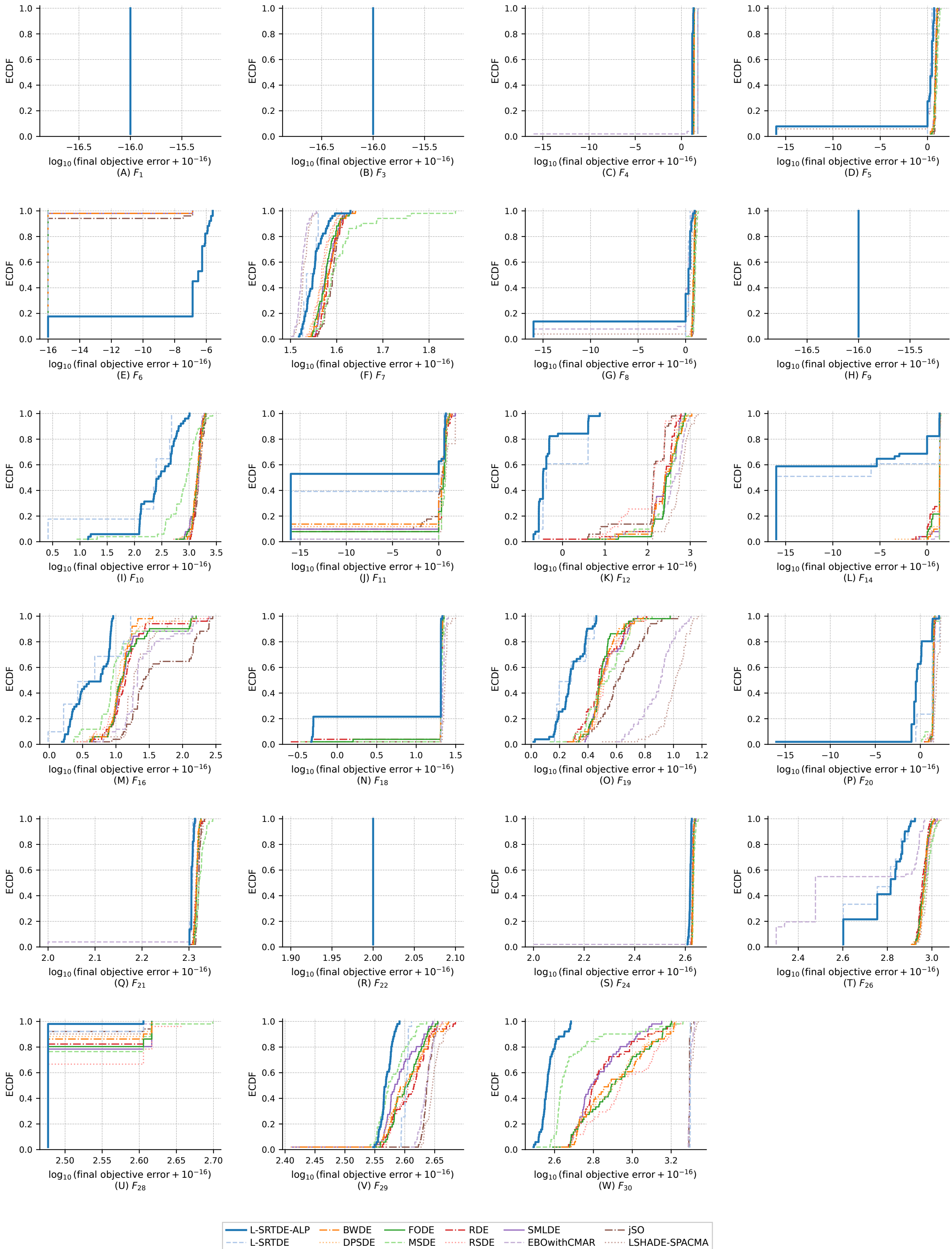

Fig D. Empirical cumulative distribution functions (ECDFs) of the final objective errors obtained by L-SRTDE-ALP and eleven competing algorithms on the 30-dimensional CEC 2017 test suite over 51 independent runs.

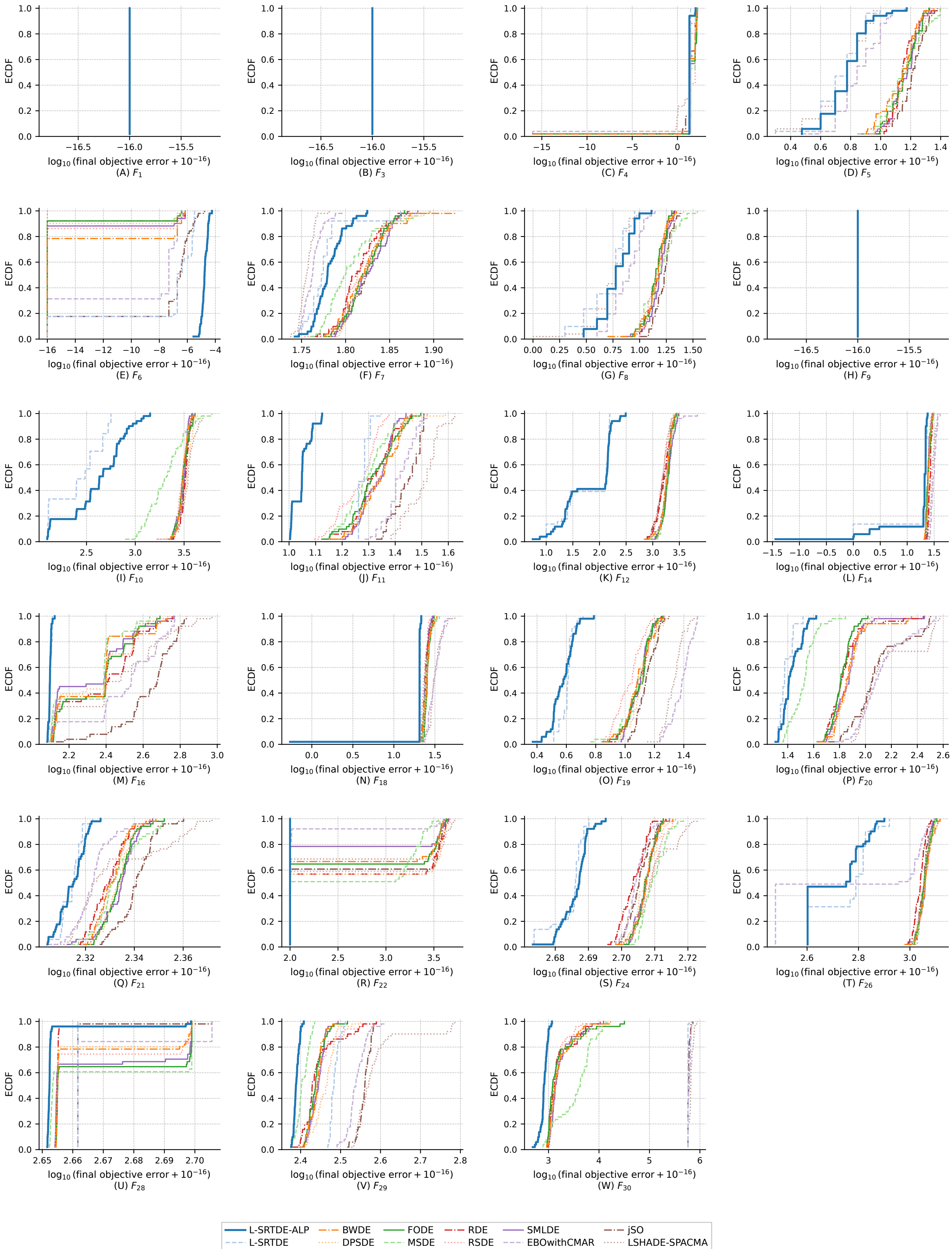

Fig E. Empirical cumulative distribution functions (ECDFs) of the final objective errors obtained by L-SRTDE-ALP and eleven competing algorithms on the 50-dimensional CEC 2017 test suite over 51 independent runs.

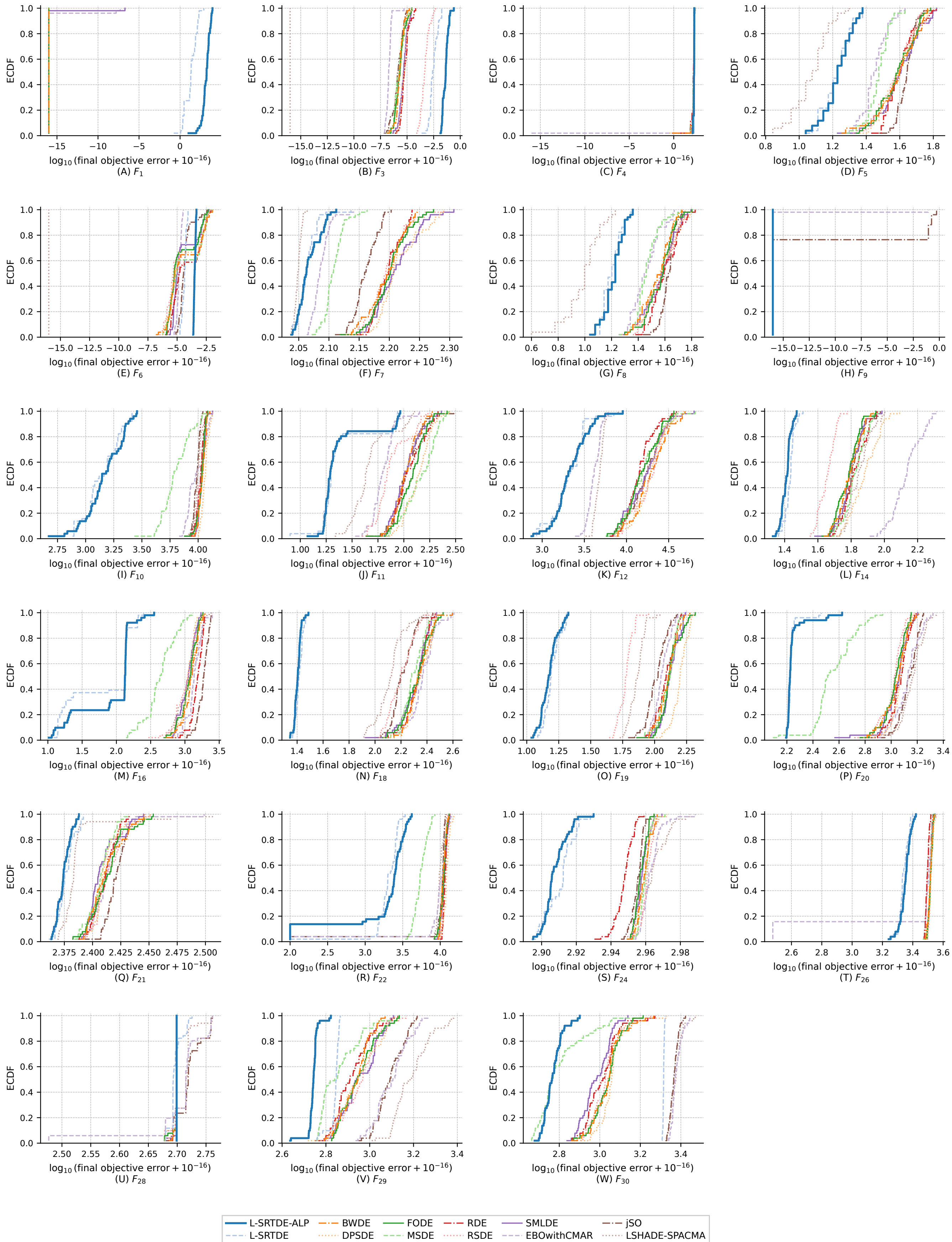

Fig F. Empirical cumulative distribution functions (ECDFs) of the final objective errors obtained by L-SRTDE-ALP and eleven competing algorithms on the 100-dimensional CEC 2017 test suite over 51 independent runs.
