# Supplementary material for: A rank-conditioned α-stable perturbation for differential evolution: Separating perturbation frequency and tail heaviness
Source: PLoS One. 2026 Sep 9;21(9):e0357907. doi: 10.1371/journal.pone.0357907 (PMC13557417; doi:10.1371/journal.pone.0357907)
Supplement: Supplementary file 2 [file pone.0357907.s003.pdf]

# Reviewers' Comments & Authors' Responses

| | |
|---|---|
| **Manuscript No.:** | #PONE-D-26-37177 |
| **Submitted to:** | *PLOS One* |
| **Title:** | "A rank-conditioned $\alpha$-stable perturbation for differential evolution: Separating perturbation frequency and tail heaviness" |
| **Authors:** | Tae Jong Choi and Yeji An |

Republic of Korea, August 14, 2026

Dear Prof. **Mahamed G.H. Omran**, *Academic Editor of PLOS One*,

We would like to sincerely thank you for handling the review process of our manuscript. We are also grateful to the anonymous Reviewer for the careful and constructive comments, which helped us substantially improve the technical soundness, clarity, reproducibility, and completeness of the revised manuscript.

In response to the comments received, we have carefully revised both the manuscript and the Supporting Information and conducted additional experiments and analyses to strengthen the empirical support for the proposed method. We have also moderated the interpretation of the results so that the conclusions accurately reflect the statistical evidence.

For convenience, the major revisions are summarized as follows:

- We substantially expanded the literature review on adaptive differential evolution, evolution strategies, and Lévy-based or heavy-tailed variation mechanisms. We now explicitly acknowledge that the general use of Lévy distributions in evolutionary optimization is not new and clarify that the specific contribution of ALP lies in directly adapting the stability index of the perturbation distribution according to individual fitness rank, independently of dimension-wise perturbation activation;
- We added comprehensive comparisons with the three leading CEC 2017 methods specifically identified by the Reviewer: EBOwithCMAR, jSO, and LSHADE-SPACMA. For these three methods, we used the publicly released run-level results from the official CEC 2017 benchmark repository. These results follow the same CEC 2017 evaluation budgets and 51-run protocol used in our experiments at $D \in \{30, 50, 100\}$;
- We substantially expanded the numerical and distributional reporting. The revised Supporting Information now presents the best, median, mean, and standard deviation for each algorithm, function, and dimensionality. We also added empirical cumulative distribution functions, complete convergence results, Wilcoxon rank-sum summaries, Friedman rankings, and multiplicity-adjusted post-hoc comparisons;
- We clarified the provenance and role of all algorithmic parameters. The parameters and fixed numerical constants inherited from L-SRTDE and ADCP are now explicitly distinguished from the two parameters introduced by ALP, $\alpha_{min}$ and $\alpha_{max}$.
- We added detailed reproducibility and computational information, including the pseudo-random number generator, the seed-generation procedure, the implementation environment, empirical runtime measurements, and asymptotic time and space complexity analyses;
- We regenerated and reorganized the convergence and ECDF figures to improve readability and publication quality. Selected results are presented in the main manuscript, while complete function-level graphical results are provided in the Supporting Information. We also revised the discussion and conclusion to characterize ALP as a competitive, low-overhead refinement of L-SRTDE rather than as a uniformly superior redesign.
- To strengthen transparency and reproducibility, we publicly released the author-generated source code and the run-level experimental data underlying the reported results at https://github.com/gry-kiu/L-SRTDE-ALP. The repository also documents the original sources of all third-party algorithm implementations and the publicly available CEC 2017 benchmark data used for EBOwithCMAR, jSO, and LSHADE-SPACMA;

Below, we first address each of the additional requirements raised by the Academic Editor and the journal, followed by a detailed point-by-point response to all comments raised by the Reviewer. The Reviewer's comments are shown in *blue italic text*, while our responses and the corresponding authors' actions are presented in normal font under separate headings.

We hope that these extensive revisions adequately address the concerns raised by the Editor and the Reviewer and that the revised manuscript now satisfies the technical and reporting standards of *PLOS ONE*. We respectfully submit the revised manuscript for your further consideration.

Sincerely,

**Yeji An**,
on behalf of all authors

# 1 Response to the Academic Editor and Journal Requirements

***Requirement 1:*** *Please ensure that your manuscript meets PLOS ONE's style requirements, including those for file naming.*

**Authors' response:** We have reviewed the PLOS ONE formatting and file-naming requirements and prepared the revised submission accordingly. The revised submission includes an unmarked manuscript, a marked-up version showing the revisions, the Supporting Information file, and the required figure files using the appropriate submission categories and file names.

**Authors' actions on the revised manuscript:** The revised manuscript and associated submission files have been checked and organized according to the PLOS ONE submission and file-naming requirements.

***Requirement 2:*** *Please note that PLOS ONE has specific guidelines on code sharing for submissions in which author-generated code underpins the findings in the manuscript.*

**Authors' response:** We agree that the author-generated code underlying the findings should be publicly accessible. We have therefore made the source code for L-SRTDE-ALP publicly available at:

`https://github.com/gry-kiu/L-SRTDE-ALP`

The repository also provides the experimental data generated in this study and documents the original sources of the publicly available third-party algorithm implementations used in the comparisons.

For EBOwithCMAR, jSO, and LSHADE-SPACMA, we did not reimplement the algorithms. Instead, we used the publicly available CEC 2017 benchmark results released at `https://github.com/P-N-Suganthan/CEC2017-BoundContrained`. The provenance of these third-party benchmark data is explicitly documented in our repository.

**Authors' actions on the revised manuscript:** The author-generated source code and experimental data have been publicly released, and the corresponding repository information has been added to the Data Availability information associated with the revised submission.

***Requirement 3:*** *The grant information provided in the Funding Information and Financial Disclosure sections does not match.*

**Authors' response:** Thank you for pointing out this inconsistency. We have carefully checked the funding information and corrected the corresponding entries so that the grant numbers reported in the Funding Information and Financial Disclosure sections are identical.

The correct grant numbers are: **[This work was supported by the National Research Foundation of Korea (NRF) grant funded by the Korea government (MSIT) (No. RS-2023-00214326 and No. RS-2026-25490120). This work was supported by the Ministry of Education of the Republic of Korea and the National Research Foundation of Korea (NRF-2023S1A5A8079831).]**.

**Authors' actions on the revised manuscript:** The funding information in the online submission system has been corrected accordingly.

***Requirement 4:*** *Please state what role the funders took in the study.*

**Authors' response:** We confirm that the funders had no role in the conduct or reporting of this study. As requested, the following statement has been included in the revised cover letter:

> "The funders had no role in study design, data collection and analysis, decision to publish, or preparation of the manuscript."

**Authors' actions on the revised manuscript:** The requested Role of Funder statement has been included in the revised cover letter.

> ***Requirement 5:*** *Please confirm that the declared relationship between the authors does not alter adherence to PLOS ONE policies on sharing data and materials.*

**Authors' response:** We confirm that the declared relationship does not affect our adherence to PLOS ONE policies. The updated Competing Interests statement is:

> "Authors Tae Jong Choi and Yeji An are spouses. The authors declare that this relationship did not influence the conduct or reporting of this study and that they have no competing financial interests. This does not alter our adherence to PLOS ONE policies on sharing data and materials."

**Authors' actions on the revised manuscript:** The updated Competing Interests statement has been included in the revised cover letter as requested.

> ***Requirement 6:*** *Please provide a data-sharing plan and ensure that the data underlying the findings are made freely accessible if the manuscript is accepted.*

**Authors' response:** We have now made the author-generated data underlying the findings of this study publicly available rather than waiting until acceptance. The data are available at:

`https://github.com/gry-kiu/L-SRTDE-ALP`

The repository includes run-level experimental data for L-SRTDE-ALP and the comparison algorithms evaluated by the authors, the controlled L-SRTDE-ALP/L-SRTDE-ADCP/L-SRTDE-CP experiments, the parameter-sensitivity experiments, and derived summary data used in the manuscript and Supporting Information.

The results for EBOwithCMAR, jSO, and LSHADE-SPACMA were obtained from the publicly available run-level CEC 2017 benchmark results at

`https://github.com/P-N-Suganthan/CEC2017-BoundContrained`.

These third-party data are not duplicated in our repository; instead, their exact source and role in the analysis are documented in the repository README.

**Authors' actions on the revised manuscript:** The Data Availability Statement in the submission system has been updated to provide the public repository and to distinguish author-generated data from the publicly available third-party CEC 2017 benchmark data.

> ***Requirement 7:*** *Please ensure that the title in the online submission form and the manuscript are identical.*

**Authors' response:** We have verified that the title entered in the online submission system is identical to the title of the revised manuscript.

**Authors' actions on the revised manuscript:** The title information has been checked and corrected where necessary.

***Requirement 8:*** *Please ensure that the abstract in the online submission form and the manuscript are identical.*

**Authors' response:** We have verified that the abstract entered in the online submission system is identical to the abstract of the revised manuscript.

**Authors' actions on the revised manuscript:** The abstract information has been checked and corrected where necessary.

***Requirement 9:*** *If the Reviewer recommends specific previously published works, please review and evaluate whether they are relevant and should be cited.*

**Authors' response:** We carefully reviewed the works suggested by the Reviewer and evaluated their relevance to the revised manuscript. The works by Das and Suganthan and by Beyer and Schwefel were directly relevant to the discussion of adaptive DE and evolution strategies and have been incorporated into the revised Literature Review. We also reviewed the additional literature suggested in connection with random-number generation and revised the description of the PRNG and seeding procedure accordingly.

**Authors' actions on the revised manuscript:** The relevant recommended literature has been incorporated into the revised manuscript, and the corresponding discussion has been expanded as described in our response to Reviewer Comments 2 and 5.

## 2 Response to the Comments of Reviewer 1

***Comment 1:*** *The underlying principle may be of interest, although it is not entirely novel. The algorithm introduces too many parameters without providing any justification for their choice. The experimental results are not sufficiently comprehensive to support the claimed comparisons. There are also no comparisons with the winners of the CEC 2017 competition. Overall, the proposed algorithm, in its present form, is not convincing.*

**Authors' response:** We sincerely thank the Reviewer for the careful and critical assessment of our manuscript. We agree that the original submission did not sufficiently distinguish the specific contribution of the proposed method from earlier Lévy-based DE approaches, did not clearly separate inherited parameters from parameters newly introduced by ALP, and did not provide sufficiently comprehensive experimental evidence.

We have therefore substantially revised both the manuscript and the Supporting Information. The principal revisions are summarized as follows:

- We expanded the literature review on adaptive DE, evolution strategies, and Lévy-based variation mechanisms and clarified that the use of a Lévy or heavy-tailed distribution in DE is not, by itself, the contribution of this work. The specific contribution of ALP is now positioned as **rank-conditioned, individual-level adaptation of the stability index of the perturbation distribution**, combined with an independent dimension-wise perturbation-activation mechanism.
- We added the three leading CEC 2017 methods identified by the Reviewer: EBOwithCMAR, jSO, and LSHADE-SPACMA. These methods are evaluated under the same dimensions, function-evaluation budgets, and numbers of independent runs as L-SRTDE-ALP.
- We substantially expanded the reported results. The Supporting Information now reports the best, median, mean, and standard deviation for each function, algorithm, and dimensionality. We also added empirical cumulative distribution functions (ECDFs) based on all independent runs, complete convergence results, Friedman rankings, and multiplicity-adjusted post-hoc comparisons.
- We clarified that nearly all parameters and numerical constants are inherited unchanged from L-SRTDE or ADCP and are fixed a priori. The only parameters introduced by ALP are $\alpha_{\min}$ and $\alpha_{\max}$. The revised manuscript now more clearly relates the justification of these two parameters to the sensitivity analysis over 20 parameter combinations that was already provided in the original submission.
- We explicitly documented the pseudo-random number generator and the seed-generation procedure applied consistently to all compared algorithms.
- We regenerated and reorganized the convergence and distributional figures to improve their readability and publication quality.
- We moderated the interpretation of the results. In particular, because the post-hoc analyses do not identify a statistically significant difference between L-SRTDE-ALP and its direct baseline L-SRTDE, the revised manuscript now describes ALP as a **conservative, low-overhead refinement** rather than a uniformly superior redesign.

We believe that these revisions substantially strengthen the technical justification, experimental support, and positioning of the proposed method. Detailed responses to each specific concern are provided below.

**Authors' actions on the revised manuscript:** The Introduction, Literature Review, Proposed Algorithm, Experimental Setup, Comparison With Recent Algorithms and CEC 2017 Competition Winners, Algorithm Complexity, Analysis of Parameter Settings, Conclusion, and Supporting Information have all been revised. New references, experimental comparisons, numerical tables, ECDFs, convergence figures, and reproducibility details have been added, and the presentation and justification of the algorithmic parameters have been clarified as described in the following responses.

> ***Comment 2:*** *The underlying principle may be of interest, although it is not entirely novel (see, for example, a keyword search for "Self-adaptive Differential Evolution with Lévy Mutation"). You may have a look at Das and Suganthan, "Differential Evolution: A Survey of the State-of-the-Art," and Beyer and Schwefel, "Evolution Strategies–A Comprehensive Introduction."*

**Authors' response:** We thank the Reviewer for this important observation and agree that the broad use of Lévy distributions, heavy-tailed mutation, and adaptive variation in evolutionary optimization is well established. We have therefore revised the manuscript so that it does not present the generic use of a Lévy distribution as the principal contribution.

The revised manuscript now distinguishes ALP from representative previous Lévy-based DE mechanisms according to **what is adapted and where the adaptation acts**. For example:

- Lévy DE uses Lévy distributions to generate or adapt a conventional DE control parameter, particularly the mutation scale factor $F$.
- Lévy Flight DE employs Lévy flight as an additional local-search mechanism around promising solutions.
- Bernstein–Lévy DE combines a Lévy-based search mechanism with elitist mutation and a specialized Bernstein-polynomial crossover strategy.
- In contrast, ALP directly adapts the **tail shape of the perturbation distribution itself**. At generation $g$, each individual receives a symmetric $\alpha$-stable distribution with stability index $\alpha_i^g$ determined by its fitness rank. At the same time, the dimension-wise probability $JR_j^g$ independently determines where and how frequently perturbations are activated.

Thus, the distinction of ALP is the separation of two controls: $JR_j^g$ governs coordinate-wise perturbation activation, whereas $\alpha_i^g$ governs individual-wise tail heaviness. Higher-ranked individuals receive lighter-tailed perturbations for refinement, while lower-ranked individuals receive heavier-tailed perturbations for more exploratory moves. We now present this two-level control structure, rather than the use of Lévy distributions alone, as the main methodological contribution.

**Authors' actions on the revised manuscript:** We expanded the Literature Review by adding a dedicated subsection entitled **"Lévy-based and heavy-tailed variation"** and a revised **"Positioning of this work"** subsection. The revised discussion now includes:

- Das and Suganthan's survey of adaptive and self-adaptive DE methods;
- Beyer and Schwefel's comprehensive treatment of adaptation in evolution strategies;
- Lévy DE by He and Yang;
- Lévy Flight DE by Sharma et al.; and
- Bernstein–Lévy differential evolution by Civicioglu and Besdok.

We also revised the Introduction and contribution statements and added Table 1, which explicitly contrasts CP, ADCP, and ALP in terms of perturbation probability, perturbation distribution, and individual-level tail control.

> ***Comment 3:*** *The top three winners, which you do not cite, are EBOwithCMAR, jSO, and LSHADE-SPACMA. Please compare your L-SRTDE-ALP to them. You could also remove many of the eleven DE-based methods you consider, as they are not particularly competitive. Quantity should not be confused with quality.*

**Authors' response:** We thank the Reviewer for identifying these important comparison algorithms. We agree that competition-level methods provide more informative reference points than simply increasing the number of comparatively weak baselines.

Following the Reviewer's recommendation, we incorporated EBOwithCMAR, jSO, and LSHADE-SPACMA into the revised comparison using the publicly available run-level results released for the CEC 2017 benchmark. These data were obtained from the public CEC 2017 repository maintained by P. N. Suganthan and colleagues:

https://github.com/P-N-Suganthan/CEC2017-BoundContrained.

The publicly released results contain the CEC 2017 experimental outcomes under the official evaluation protocol, including 51 independent runs for the dimensionalities considered in this study. Thus, the comparison uses the original competition results rather than our own reimplementations of these three algorithms.

To prioritize the quality of the comparison set rather than merely increasing its size, we reorganized the baseline set. In particular, the earlier comparisons with NL-SHADE-LBC, NL-SHADE-RSP, and iLSHADE-RSP were replaced by the three CEC 2017 methods recommended by the Reviewer. We retained L-SRTDE because it is the direct algorithmic baseline of the proposed method. We also retained seven recent DE variants because they provide relevant contemporary reference points, several of which employ Cauchy-based perturbation mechanisms closely related to the motivation of this study.

The new results show that L-SRTDE-ALP achieves the following win/loss/tie outcomes:

- against EBOwithCMAR: 18/2/9, 22/3/4, and 25/3/1 at $D = 30$, 50, and 100, respectively;
- against jSO: 23/1/5, 24/2/3, and 24/4/1; and
- against LSHADE-SPACMA: 22/2/5, 21/3/5, and 22/6/1.

L-SRTDE-ALP also obtains the lowest Friedman average rank at all three dimensionalities: 2.793 at $D = 30$, 2.483 at $D = 50$, and 2.948 at $D = 100$. At the same time, the revised manuscript explicitly acknowledges that the post-hoc procedures do not distinguish L-SRTDE-ALP significantly from its direct baseline, L-SRTDE. We have therefore avoided claiming uniform superiority.

**Authors' actions on the revised manuscript:** We revised the **Compared algorithms** paragraph and Table 2 to include EBOwithCMAR, jSO, and LSHADE-SPACMA and their recommended parameter settings. We also added their original publications to the reference list.

The subsection **"Comparison With Recent Algorithms and CEC 2017 Competition Winners"** was extensively revised. The new results are reported in:

- Tables 3, 5, and 7: Wilcoxon win/loss/tie summaries;
- Tables 4, 6, and 8: Bonferroni–Dunn-, Holm-, and Hochberg-adjusted post-hoc comparisons;
- Figs. 1–3: Friedman average rankings;
- Figs. 4–6: convergence curves;
- Figs. 7–9: ECDFs; and
- S1–S6 Tables in the Supporting Information: complete function-level numerical results.

> ***Comment 4:*** *Please provide more comprehensive results. Reporting only the mean and standard deviation is not particularly informative. The distribution of the results may not be symmetric, and readers are interested in knowing the best result that an algorithm is capable of achieving. At the very least, the tables should report the best, median, and standard deviation. An even more informative presentation would be to provide the cumulative distribution functions, based on, say, 100 independent runs. This would allow readers to estimate the probability of obtaining a result below any given threshold.*

**Authors' response:** We agree with the Reviewer that mean and standard deviation alone are insufficient for characterizing stochastic optimization results, especially when the distributions may be asymmetric or multimodal. We have therefore substantially expanded both the numerical and distributional reporting.

For each algorithm, function, and dimensionality, the revised Supporting Information now reports:

- the best objective error observed across the independent runs;

- the median objective error;
- the mean objective error;
- the standard deviation; and
- the outcome of a two-sided Wilcoxon rank-sum comparison against L-SRTDE-ALP.

We have also added empirical cumulative distribution functions (ECDFs), which are the empirical form of the CDF requested by the Reviewer. Each ECDF is constructed from all run-level final objective errors, thereby allowing the reader to assess the probability of attaining an error below any given threshold.

We retained 51 independent runs because this is the standard CEC 2017 evaluation protocol and preserves direct comparability with the official benchmark results and previous CEC 2017 studies. All 51 run-level observations were used to construct the ECDFs.

In addition, in response to the Reviewer's concern regarding availability of the data underlying the reported summary statistics, we have publicly released the author-generated run-level experimental data at https://github.com/gry-kiu/L-SRTDE-ALP. These data allow the reported means, standard deviations, medians, best values, and statistical comparisons to be independently inspected and reproduced.

For EBOwithCMAR, jSO, and LSHADE-SPACMA, the corresponding run-level data are already publicly available from the original CEC 2017 benchmark repository, and the exact source is documented in our repository.

In addition, the revised manuscript reports median best-so-far convergence curves, Friedman rankings, and multiplicity-adjusted post-hoc comparisons. These results provide complementary information on final performance, run-to-run variability, convergence behavior, and overall algorithm ranking.

**Authors' actions on the revised manuscript:** We added complete numerical and distributional results as follows:

- S1, S3, and S5 Tables report mean, standard deviation, and Wilcoxon outcomes for all compared algorithms at $D = 30$, 50, and 100;
- S2, S4, and S6 Tables report best and median objective errors;
- S7–S12 Tables provide the same statistics for the controlled L-SRTDE-ALP, L-SRTDE-ADCP, and L-SRTDE-CP comparisons;
- Figs. 7–9 in the main manuscript show ECDFs for six selected functions;
- Figs. D–F in the Supporting Information show ECDFs for the remaining 23 functions; and
- Figs. 4–6 and Figs. A–C provide median convergence curves across all 29 evaluated functions.

The captions and Supporting Information description were also revised to state explicitly that the statistics and ECDFs are based on 51 independent runs.

> ***Comment 5:*** *The algorithm uses 13 parameters, together with six additional hard-coded ones in formulae 15, 16, and 17, without sufficient justification, apart from $\alpha_{\min}$ and $\alpha_{\max}$. Moreover, the pseudo-random number generator and its seed are not considered. You may wish to consult Antunes (2025) regarding the influence of random-number quality.*

**Authors' response:** We thank the Reviewer for raising this important issue. We agree that the original presentation could give the impression that all listed quantities were newly introduced and independently tuned for L-SRTDE-ALP. This was not the case, and we have revised the manuscript to make the provenance and role of each setting explicit.

The parameter settings can be divided into three groups.

1. **Parameters inherited from L-SRTDE.** The initial population size $N_{\text{init}} = 20D$, minimum population size $N_{\min} = 4$, memory size $H = 5$, rank-selection pressure $k_p = 3$, initial crossover memory $M_{CR}^{(0)} = 1$, initial success rate $SR^{(0)} = 0.5$, and all fixed numerical constants in Eqs. (15)–(17) are retained unchanged from the original L-SRTDE framework. They were not retuned for the proposed method.

2. **Parameters inherited from ADCP.** The dimension-wise perturbation settings $JR_{\min} = 0.1$, $JR_{\max} = 0.2$, $\kappa = 10$, and the perturbation scale $c = 0.1$ are retained unchanged from ADCP. These settings were also fixed a priori and were not optimized for L-SRTDE-ALP.

3. **Parameters introduced by ALP.** The only additional parameters introduced by the proposed rank-conditioned tail-shape mechanism are $\alpha_{\min}$ and $\alpha_{\max}$, which define the range of individual-specific stability indices.

We have also clarified the notation used by the algorithm. In particular, $n_c$ is not a tunable algorithmic parameter; it is an internal rotating insertion pointer initialized to 1 for bookkeeping. The nonlinearity parameter used in the dimension-wise jumping-rate mapping is now denoted by $\kappa$, thereby separating it from internal indices, and its ADCP-recommended value is reported as $\kappa = 10$.

To examine the only two ALP-specific parameters, we evaluated 20 combinations defined by $\alpha_{\min} \in \{0.3, 0.5, 0.7, 1.0\}$ and $\alpha_{\max} \in \{1.0, 1.3, 1.5, 1.7, 2.0\}$ on the complete 50-dimensional CEC 2017 test set. The Friedman test and Iman–Davenport correction detected no statistically significant differences among these settings ($\chi_F^2 = 23.561$, $p = 0.214$; $F_F = 1.251$, $p = 0.211$), and Kendall's $W \approx 0.043$ indicated only weak rank concordance. Pairwise and post-hoc comparisons likewise identified no significant differences after family-wise error correction. These results indicate that the method is not highly sensitive to the investigated stability-index ranges.

We also agree that the PRNG and seed-generation procedure are important controlled settings. In response to the concern and the recommended reference, the revised manuscript now states that all stochastic algorithms use the C++ `std::mt19937` Mersenne Twister generator. Following the time-based seeding convention used in the CEC 2017 protocol, each independent run initializes a global seed

$$s = \texttt{unsigned(time(NULL))}.$$

Four separate Mersenne Twister instances are then initialized using $s$, $s + 100$, $s + 200$, and $s + 300$ for the different random-variable generators. Because this protocol uses time-based initialization, there is no single fixed seed shared by all runs; instead, the complete seed-generation rule is now explicitly reported. The same PRNG and seeding procedure were applied to every compared algorithm so that the random-number mechanism was controlled consistently across the study.

**Authors' actions on the revised manuscript:** We revised the **Compared algorithms** paragraph and Table 2 to distinguish parameters inherited from L-SRTDE and ADCP from the two parameters newly introduced by ALP. Equation (7), Algorithm 1, and Algorithm 2 were also revised to use the unambiguous notation $\kappa$ and to distinguish tunable settings from internal bookkeeping pointers.

The existing subsection entitled "Analysis of Parameter Settings," which was already included in the original submission, has been retained to support the justification of the two ALP-specific parameters. We revised the surrounding parameter discussion to distinguish the inherited L-SRTDE and ADCP settings from $\alpha_{min}$ and $\alpha_{max}$.

We also added a dedicated **"Random-number generation"** paragraph to the Experimental Setup, specifying the PRNG, global seed-generation rule, four derived random streams, and the fact that the same procedure was used for all algorithms.

> ***Comment 6:*** *The quality of the figures on pages 26–28 is poor, at least in the PDF I downloaded.*

**Authors' response:** We thank the Reviewer for pointing this out and agree that the original multi-panel figures were too dense and difficult to inspect at normal magnification.

We have regenerated the convergence figures at publication quality and reorganized their presentation. Six selected functions are now displayed as separate, readable multi-panel figures in the main manuscript, while the remaining 23

functions are provided in the Supporting Information. We used consistent line styles, markers, axis labels, legends, and enlarged graphical elements to improve discrimination among the twelve algorithms.

We also added ECDF figures using the same visual organization. This separation keeps the principal comparisons readable in the main manuscript while preserving complete function-level graphical results in the Supporting Information.

**Authors' actions on the revised manuscript:** The original convergence figures have been replaced by:

- Figs. 4–6 in the main manuscript, showing median convergence curves for six selected functions at $D = 30$, 50, and 100;
- Figs. A–C in the Supporting Information, showing the corresponding curves for the remaining 23 functions;
- Figs. 7–9 in the main manuscript, showing ECDFs for the six selected functions; and
- Figs. D–F in the Supporting Information, showing ECDFs for the remaining functions.

All revised figures have been re-exported as separate high-resolution files suitable for journal submission. We believe that the revised organization and graphical quality substantially improve readability.
