# Supplementary material for: A rank-conditioned α-stable perturbation for differential evolution: Separating perturbation frequency and tail heaviness
Source: PLoS One. 2026 Sep 9;21(9):e0357907. doi: 10.1371/journal.pone.0357907 (PMC13557417; doi:10.1371/journal.pone.0357907)
Supplement: Peer review history

# Peer review: Decision Letter 0

**DOI:** 10.1371/journal.pone.0357907.r001

**Authors:** Mahamed G.H. Omran

4 Aug 2026

Dear Dr. An,

publication criteria and not, for example, on novelty or perceived impact.

For Lab, Study and Registered Report Protocols: These article types are not expected to include results but may include pilot data.

==============================

Please submit your revised manuscript by Oct 03 2026 11:59PM. If you will need more time than this to complete your revisions, please reply to this message or contact the journal office at plosone@plos.org. When you're ready to submit your revision, log on to https://www.editorialmanager.com/pone/ and select the 'Submissions Needing Revision' folder to locate your manuscript file.

- A letter that responds to each point raised by the academic editor and reviewer(s). You should upload this letter as a separate file labeled 'Response to Reviewers'.
- A marked-up copy of your manuscript that highlights changes made to the original version. You should upload this as a separate file labeled 'Revised Manuscript with Track Changes'.
- An unmarked version of your revised paper without tracked changes. You should upload this as a separate file labeled 'Manuscript'.

If you would like to make changes to your financial disclosure, please include your updated statement in your cover letter. Guidelines for resubmitting your figure files are available below the reviewer comments at the end of this letter.

If applicable, we recommend that you deposit your laboratory protocols in protocols.io to enhance the reproducibility of your results. Protocols.io assigns your protocol its own identifier (DOI) so that it can be cited independently in the future. For instructions see: https://journals.plos.org/plosone/s/submission-guidelines#loc-laboratory-protocols. Additionally, PLOS ONE offers an option for publishing peer-reviewed Lab Protocol articles, which describe protocols hosted on protocols.io. Read more information on sharing protocols at https://plos.org/protocols?utm_medium=editorial-email&utm_source=authorletters&utm_campaign=protocols.

As the corresponding author, your ORCID iD is verified in the submission system and will appear in the published article. PLOS supports the use of ORCID, and we encourage all coauthors to register for an ORCID iD and use it as well. Please encourage your coauthors to verify their ORCID iD within the submission system before final acceptance, as unverified ORCID iDs will not appear in the published article. *Only*  the individual author can complete the verification step; PLOS staff *cannot*  verify ORCID iDs on behalf of authors.

We look forward to receiving your revised manuscript.

Kind regards,

Mahamed G.H. Omran

Academic Editor

PLOS One

Journal Requirements:

When submitting your revision, we need you to address these additional requirements.

1. Please ensure that your manuscript meets PLOS ONE's style requirements, including those for file naming. The PLOS ONE style templates can be found at

https://journals.plos.org/plosone/s/file?id=wjVg/PLOSOne_formatting_sample_main_body.pdf and

https://journals.plos.org/plosone/s/file?id=ba62/PLOSOne_formatting_sample_title_authors_affiliations.pdf

2. Please note that PLOS One has specific guidelines on code sharing for submissions in which author-generated code underpins the findings in the manuscript. In these cases, we expect all author-generated code to be made available without restrictions upon publication of the work. Please review our guidelines at https://journals.plos.org/plosone/s/materials-and-software-sharing#loc-sharing-code and ensure that your code is shared in a way that follows best practice and facilitates reproducibility and reuse.

3. We note that the grant information you provided in the ‘Funding Information’ and ‘Financial Disclosure’ sections do not match.

When you resubmit, please ensure that you provide the correct grant numbers for the awards you received for your study in the ‘Funding Information’ section.

4. Thank you for stating the following financial disclosure:

“This work was supported by the National Research Foundation of Korea (NRF) grant funded by the Korea government (MSIT) (No. RS-2023-00214326, No. RS-2026-25490120 and No. RS-2023-00242528).”

Please state what role the funders took in the study.  If the funders had no role, please state: "The funders had no role in study design, data collection and analysis, decision to publish, or preparation of the manuscript."

If this statement is not correct you must amend it as needed.

Please include this amended Role of Funder statement in your cover letter; we will change the online submission form on your behalf.

5. Thank you for stating the following in the Competing Interests section:

“Authors Tae Jong Choi and Yeji An are spouses. The authors declare that this relationship did not influence the conduct or reporting of this study and that they have no competing financial interests.”

Please confirm that this does not alter your adherence to all PLOS ONE policies on sharing data and materials, by including the following statement: "This does not alter our adherence to  PLOS ONE policies on sharing data and materials.” (as detailed online in our guide for authors http://journals.plos.org/plosone/s/competing-interests).  If there are restrictions on sharing of data and/or materials, please state these. Please note that we cannot proceed with consideration of your article until this information has been declared.

Please include your updated Competing Interests statement in your cover letter; we will change the online submission form on your behalf.

6. When completing the data availability statement of the submission form, you indicated that you will make your data available on acceptance. We strongly recommend all authors decide on a data sharing plan before acceptance, as the process can be lengthy and hold up publication timelines. Please note that, though access restrictions are acceptable now, your entire data will need to be made freely accessible if your manuscript is accepted for publication. This policy applies to all data except where public deposition would breach compliance with the protocol approved by your research ethics board. If you are unable to adhere to our open data policy, please kindly revise your statement to explain your reasoning and we will seek the editor's input on an exemption. Please be assured that, once you have provided your new statement, the assessment of your exemption will not hold up the peer review process.

7. Please amend either the title on the online submission form (via Edit Submission) or the title in the manuscript so that they are identical.

8. Please amend either the abstract on the online submission form (via Edit Submission) or the abstract in the manuscript so that they are identical.

9. If the reviewer comments include a recommendation to cite specific previously published works, please review and evaluate these publications to determine whether they are relevant and should be cited. There is no requirement to cite these works unless the editor has indicated otherwise.

[Note: HTML markup is below. Please do not edit.]

Reviewers' comments:

Reviewer's Responses to Questions

**Comments to the Author**

1. Is the manuscript technically sound, and do the data support the conclusions?

Reviewer #1: Partly

**********

2. Has the statistical analysis been performed appropriately and rigorously?

Reviewer #1: Yes

**********

3. Have the authors made all data underlying the findings in their manuscript fully available?

The PLOS Data policy

Reviewer #1: No

**********

4. Is the manuscript presented in an intelligible fashion and written in standard English?

Reviewer #1: Yes

**********

Reviewer #1: The underlying principle may be of interest, although it is not entirely novel (see, for example, a keyword search for "Self-adaptive Differential Evolution with Lévy Mutation").

The algorithm introduces too many parameters without providing any justification for their choice.

The experimental results are not sufficiently comprehensive to support the claimed comparisons.

There are also no comparisons with the winners of the CEC 2017 competition.

Overall, the proposed algorithm, in its present form, is not convincing.

----------------------- More detailed remarks

p. 2

"We conduct an extensive empirical study on the IEEE CEC 2017 benchmark suite across multiple dimensions, including comparisons with state-of-the-art DE variants and controlled comparisons with CP and ADCP."

The top three winners, which you do not cite, are

EBOwithCMAR

Kumar, A., Misra, R. K., & Singh, D. (2017). "Improving the local search capability of Effective Butterfly Optimizer using Covariance Matrix Adapted Retreat Phase." In 2017 IEEE Congress on Evolutionary Computation (CEC), pp. 1835–1842.

jSO

Brest, J., Maučec, M. S., & Bošković, B. (2017). "Single objective real-parameter optimization: algorithm jSO." In 2017 IEEE Congress on Evolutionary Computation (CEC).

LSHADE-SPACMA

Mohamed, A. W., Hadi, A. A., Fattouh, A. M., & Jambi, K. M. (2017). "LSHADE with semi-parameter adaptation hybrid with CMA-ES for solving CEC 2017 benchmark problems." In 2017 IEEE Congress on Evolutionary Computation (CEC), pp. 145–152.

Please compare your L-SRTDE-ALP to them.

And you could remove many of the eleven DE-based methods you consider, for they are not very competitive. Quantity does not mean quality.

Here's a polished version in natural British English:

You could also remove many of the eleven DE-based methods you consider, as they are not particularly competitive. Quantity should not be confused with quality.

In addition, please provide more comprehensive results. Reporting only the mean and standard deviation, as in Tables S1–S4, is not particularly informative. First, the distribution of the results may not be symmetric. Second, readers are interested in knowing the best result that an algorithm is capable of achieving. At the very least, the tables should report the best, median, and standard deviation.

An even more informative presentation would be to provide the cumulative distribution functions (CDFs), based on, say, 100 independent runs. This would allow readers to estimate the probability of obtaining a result below any given threshold.

p. 6

"This section introduces ALP, a perturbation operator for DE recombination that assigns distinct tail behaviors to individual solutions"

You may have a look at

Das & Suganthan, "Differential Evolution: A Survey of the State-of-the-Art," IEEE Transactions on Evolutionary Computation*, 15(1), 2011.

It reviews self-adaptive parameter schemes and Lévy variants.

Beyer & Schwefel, Evolution Strategies – A Comprehensive Introduction, Natural Computing, 1, 2002.

p. 11

"Ninit = 20D, Nmin = 4, H = 5, k = 1, nc = 1, kp = 3, MCr,r = 1, SR = 0.5, JRmin = 0.1, JRmax = 0.2, αmin = 0.3, αmax = 1.7"

13 parameters, together with six additional hard-coded ones (formulae 15, 16, and 17), are used without any justification, apart αmin and αmax. This is far from sufficient.

Moreover, you do not consider the two other parameters: the pseudo-random number generator (PRNG) and its seed.

You may wish to consult the following paper, which cites several studies on the influence of these parameters:

Antunes, Benjamin A. 2025. ‘The Influence of the Random Numbers Quality on the Results in Stochastic Simulations and Machine Learning’. arXiv:2510.25269. Preprint, arXiv, October 29. https://doi.org/10.48550/arXiv.2510.25269.

Minor point

----------

The quality of the figures on pages 26–28 is poor, at least in the PDF I downloaded.

**********

what does this mean?). If published, this will include your full peer review and any attached files.

If you choose “no”, your identity will remain anonymous but your review may still be made public.

**Do you want your identity to be public for this peer review?**  For information about this choice, including consent withdrawal, please see our Privacy Policy

Reviewer #1: No

**********

[NOTE: If reviewer comments were submitted as an attachment file, they will be attached to this email and accessible via the submission site. Please log into your account, locate the manuscript record, and check for the action link "View Attachments". If this link does not appear, there are no attachment files.]

To ensure your figures meet our technical requirements, please review our figure guidelines: https://journals.plos.org/plosone/s/figures

You may also use PLOS’s free figure tool, NAAS, to help you prepare publication quality figures: https://journals.plos.org/plosone/s/figures#loc-tools-for-figure-preparation.

NAAS will assess whether your figures meet our technical requirements by comparing each figure against our figure specifications.

# Author comment: Author response to Decision Letter 1

**DOI:** 10.1371/journal.pone.0357907.r002

18 Aug 2026

Please refer to the separately uploaded “Response to Reviewers” file, which provides our complete point-by-point responses to all comments raised by the Academic Editor and Reviewer 1, together with the corresponding revisions made to the manuscript and Supporting Information.

Attachment Submitted filename: Response to reviewers.pdf

# Peer review: Decision Letter 1

**DOI:** 10.1371/journal.pone.0357907.r003

**Authors:** Mahamed G.H. Omran

24 Aug 2026

A rank-conditioned α-stable perturbation for differential evolution: Separating perturbation frequency and tail heaviness

PONE-D-26-37177R1

Dear Dr. An,

We’re pleased to inform you that your manuscript has been judged scientifically suitable for publication and will be formally accepted for publication once it meets all outstanding technical requirements.

Within one week, you’ll receive an e-mail detailing the required amendments. When these have been addressed, you’ll receive a formal acceptance letter and your manuscript will be scheduled for publication.

An invoice will be generated when your article is formally accepted. Please note, if your institution has a publishing partnership with PLOS and your article meets the relevant criteria, all or part of your publication costs will be covered. Please make sure your user information is up-to-date by logging into Editorial Manager at Editorial Manager® and clicking the ‘Update My Information' link at the top of the page. For questions related to billing, please contact billing support.

If your institution or institutions have a press office, please notify them about your upcoming paper to help maximize its impact. If they’ll be preparing press materials, please inform our press team as soon as possible -- no later than 48 hours after receiving the formal acceptance. Your manuscript will remain under strict press embargo until 2 pm Eastern Time on the date of publication. For more information, please contact onepress@plos.org.

Kind regards,

Mahamed G.H. Omran

Academic Editor

PLOS One

Additional Editor Comments (optional):

Reviewers' comments:

Reviewer's Responses to Questions

**Comments to the Author**

Reviewer #1: All comments have been addressed

**********

2. Is the manuscript technically sound, and do the data support the conclusions?

Reviewer #1: (No Response)

**********

3. Has the statistical analysis been performed appropriately and rigorously?

Reviewer #1: (No Response)

**********

4. Have the authors made all data underlying the findings in their manuscript fully available?

The PLOS Data policy

Reviewer #1: (No Response)

**********

5. Is the manuscript presented in an intelligible fashion and written in standard English?

Reviewer #1: (No Response)

**********

Reviewer #1: (No Response)

**********

what does this mean?). If published, this will include your full peer review and any attached files.

If you choose “no”, your identity will remain anonymous but your review may still be made public.

**Do you want your identity to be public for this peer review?**  For information about this choice, including consent withdrawal, please see our Privacy Policy

Reviewer #1: No

**********

# Editor report: Acceptance letter

**DOI:** 10.1371/journal.pone.0357907.r004

**Authors:** Mahamed G.H. Omran

PONE-D-26-37177R1

PLOS One

Dear Dr. An,

I'm pleased to inform you that your manuscript has been deemed suitable for publication in PLOS One. Congratulations! Your manuscript is now being handed over to our production team.

At this stage, our production department will prepare your paper for publication. This includes ensuring the following:

* All references, tables, and figures are properly cited

* All relevant supporting information is included in the manuscript submission,

* There are no issues that prevent the paper from being properly typeset

You will receive further instructions from the production team, including instructions on how to review your proof when it is ready. Please keep in mind that we are working through a large volume of accepted articles, so please give us a few days to review your paper and let you know the next and final steps.

Lastly, if your institution or institutions have a press office, please let them know about your upcoming paper now to help maximize its impact. If they'll be preparing press materials, please inform our press team within the next 48 hours. Your manuscript will remain under strict press embargo until 2 pm Eastern Time on the date of publication. For more information, please contact onepress@plos.org.

You will receive an invoice from PLOS for your publication fee after your manuscript has reached the completed accept phase. If you receive an email requesting payment before acceptance or for any other service, this may be a phishing scheme. Learn how to identify phishing emails and protect your accounts at https://explore.plos.org/phishing.

If we can help with anything else, please email us at customercare@plos.org.

Thank you for submitting your work to PLOS One and supporting open access.

Kind regards,

PLOS One Editorial Office Staff

on behalf of

Prof. Mahamed G.H. Omran

Academic Editor

PLOS One
